# Puerarin relives inflammation, bone destruction and facilitates osteogenic differentiation in periodontitis by enhancing mitochondrial autophagy via activating mitochondrial Mitofusin 2

**DOI:** 10.1186/s13287-025-04355-w

**Published:** 2025-05-01

**Authors:** Yulan Xiang, Zelu Li, Xin He, Xiaoyang Chu, Chunyan Gao, Jiahao Guo, Yingyi Luan, Kai Yang, Dongliang Zhang

**Affiliations:** 1 Department of Orthodontics, Beijing Stomatological Hospital, School of Stomatology, Capital Medical University, Capital Medical University, Beijing, China; 2 Department of Stomatology, Fifth Medical Center of Chinese PLA General Hospital, Beijing, China; 3 Weifang Medical College, Weifang, Shandong China; 4 Beijing Obstetrics and Gynecology Hospital, Capital Medical University, Beijing, China; 5 Translational Medical Center, Weifang Second People’s Hospital, Shandong Second Medical University, Weifang, Shandong China

**Keywords:** Periodontitis, Puerarin, Inflammation, Mitochondrial autophagy, Mfn2

## Abstract

**Purpose:**

Puerarin (Pue) has recently been reported to have therapeutic effects on periodontitis (PD). However, there is insufficient evidence, and the mechanism involved has not yet been revealed. This work delved to explore the exact therapeutic effects and molecular mechanism of Pue in treating PD.

**Methods:**

PD mouse (C57BL/6 N mouse) model constructed by *Porphyromonas gingivalis*-lipopolysaccharide (Pg-LPS) induction was treated with Pue. Therapeutic efficacy of Pue for PD was examined by a series of experiments. PD cell model was induced by treating human periodontal ligament cells with Pg-LPS. Therapeutic effects of Pue on PD cell model, along with the potential molecular mechanism, were explored by logical experiments. Rescue experiments based on in vitro and in vivo studies were implemented to validate the molecular mechanism of Pue in treating PD.

**Results:**

In PD mice, Pue treatment relieved inflammation and bone destruction, facilitated osteogenic differentiation and autophagy in periapical tissues. In PD cell model, Pue treatment facilitated osteogenic differentiation and mitochondrial autophagy; suppressed inflammation and mitochondrial reactive oxygen species; maintained mitochondrial membrane potential and mitochondrial kinetic homeostasis; and activated mitochondrial Mitofusin 2 (Mfn2). However, these influences of Pue on PD cell model were eliminated by CsA (mitochondrial autophagy inhibitor). The enhanced mitochondrial autophagy induced by Pue was reversed by Mfn2 silencing. Through in vivo data, Mfn2 knockdown counteracted the therapeutic effects of Pue on PD mice.

**Conclusion:**

Pue exerted therapeutic effects on PD, possibly by enhancing mitochondrial autophagy via activating mitochondrial Mfn2. This might be a cure for PD.

**Graphical Abstract:**

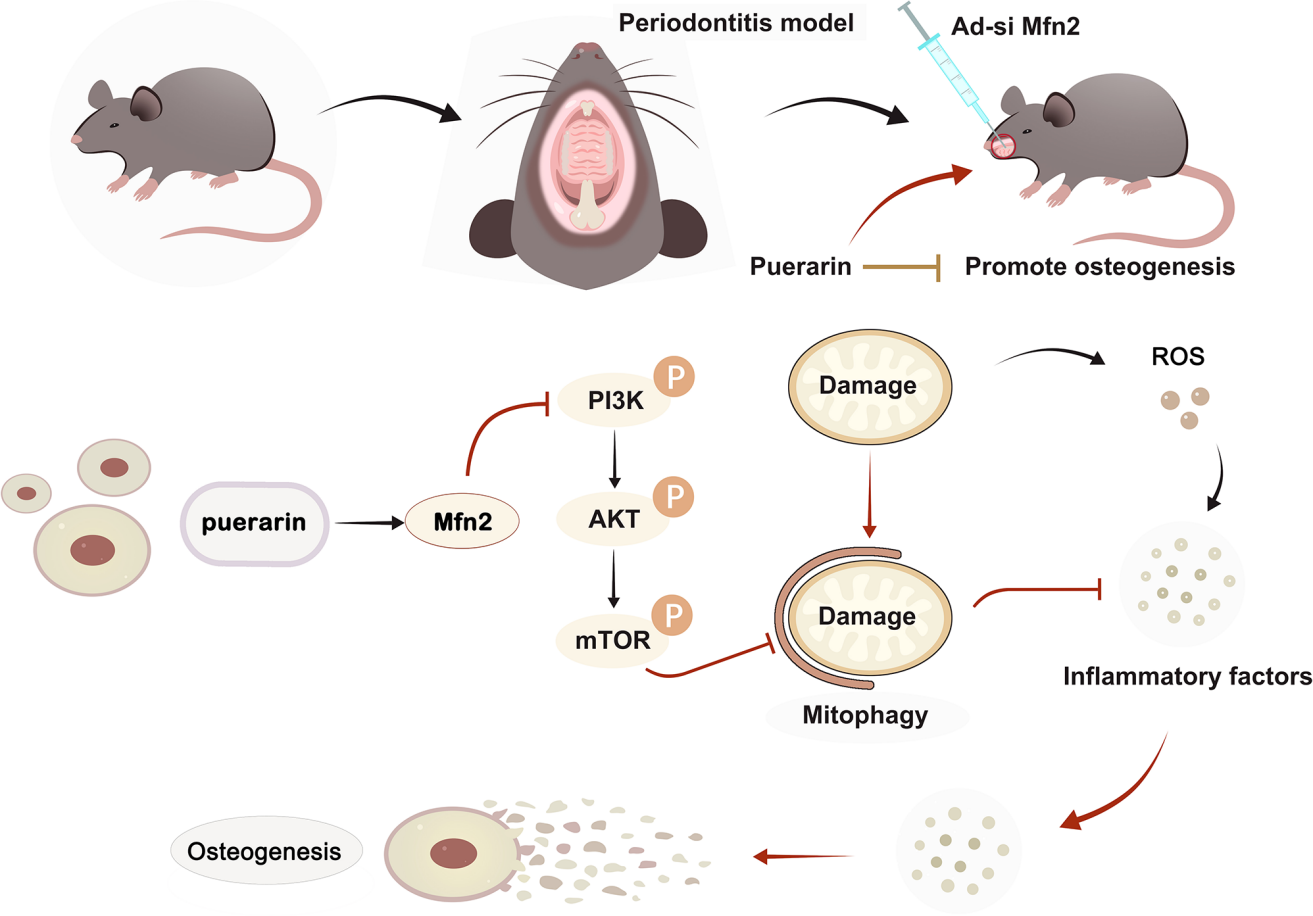

**Supplementary Information:**

The online version contains supplementary material available at 10.1186/s13287-025-04355-w.

## Introduction

Periodontitis (PD) is a common chronic disease globally that stems from periodontal infection and pathogen invasion. It is typically characterized by inflammation and destruction in the periodontal tissues and resorption of alveolar bone [1]. PD is not only a prominent cause of tooth loss, but is also associated with a variety of systemic diseases [2]. The treatment of PD includes surgical and pharmacological treatments, aimed at controlling inflammation, promoting osteogenic differentiation and halting disease progression [3]. Nevertheless, the current treatments are still unsatisfactory, and finding effective treatment strategies is critical to improving PD.

Puerarin (Pue), also known as daidzein-8-c-glucoside (7,4’-dihydroxy-8-c-glycosylisoflavone, C21H20O9), is a natural flavonoid compound extracted from the traditional Chinese medicine Pueraria lobata; it possesses multiple pharmacological activities, such as anti-inflammatory, anti-cardiovascular disease, anti-ischemia-reperfusion injury, anti-oxidative stress, neuroprotective effect, anti-osteoporosis and reduction of insulin resistance [4, 5]. Pue is used in treating a wide range of diseases due to its unique advantages such as low toxicity, high safety, few side effects and low cost [6]. Recent study has highlighted the therapeutic potential of Pue to treat PD, as the anti-inflammatory and antioxidant effects make it effective in treating mice with PD [7]. Pue has been discovered to be effective in suppressing the formation of osteoclast and the loss of bone, which is thus proposed to be an effective agent for the treatment of bone-related diseases such as PD [8, 9]. As we know, human periodontal ligament cells (hPDLCs) is capable of inducing osteogenic differentiation and bone regeneration, whereas its damage can result in the progression and poor treatment effectiveness of PD [10]. Interestingly, Pue has been emphasized to promote proliferation and osteogenic differentiation of hPDLCs, and meanwhile, it reduces alveolar bone loss and relieves bone loss and collagen destruction in PD rats. These findings provide an important rationale for the application of Pue in the clinical treatment of PD [11, 12]. Unfortunately, more data is currently unavailable when it comes to Pue to treat PD. This paper was dedicated to exploring in detail the therapeutic effects of Pue on PD through in vitro and in vivo studies.

Mitochondria are the structural basis and energy source for osteogenic differentiation of stem cells, and mitochondrial homeostasis is necessary for achieving osteogenic differentiation of stem cells; in PD, microbial stimulation and inflammation-induced mitochondrial dysregulation, especially mitochondrial autophagy imbalance, is one of the most important reasons for the destruction of stem cell osteogenic differentiation ability; therefore, mitochondrial autophagy is considered a potential therapeutic target for bone-related diseases, including PD [13, 14]. Intriguingly, in the lipopolysaccharide (LPS)-induced cellular inflammation model, Pue has been noticed to be able to alleviate mitochondrial damage by facilitating mitochondrial autophagy [15]. Mitofusin 2 (Mfn2), mainly located on the outer mitochondrial membrane, is a critical factor involved in mitochondrial dynamics as it can enable mitochondrial fusion [16]. In periodontal disease, Mfn2 maintains cellular function and mitochondrial fusion, and its expression disruption leads to an imbalance in mitochondrial dynamics, as well as inflammation and mitochondrial dysfunction in periodontal tissues [17]. Recent study reported the critical regulatory role of Mfn2 in mitochondrial autophagy [18]. According to these discoveries, this paper focused on the effect on Pue on the Mfn2-mediated mitochondrial autophagy in PD to gain insights into its intrinsic mechanism in the treatment of PD.

## Materials and methods

### ARRIVE guidelines

The work has been reported in line with the ARRIVE guidelines 2.0.

### Animals and animal ethics committee

C57BL/6 N mice (10-week old, 20–25 g, *n* = 174) were commercially supplied by Vital River Laboratory Animal Technology (Beijing, China). All mice were kept in a specific pathogen-free room (a 12-h day/night cycle) at 22 ± 2 °C, and allowed freely to diet and water. Pue injection (Purity ≥ 98%) was purchased from Yuanye Biotechnology (B20446; Shanghai, China).

This experiment was approved by the Animal Welfare Ethics Committee of Beijing MDKN Biotechnology Co., LTD.(Approval number: MDKN-2023-048; Date of approval: 2022.3.25), and was conducted in strict accordance with the experimental animal care and use guidelines of Beijing Animal Control Committee.

### Animal PD modeling, Pue treatment and adenovirus transfection

The PD mouse model was constructed as previously reported [19]. Briefly, mice were anaesthetized with 2% isoflurane and then fixed in the supine position on the operating table. A 5 − 0 silk thread that had been wetted with *Porphyromonas gingivalis*-lipopolysaccharide (Pg-LPS) (10 µg/mL) were knotted and placed between the first and second molar spaces on the right side of the maxilla. The silk thread was cut at the outside of the knot so that the knot was located buccolingually between the first and second molar spaces. *Porphyromonas gingivalis* was purchased from Ningbo Biotechnology (B290826; Ningbo, China), while LPS was provided by Sigma (L2630; St. Louis, MO, USA).

In the following, Pue treatment of mice was performed using a microsyringe. The injection site was: the buccal gingival papilla between the maxillary right first and second molars and the top of the alveolar ridge on the palatal side. Pue at doses of 5 mg/kg and 10 mg/kg (diluted into 5 µL phosphate buffer solution) were injected, respectively. After injection, the microsyringe was kept in the injection site for 1 min to prevent the drug from flowing out. Pue treatment was given every two days.

Adenovirus transfection of mice was implemented as following: Adenovirus-based Mfn2 siRNA and Mfn2 siRNA negative control (NC) were provided by GeneChem (Shanghai, China), and individually injected into mice 7 days prior to PD modeling. The viral titer of Mfn2 siRNA adenovirus was 10^9^ VG/µL, and the injection volume was 500 nL [20]. The adenovirus injection site was the same as the Pue injection site.

### Animal grouping and specific treatment

The in vivo study contained two parts: Pue treatment efficacy study, and the following Pue treatment mechanism study.

In the Pue treatment efficacy study, 84 mice were randomly (by the random number table method) selected and divided into four groups: the Control group (*n* = 21), the PD group (*n* = 21), the PD + Pue 5 mg/kg treatment group (*n* = 21), and the PD + Pue 10 mg/kg treatment group (*n* = 21). Mice of the Control group were subjected to the similar treatment process as for the PD modeling, but the 5 − 0 silk thread was not wetted with Pg-LPS; mice of the PD group experienced the PD modeling; those of the PD + Pue 5 mg/kg treatment group and the PD + Pue 10 mg/kg treatment group firstly underwent the PD modeling, and then treated by Pue for 10 days.

Another 84 mice were utilized for Pue treatment mechanism study, which were randomly (by the random number table method) divided into the Control group (*n* = 21), the PD group (*n* = 21), the PD + Pue + siMfn2 NC group (*n* = 21), and the PD + Pue + siMfn2 group (*n* = 21). The treatment of mice in the Control group and the PD group were performed as described above. Those of the PD + Pue + siMfn2 NC group and the PD + Pue + siMfn2 group were firstly transfected by adenovirus-based Mfn2 siRNA NC and Mfn2 siRNA, respectively. After 7 days of transfection, the PD modeling and a 10-day Pue treatment were implemented.

After 10 days of Pue treatment, all mice were euthanized as follows: mice were placed in an airtight container and CO₂ (concentration gradient rising from 30 to 70%) was slowly injected into the airtight container; mice gradually lost consciousness and then died. The right maxilla of each mouse was collected for the subsequent experiment. Unsuccessful modeling or dead animals were excluded. In this study, all animals were modeled successfully and did not die. Therefore, no animals were excluded. All experimenters were aware of group assignments at the different stages of the experiment.

Of the 21 mice per group, six of them were used for Micro-computed tomography (Micro-CT) analysis, six for enzyme-linked immunosorbent assay (ELISA), three for histological studies (including hematoxylin-eosin [H&E] staining and immunohistochemistry), three for real-time quantitative reverse transcription-polymerase chain reaction (qRT-PCR), and three for Western blot. For each analysis, mice were randomly selected so there was no selection bias.

### In vivo safety testing of Pue

The remaining six mice were used for the in vivo safety testing of Pue. Briefly, the six mice were injected with 10 mg/kg Pue in periodontal tissues for 10 consecutive days. Then mice were euthanized by inhaling CO₂ (concentration gradient rising from 30 to 70%) in the airtight container. The heart, liver, spleen, lung, kinder, brain, skin and oral mucosa of each mice were collected immediately and stored at -80 °C for the observation of pathological changes by H&E staining.

### Sulcus bleeding index and tooth mobility

The sulcus bleeding index of mice was evaluated by a blunt-tipped periodontal probe. Measurement was taken at two sites, i.e., distal mesial buccal and palatal sides of the maxillary first molar. The assessment criteria were: 0 point, healthy gingiva with no bleeding on probing; 1 point, bleeding on probing, but no oedema or color change of the gingival papilla and marginal gingiva; 2 points, bleeding on probing, color change of the gingival papilla and marginal gingiva, but no oedema; 3 points, bleeding on probing, color change of the gingival papilla and marginal gingiva, as well as mild oedema; 4 points, bleeding on probing, color change of the gingival papilla and marginal gingiva, as well as marked oedema; 5 points, bleeding on probing, spontaneous bleeding and color change of the gingival papilla and marginal gingiva, as well as marked oedema.

The tooth mobility of the maxillary right first molar tooth was observed. Briefly, the tip of the dental forceps was used against the maxillofacial fossa, and then the dental forceps were rocked in a buccolingual or proximal-distal-medial direction. The tooth mobility was appraised according to the loosening direction. 0 point, normal physiological mobility of the tooth; 1 point, the tooth was loose in the buccal (labial) lingual direction; 2 points, the tooth was loose in the buccal (labiolingual) and proximal-distal-medial directions; 3 points, the tooth was loose in the buccal (labiolingual), proximal-distal-medial and vertical directions.

### Micro-CT analysis

The right maxilla samples of mice were placed in 4% paraformaldehyde (Biolab Technology, Beijing, China) for a 24-h fixation. Micro-CT scanning of the right maxilla was performed with the Skyscan 1176 X-ray microtomograph (Bruker, Karlsruhe, Germany). The scanning parameters were: voltage 70 kV, current 200 µA, exposure time 270 ms, rotation angle 360°, and scanning layer thickness 34.4 μm. Data were reconstructed by the Mimics software (Materialise, Leuven, Belgium) in order to obtain the three-dimensional images. The distance between the cementoenamel junction (CEJ) and the alveolar bone crest (ABC), as well as bone volume fraction (BV/TV), trabecular bone number (Tb. N), and trabecular spacing (Tb. Sp), were analyzed.

### H&E staining

The right maxilla samples were fixed by 4% paraformaldehyde, and then immersed into 10% ethylene-diamine tetra-acetic acid (EDTA) decalcifying solution for two weeks. Besides, the heart, liver, spleen, lung, kinder, brain, skin and oral mucosa of mice were subjected to fixation by 4% paraformaldehyde. After routine dehydration, the samples were embedded in paraffin to prepare Sect. (4 μm thickness). Routine dewaxing and rehydration were sequentially performed on the sections. In the following, 5 min staining by hematoxylin solution (Biolab Technology, Beijing, China) and then 2 min staining by eosin solution (Biolab Technology, Beijing, China) were implemented on the sections. Post routine dehydration and transparency, the sections were enclosed in neutral resin and placed under a light microscope (Olympus, Tokyo, Japan) for observation.

### Immunohistochemistry

The prepared sections of the right maxilla samples were routinely dewaxed and rehydrated. After boiled in citrate buffer for 10 min, the sections were blocked in normal goat serum (Beyotime Biotechnology, Shanghai, China) for 30 min at room temperature. Rabbit anti-RUNX2 (1:100, ab114133, Abcam, Cambridge, UK) and anti-LC3 (1:100, ab62721, Abcam, Cambridge, UK) primary antibodies were individually dropped onto the sections for 12 h probe at 4 °C, and then horseradish peroxidase (HRP) conjugated goat anti-rabbit secondary antibody (1:200, ab6721, Abcam, Cambridge, UK) was added to incubate the sections for 30 min at room temperature. Following 3,3′-diaminobenzidine (DAB) (Beyotime Biotechnology, Shanghai, China) color reaction and hematoxylin counterstaining, the sections were dried, transparent and disclosed in neutral resin. The expression of RUNX2 and LC3 was observed under a light microscope (Olympus, Tokyo, Japan). The qualification of RUNX2 and LC3 expression was finished by the Image J software (ImageJ, NIH, Bethesda, MD, USA).

### hPDLCs culture and identification

hPDLCs were purchased from Procell Life Science & Technology (Wuhan, China) (https://www.procell.com.cn/view/5779.html), and cultivated in Dulbecco’s modified Eagle’s medium (DMEM) (Beyotime Biotechnology, Shanghai, China) containing 1% penicillin/streptomycin (Beyotime Biotechnology, Shanghai, China) and 10% fetal bovine serum (FBS) (Beyotime Biotechnology, Shanghai, China) at 37 °C, 5% CO_2_. The original source of hPDLCs (Procell Life Science & Technology, Wuhan, China) has confirmed that there was initial ethical approval for collection of human cells, and that the donors had signed informed consent.

hPDLCs were sub-cultured three times, and flow cytometry was adopted to identify the hPDLCs by detecting hPDLCs markers, including positive marker CD44 and CD90, and negative marker CD34 and CD45. Briefly, hPDLCs were incubated with antibodies against CD44 (1:100, ab157107, Abcam, Cambridge, UK), CD90 (1:100, ab23894, Abcam, Cambridge, UK), CD34 (1:100, ab78165, Abcam, Cambridge, UK) and CD45 (1:100, ab23910, Abcam, Cambridge, UK) for 30 min at 4 °C. The signals of CD44, CD90, CD34 and CD45 were detected by flow cytometry (FACSCalibur, BD, Franklin Lakes, NJ, USA), and analyzed by FlowJo software (Tree Star Inc., Ashland, OR, USA).

### hPDLCs transfection and treatment

hPDLCs in serum-free DMEM were plated into the 6-well plates with 1 × 10^5^ cells/mL per well. Mfn2 siRNA along with the corresponding NC (GeneChem, Shanghai, China) were individually transfected into hPDLCs by adopting Lipofectamine 3000 reagent (Thermo Fisher Scientific, San Jose, CA USA). The transfection dose of Mfn2 siRNA and siRNA NC was 50 nM [21]. hPDLCs were cultivated into DMEM with 10% FBS after transfection.

The construction of the PD cell model was achieved by inducing hPDLCs with 1 µg/mL Pg-LPS.

To examine cytotoxicity, Pue at final doses of 10, 20, 50, 100 and 200 µM were utilized to treat hPDLCs. To determine the optimal dose of Pue for treatment of the PD cell model, hPDLCs were treated with different doses of Pue (10, 20, 50, 100 and 200 µM) in the presence of Pg-LPS (1 µg/mL).

In the next efficacy study and mechanistic study of Pue, the following groups of hPDLCs were set: the Control group (hPDLCs cultivated in normal condition), the Pue group (hPDLCs only treated by 20 µM Pue), the LPS group (hPDLCs only induced by 1 µg/mL Pg-LPS), the LPS + Pue group (hPDLCs treated by 20 µM Pue in the presence of 1 µg/mL Pg-LPS), the LPS + Pue + CsA group (hPDLCs treated by 20 µM Pue and 1 µM CsA in the presence of 1 µg/mL Pg-LPS), the LPS + Pue + 3-MA group (hPDLCs treated by 20 µM Pue and 5 mM autophagy inhibitor 3-Methyladenine [3-MA; MCE, New Jersey, USA] in the presence of 1 µg/mL Pg-LPS), the LPS + Pue + siMfn2 NC group (hPDLCs transfected by Mfn2 siRNA NC, followed by 20 µM Pue treatment in the presence of 1 µg/mL Pg-LPS), and the LPS + Pue + siMfn2 group (hPDLCs transfected by Mfn2 siRNA, followed by 20 µM Pue treatment in the presence of 1 µg/mL Pg-LPS). hPDLCs of each group were cultivated under the relevant conditions at 37 °C, 5% CO_2_.

### Alizarin red staining and alkaline phosphatase (ALP) staining

hPDLCs were cultivated into the 6-well plates (5 × 10^3^ cells/mL per well), and then subjected to osteogenic differentiation induction by cultivated in 1 mL osteogenic induction medium (DMEM containing 10% FBS, 10 mM dexamethasone, 50 mg/L ascorbic acid and 10 mM β-glycerophosphate) at 37 °C, 5% CO_2_. The medium was refreshed every three days. After 14 days of osteogenic differentiation induction, hPDLCs were fixed by 4% paraformaldehyde for 20 min, and then stained with 2% alizarin red staining solution (Biolab Technology, Beijing, China) for 30 min at room temperature. The residual staining solution was washed out with phosphate buffered saline (PBS). hPDLCs were observed and photographed under a light microscope (Olympus, Tokyo, Japan). The alizarin red staining was qualified by detection the optical density (OD) value at 405 nm under a microplate reader (Biotek, Winooski, VT, USA).

For ALP staining, hPDLCs were stained for 20 min with ALP working solution (Biolab Technology, Beijing, China) after osteogenic differentiation induction and fixation. Following PBS washing, hPDLCs were photographed under a light microscope (Olympus, Tokyo, Japan). The ALP activity was determined by OD value that detected under a microplate reader (Biotek, Winooski, VT, USA) at a wave length of 520 nm.

### Oil O staining

hPDLCs in the 6-well plates (5 × 10^3^ cells/mL per well) were fostered in 1 mL DMEM containing 10% FBS, 10 µM insulin, 100 nM dexamethasone, 500 µM 3-isobutyl-1-methylxanthine and 200 µM indomycin for 14 days for the induction of adipogenic differentiation. The medium was changed every three days. After fixed by 4% polyformaldehyde, Oil Red O staining solution (Biolab Technology, Beijing, China) was added to stain hPDLCs for 30 min at room temperature. After washed by PBS, hPDLCs were placed under a light microscope (Olympus, Tokyo, Japan) for the observation of lipid droplets.

### Cell counting kit-8 (CCK-8) assay

After plated into the 96-well plates (5 × 10^3^ cells/mL per well), hPDLCs were cultivated for 24 and 48 h under the respective treatment conditions. At the two time points, hPDLCs were terminated from culture and treated by 10 µL CCK-8 solution (Biolab Technology, Beijing, China) for 2 h at 37 °C. The viability of hPDLCs was scrutinized by detecting OD value at 450 nm under a microplate reader (Biotek, Winooski, VT, USA).

### TMRE staining, mitosox red staining, and mito tracker staining

hPDLCs in the 6-well plates (1 × 10^5^ cells/mL per well) were fostered for 48 h under the respective treatment conditions. For the observation of mitochondrial membrane potential changes, N, N, N’, N’-tetra-methylethylenediamine (TMRE) was added to treat hPDLCs for 60 min at 37 °C. The excess dye was washed out by PBS. Flow cytometry analysis was implemented using a FACSCalibur system (Becton Dickinson, San Jose, CA, USA), and qualified by the CellQuest™ software system (Becton Dickinson, San Jose, CA, USA) to evaluate mitochondrial membrane potential.

Mitochondrial reactive oxygen species (ROS) level in hPDLCs was monitored by MitoSox Red staining. After the relevant treatment, hPDLCs were probed with 5 µM MitoSox Red staining solution (Yisheng Biotechnology, Shanghai, China) for 30 min at 37 °C in darkness. After washed with PBS, the fluorescence was detected using a FACSCalibur system (Becton Dickinson, San Jose, CA, USA), followed by being qualified by the CellQuest™ software system (Becton Dickinson, San Jose, CA, USA) to assess mitochondrial ROS level.

In Mito Tracker staining, hPDLCs were infected by GFP-LC3 lentivirus (GeneChem, Shanghai, China) for 5 h, and stained with Mito Tracker staining solution (Beyotime Biotechnology, Shanghai, China) for 30 min at 37 °C in the dark. The 4’, 6-diamidino-2-phenylindole (DAPI) solution (Beyotime Biotechnology, Shanghai, China) was adopted for nucleus staining. The GFP-LC3 fluorescence (green) and Mito Tracker fluorescence (red) was observed under a fluorescence microscope (Olympus, Tokyo, Japan). The LC3 dots as well as Mito aspect ratio were determined by the Image J software (ImageJ, NIH, Bethesda, MD, USA). The Mito aspect ratio was calculated by the length of the major axe/the length of the minor axe.

### mRFP-eGFP-LC3 assay

hPDLCs were transfected by mRFP-eGFP-LC3 plasmids (GeneChem, Shanghai, China) using Lipofectamine 3000 reagent. After transfection, hPDLCs were cultivated for 48 h under the relevant treatment conditions. After the termination of culture, hPDLCs were stained by DAPI solution for 5 min. The LC3 dots (green dots) were captured under a fluorescence microscope (Olympus, Tokyo, Japan), and qualified by the Image J software (ImageJ, NIH, Bethesda, MD, USA).

### Parkin/Tomm20 immunofluorescence staining

After 48 h of treatment under the relevant conditions, hPDLCs of each group were probed with rabbit anti-Parkin (1:100, ab315376, Abcam, Cambridge, UK) and mouse anti-TOMM20 (1:100, ab56783, Abcam, Cambridge, UK) primary antibodies overnight at 4 °C. Followed by this, Fluor488-conjugated goat anti-rabbit (1:200, ab150077, Abcam, Cambridge, UK) and Alexa Fluor594-conjugated goat anti-mouse (1:200, ab150116, Abcam, Cambridge, UK) secondary antibodies were added respectively for 2 h reaction at room temperature. DAPI was applied for nucleus staining. The co-location of Parkin and TOMM20 was observed under a fluorescence microscope (Olympus, Tokyo, Japan), and then qualified by the Image J software (ImageJ, NIH, Bethesda, MD, USA).

### ELISA

Periapical tissues of mice as well as hPDLCs (cultivated for 48 h under the relevant conditions) were gathered and lysed in lysis buffer. The lysate samples were centrifuged at 12,000 rpm for 10 min to collect the supernatant. Pro-inflammatory factors including tumor necrosis factor-α (TNF-α), interleukin-1β (IL‐1β) and interleukin-6 (IL-6) in the supernatant was detected using commercial TNF-α assay kit, IL-1β assay kit and IL-6 assay kit (Elabscience Biotechnology, Wuhan, China). The testing process was executed in strict accordance with the instructions of commercial kits.

### qRT-PCR

Periapical tissues from mice, along with hPDLCs treated for 48 h under the relevant conditions, were treated by TRIzol Reagent (Beyotime Biotechnology, Shanghai, China) to extract total RNA. A total of 1 µg total RNA sample was gathered for reverse transcription reaction by adopting a PrimeScript RT Reagent Kit (Takara, Tokyo, Japan). Afterwards, qRT-PCR was implemented on Agilent StrataGene Mx3005P Detection System (Santa Clara, CA, USA) via using a SYBR^®^Premix Ex TaqTM II (Takara, Tokyo, Japan). The reaction procedure was: 30 s at 95°C, and 40 cycles of 5 s at 95°C, 30 s at 60°C. The relative mRNA expression of genes was determined by the 2^−ΔΔCT^ method using glyceraldehyde-3-phosphate dehydrogenase (GAPDH) as the control. The primers were as below: Runt-related gene 2 (Runx2), 5’-CATGGCCGGGAATGATGAG-3’, 5’-TGTGAAGACCGTTATGGTCAAAGTG-3’. Osterix (OSX), 5’-CATCCATGCAGGCATCTCA-3’, 5’-CTGCCCACCACCTAACCAA-3’. Osteopontin (OPN), 5’-GACAGCAACGGGAAGACC-3’, 5’-CAGGCTGGCTTTGGAACT-3’. Osteocalcin (OCN), 5’-GAGGGCAGTAAGGTGGTGAA-3’, 5’-CGTCCTGGAAGCCAATGTG-3’. ALP, 5’-CATCGCCTATCAGCTAATGCACA-3’, 5’-ATGAGGTCCAGGCCATCCAG-3’. COL1A1, 5’-GACATGTTCAGCTTTGTGGACCTC-3’, 5’-AGGGACCCTTAGGCCATTGTGTA-3’. Optic atrophy 1 (OPA1), 5’-TTAGCCCTGAGACCATATCCT-3’, 5’-CAGCATCCACAGATCCATCT-3’. Fission 1 protein (Fis1), 5’-TGAACGAGCTGGTGTCTGT-3’, 5’-CGTATTCCTTGAGCCGGTA-3’. Mitofusin 1 (Mfn1), 5’-GGTCGCAAACTCTGAATCAA-3’, 5’-CAACTTTGAGCTCCTCCACC-3’. Mfn2, 5’-GCGAATTAAGCAGATTACGG-3’, 5’-ATGTCCTGCTGCATGGTCT-3’. Dynamin-related protein 1 (Drp1), 5’-AGATCCTGAAGATGTTGCCG-3’, 5’-CGCAATCTGATAGCTGTCCA-3’. GAPDH, 5’-GCCTTCCGTGTCCCCACTGC-3’, 5’-GGCTGGTGGTCCAGGGGTCT-3’.

### Western blot

The periapical tissues of mice and hPDLCs cultivated for 48 h under the relevant conditions were incubated with radio immunopreci pitation assay (RIPA) lysis buffer (Biolab Technology, Beijing, China) for 30 min on ice. The mixture was centrifuged for 15 min at 12,000 rpm and 4 °C. The supernatant that contained total proteins was gathered. For the separation of mitochondria in periapical tissues and hPDLCs, a Mitochondria Isolation Kit for Cell and Tissue (Biolab Technology, Beijing, China) was recruited based on the manufacturer’s directions. The harvested mitochondria were treated by RIPA lysis buffer for mitochondrial proteins extraction. A BCA Protein Assay Kit (Biolab Technology, Beijing, China) was adopted for the detection of the concentration of total proteins in the supernatant. The total protein sample with a weight of 30 µg was collected for protein separation by 10% sodium dodecyl sulfate-polyacrylamide gel electrophoresis, and thereafter transferred onto polyvinylidene fluoride (PVDF) membranes. The proteins loaded on the PVDF membranes were blocked in 5% skimmed milk for 1 h at room temperature. In sequence, the proteins were probed with primary antibodies (for 12 h at 4 °C) and then reacted with secondary antibody (for 2 h at room temperature). The protein signals were developed by treatment with enhanced chemiluminescent reagent (Beyotime Biotechnology, Shanghai, China), which were then qualified by the Image J software (ImageJ, NIH, Bethesda, MD, USA).

Primary antibodies were: rabbit anti-Light chain 3 (LC3) (1:1000, ab62721, Abcam, Cambridge, UK), anti-P62 (1:1000, ab91526, Abcam, Cambridge, UK), anti-Drp1 (1:1000, ab154879, Abcam, Cambridge, UK), anti-Phosphorylated-DRP1 (p-DRP1) (1:1000, 3455, Cell Signaling Technology, Danvers, MA, USA), anti-Mfn2 (1:1000, ab205236, Abcam, Cambridge, UK), anti-OPA1 (1:1000, ab42364, Abcam, Cambridge, UK), anti-Mfn1 (1:1000, PSI-7861, AmyJet Scientific, Wuhan, China), anti-Fis1 (1:1000, FNab03132, Fine Test Biotechnology, Wuhan, China), anti-Phosphatidylinositol 3-kinase (PI3K) (1:1000, orb137259, biorbyt, Wuhan, China), anti-Phosphorylated-PI3K (p-PI3K) (1:1000, AF5905, Beyotime Biotechnology, Shanghai, China), anti-Protein kinase B (AKT) (1:1000, ab8932, Abcam, Cambridge, UK), anti-Phosphorylated-AKT (p-AKT) (1:1000, YT638, Biolab Technology, Beijing, China), anti-Mammalian target of rapamycin (mTOR) (1:1000, ab1093, Abcam, Cambridge, UK), anti-Phosphorylated-mTOR (p-mTOR) (1:1000, K24032, Biolab Technology, Beijing, China), anti-Parkin (1:1000, ab315376, Abcam, Cambridge, UK), anti-GAPDH (1:1000, ab9485, Abcam, Cambridge, UK), and anti-Cytochrome C oxidase subunit IV (COXIV) (1:1000, ab202554, Abcam, Cambridge, UK). Secondary antibody was HRP conjugated goat anti-rabbit secondary antibody (1:3000, ab6721, Abcam, Cambridge, UK). GAPDH was utilized as the reference for total cellular proteins, while COXIV was reference for mitochondrial proteins.

### Statistical analysis

The statistical analysis and statistical graphing of quantitative data (shown as mean ± standard deviation) were conducted using GraphPad Prism 7 (GraphPad Software Inc., San Diego, CA, USA). Data were consistent with a normal distribution based on the Shapiro-Wilk test. One-way analyses of variance combined with Tukey’s post-hoc test were employed for the comparison of statistical difference in more than two groups. The statistical difference was considered statistically significant when *P* < 0.05.

## Results

### Pue treatment relieved periapical inflammation and bone destruction, and facilitated osteogenic differentiation and autophagy in periapical tissues of PD mice

Firstly, the in vivo safety of Pue on mice was assessed. As shown in Figure S1 A, after treated by 10 mg/kg Pue for 10 days, H&E staining was performed on heart, liver, spleen, lung, kinder, brain, skin and oral mucosa of mice. There was no obvious pathological damage to these above vital organs, skin and oral mucosa, suggesting that Pue had a favorable in vivo safety.

**Fig. 1:**
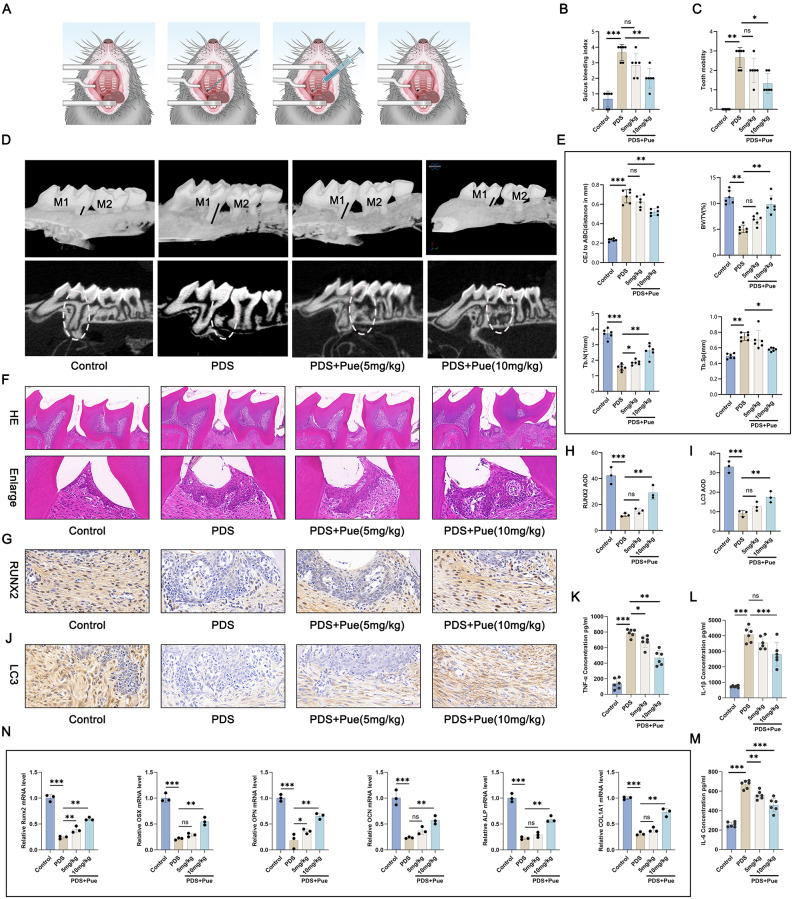
Pue treatment improved PD in mice. (**A**) Establishment of the mouse models of PD. (**B**-**C**) Sulcus bleeding index and tooth mobility analysis in mice. *n* = 6. (**D**-**E**) Micro-CT images and analysis of CEJ-ABC distance, BV/TV, Tb. N and Tb. Sp in mice. *n* = 6. (**F**) H&E staining of periapical tissues in mice was carried out to observe pathological changes. *n* = 3. (**G**-**J**) Immunohistochemistry of periapical tissues in mice was adopted to explore osteogenic differentiation capacity. *n* = 3. (**K**-**M**) Pro-inflammatory factors in periapical tissues of mice by ELISA. *N* = 6. (**N**) Expression of osteogenesis-related genes in periapical tissues of mice by qRT-PCR. *n* = 3. * *P* < 0.05. ** *P* < 0.01. *** *P* < 0.001. The “ns” symbol represented no obvious difference

This study constructed the mouse models of PD, and treated them with Pue at doses of 5 mg/kg and 10 mg/kg, respectively (Fig. 1A). The increased sulcus bleeding index and tooth mobility was monitored in PD mice (*P* < 0.01, *P* < 0.001), which was declined by 10 mg/kg Pue treatment (*P* < 0.05, *P* < 0.01) (Fig. 1B and C). Micro-CT analysis provided the evidence of the increased CEJ-ABC distance and Tb. Sp, along with the decreased BV/TV and Tb. N in PD mice (*P* < 0.01, *P* < 0.001). Intriguingly, 10 mg/kg Pue treatment reduced CEJ-ABC distance and Tb. Sp, and elevated BV/TV and Tb. N in PD mice (*P* < 0.05, *P* < 0.01). Indeed, 5 mg/kg Pue treatment only increased Tb. N in PD mice (*P* < 0.05), but had no obvious influence on the other three indicators (Fig. 1D and E).

H&E staining displayed severe periapical inflammation and bone destruction in PD mice, which was actually relieved by Pue treatment (Fig. 1F). By immunohistochemistry, a distinct down-regulation in osteogenic protein RUNX2 and autophagy protein LC3 was found in periapical tissues of PD mice (*P* < 0.001). In fact, 10 mg/kg Pue treatment prominently induced the expression of RUNX2 and LC3 proteins in periapical tissues of PD mice (*P* < 0.01) (Fig. 1G and J).

Pro-inflammatory factors in periapical tissues, including TNF-α, IL-1β and IL-6, were tested by ELISA. Excessive production of TNF-α, IL-1β and IL-6 was detected in periapical tissues of PD mice (*P* < 0.001). PD mice treated by 5 mg/kg Pue showed the reduced concentrations of TNF-α and IL-6 (*P* < 0.05, *P* < 0.01), where reduction in the three pro-inflammatory factors was monitored in those administered with 10 mg/kg Pue (*P* < 0.01, *P* < 0.001) (Fig. 1K and M). qRT-PCR of periapical tissues presented the reduced mRNA expression for Runx2, OSX, OPN, OCN, ALP and COL1A1 in PD mice (*P* < 0.001), whereas Pue treatment up-regulated the expression of these genes, in particular 10 mg/kg Pue treatment (*P* < 0.05, *P* < 0.01) (Fig. 1N). All of these evidence suggested that Pue treatment alleviated periapical inflammation and bone destruction and induced osteogenic differentiation and autophagy in PD.

### Pue treatment facilitated osteogenic differentiation capacity and repressed inflammatory response of the PD cell model

**Fig. 2:**
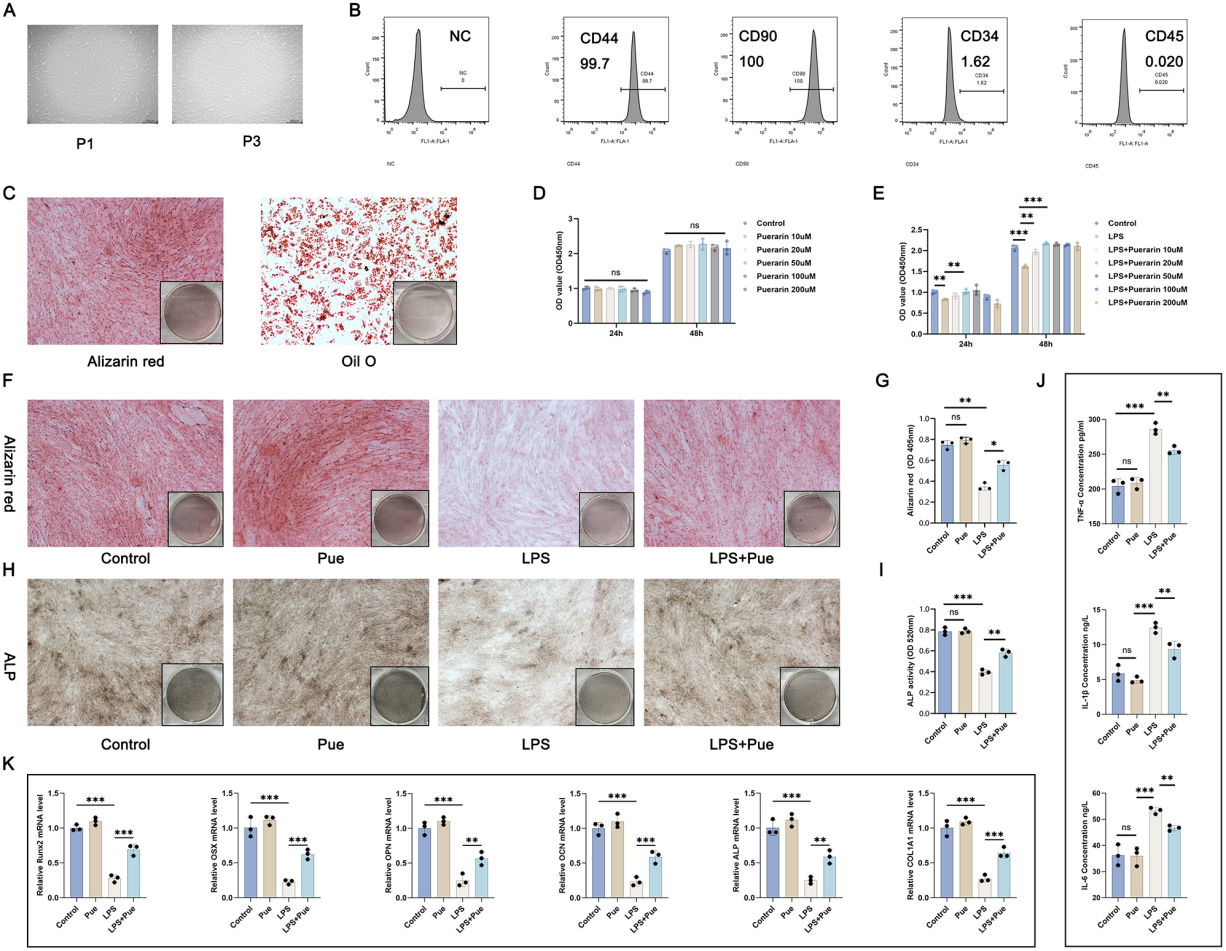
Pue treatment intensified osteogenic differentiation capacity and inhibited inflammatory response of the PD cell model (**A**) Passage culture of hPDLCs. (**B**) Identification of hPDLCs by flow cytometry. (**C**) Alizarin red staining and Oil O staining of hPDLCs to evaluate osteogenic and lipogenic differentiation capacities. (**D**) CCK-8 assay to study the cytotoxicity of Pue on hPDLCs. *n* = 3. (**E**) CCK-8 assay to identify the optimal dose of Pue on hPDLCs. *n* = 3. (**F**-**I**) Alizarin red staining and ALP staining were utilized to research the osteogenic differentiation ability of hPDLCs. *n* = 3. (**J**) ELISA of hPDLCs to detect pro-inflammatory factors. *n* = 3. (**K**) qRT-PCR of hPDLCs to assess the expression of osteogenesis-related genes. *n* = 3. * *P* < 0.05. ** *P* < 0.01. *** *P* < 0.001. The “ns” symbol represented no obvious difference

hPDLCs were passaged three times (Fig. 2A). Based on flow cytometry, the cultured hPDLCs were capable of expressing CD44 and CD90, but rarely expressing CD34 and CD45 (Fig. 2B). Alizarin red staining and Oil O staining showed favorable osteogenic and lipogenic differentiation capacities of hPDLCs (Fig. 2C).

According to data from CCK-8 assay, Pue at doses of 10, 20, 50, 100 and 200 µM were all non-toxic to hPDLCs (Fig. 2D). The cell model of PD was induced by Pg-LPS. As shown in Fig. 2E, Pg-LPS resulted in a marked decrease in hPDLCs viability (*P* < 0.01, *P* < 0.001). When the dose of Pue reached to 20 µM, a significantly enhanced viability of the Pg-LPS-induced hPDLCs was observed (*P* < 0.01, *P* < 0.001). Thus, in the follow-up study, Pue at a dose of 20 µM was selected to treat hPDLCs.

By Alizarin red staining and ALP staining, the osteogenic differentiation ability of hPDLCs was examined. Pue treatment alone had no obvious effect on the osteogenic differentiation ability of hPDLCs (the Pue group vs. the Control group), whereas Pg-LPS induction led to a much decrease in the osteogenic differentiation ability of hPDLCs (as evidenced by the attenuated Alizarin red staining and ALP activity) (the LPS group vs. the Control group) (*P* < 0.01, *P* < 0.001). Pue treatment intensified the osteogenic differentiation ability of the Pg-LPS-induced hPDLCs, as it increased Alizarin red staining and ALP activity (the LPS + Pue group vs. the LPS group) (*P* < 0.05, *P* < 0.01) (Fig. 2F and I).

In addition, Pue treatment alone did not cause inflammatory response in hPDLCs, because it had no obvious effect on the production of pro-inflammatory factors TNF-α, IL-1β and IL-6 (the Pue group vs. the Control group). However, Pg-LPS treatment dramatically elevated the levels of TNF-α, IL-1β and IL-6 in hPDLCs (the LPS group vs. the Control group) (*P* < 0.001). This effect of Pg-LPS was eliminated by Pue treatment (the LPS + Pue group vs. the LPS group) (*P* < 0.01) (Fig. 2J).

From results of qRT-PCR, Pue treatment alone did not influence the mRNA expression for Runx2, OSX, OPN, OCN, ALP and COL1A1 in hPDLCs (the Pue group vs. the Control group), whereas Pg-LPS induction remarkably decreased the expression of these genes (the LPS group vs. the Control group) (*P* < 0.001). In fact, the suppression of Pg-LPS on the mRNA expression for Runx2, OSX, OPN, OCN, ALP and COL1A1 in hPDLCs was abrogated by Pue treatment (the LPS + Pue group vs. the LPS group) (*P* < 0.01, *P* < 0.001) (Fig. 2K). Thus, these data was indicative that Pue treatment could enhance the osteogenic differentiation capacity and suppress inflammatory response of the PD cell model.

### Pue treatment enhanced mitochondrial autophagy in the PD cell model

**Fig. 3:**
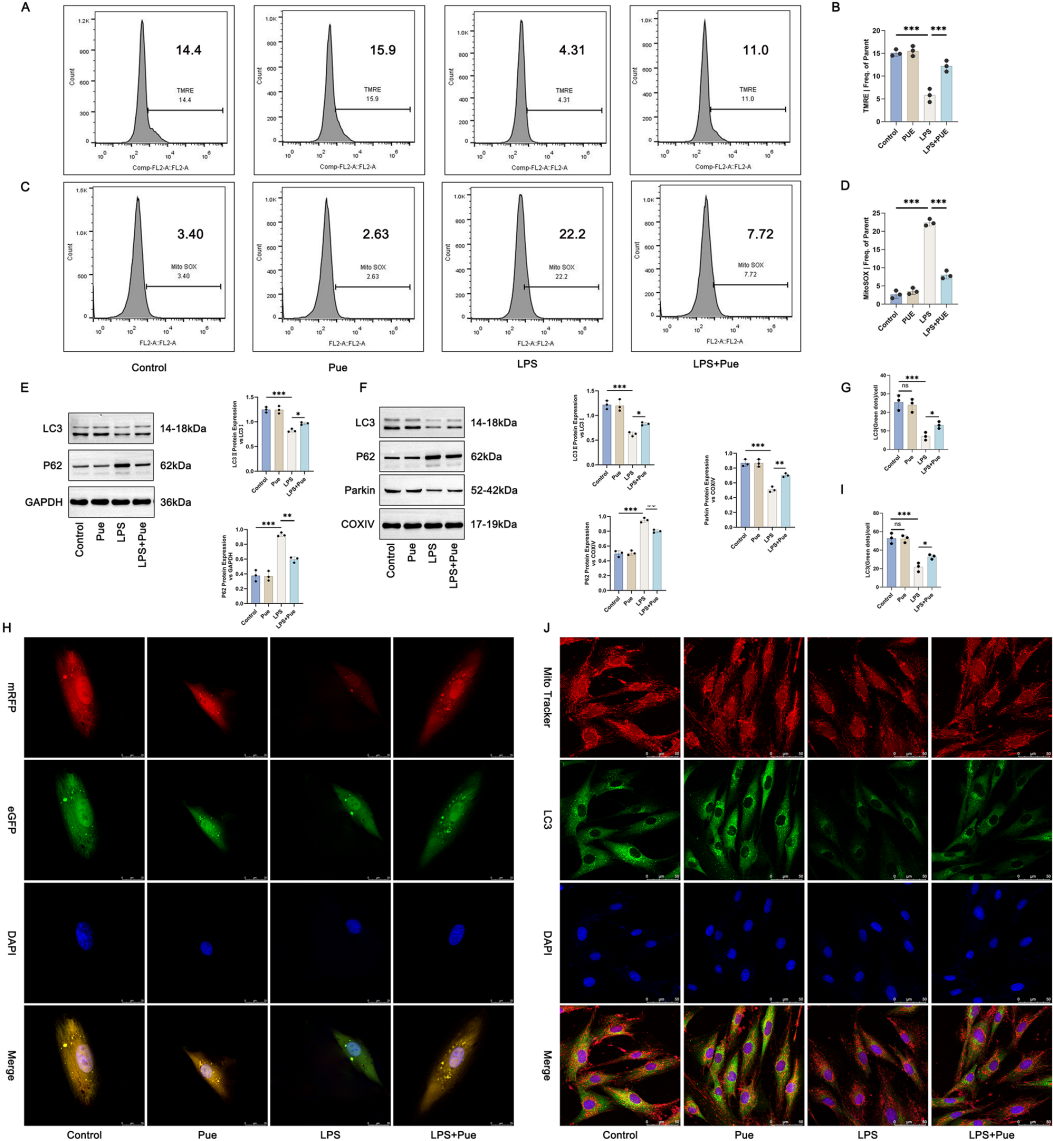
Pue treatment restored mitochondrial autophagy in the PD cell model. (**A**-**B**) TMRE staining combined with flow cytometry analysis was utilized to research mitochondrial membrane potential changes in hPDLCs. *n* = 3. (**C**-**D**) MitoSox Red staining combined with flow cytometry analysis was executed to monitor mitochondrial ROS level in hPDLCs. *n* = 3. (**E**-**F**) The expression of autophagy-related proteins in hPDLCs and mitochondria was detected by Western blot (Full-length blots/gels are presented in Supplementary Fig. 3E-LC3, Fig. 3E-P62, Fig. 3E-GAPDH, Fig. 3F-LC3, Fig. 3F-P62, Fig. 3F-Parkin, Fig. 3F-COXIV). *n* = 3. (**G**-**H**) LC3 dots in hPDLCs exhibited by mRFP-eGFP-LC3 assay. (**I**-**J**) LC3 dots in hPDLCs presented by Mito Tracker staining. *n* = 3. * *P* < 0.05. ** *P* < 0.01. *** *P* < 0.001. The “ns” symbol represented no obvious difference

By TMRE staining combined with flow cytometry analysis (Fig. 3A and B) and MitoSox Red staining combined with flow cytometry analysis (Fig. 3C and D), mitochondrial membrane potential changes and mitochondrial ROS level in hPDLCs of each group was investigated. Pue treatment alone did not affect mitochondrial membrane potential and mitochondrial ROS level in hPDLCs (the Pue group vs. the Control group). However, Pg-LPS treatment led to a much decrease in mitochondrial membrane potential and an increase in mitochondrial ROS level in hPDLCs (the LPS group vs. the Control group) (*P* < 0.001), which was abolished by Pue treatment (the LPS + Pue group vs. the LPS group) (*P* < 0.001).

The expression of autophagy-related proteins in hPDLCs of each group was scrutinized by Western blot. As a result, the Pg-LPS-induced hPDLCs expressed higher P62 protein but lower LC3II/LC3I protein (the LPS group vs. the Control group) (*P* < 0.001). Conversely, in the Pg-LPS-induced hPDLCs, Pue treatment reduced P62 protein, and elevated LC3II/LC3I protein (the LPS + Pue group vs. the LPS group) (*P* < 0.05, *P* < 0.01) (Fig. 3E). To research whether Pue regulated inflammation and osteogenic differentiation of the Pg-LPS-induced hPDLCs by modulating autophagy, Pue combined with autophagy inhibitor 3-MA was used to treat the Pg-LPS-induced hPDLCs. Based on ALP staining (Figure S1 B-C) and Alizarin red staining (Figure S1 D-E), the Pg-LPS-induced hPDLCs (the LPS group) showed a much reduction in ALP activity and Alizarin red staining than control hPDLCs (the Control group) (*P* < 0.0001). Pue treatment enhanced ALP activity and Alizarin red staining in the Pg-LPS-induced hPDLCs (the LPS + Pue group vs. the LPS group) (*P* < 0.0001), but this effect was reversed by 3-MA treatment (the LPS + Pue + 3-MA group vs. the LPS + Pue group) (*P* < 0.01, *P* < 0.001). qRT-PCR (Figure S1 F) and ELISA (Figure S1 G) were carried out to detect the expression of osteogenic differentiation-related factors (including OCN, OSX, Runx2, ALP, OPN and Collagen I) in hPDLCs and pro-inflammatory factors in culture medium (including IL-6, TNF-α and IL-1β). As a result, lower mRNA expression of OCN, OSX, Runx2, ALP, OPN and Collagen I, combined with higher levels of IL-6, TNF-α and IL-1β, was found in the Pg-LPS-induced hPDLCs (the LPS group vs. the Control group) (*P* < 0.001). Pue treatment increased mRNA expression of OCN, OSX, Runx2, ALP, OPN and Collagen I, and reduced levels of IL-6, TNF-α and IL-1β, comparatively (the LPS + Pue group vs. the LPS group) (*P* < 0.001), which was counteracted by 3-MA treatment (the LPS + Pue + 3-MA group vs. the LPS + Pue group) (*P* < 0.05, *P* < 0.01, *P* < 0.001). Thus, the promotion of Pue on osteogenic differentiation and suppression on inflammation in the Pg-LPS-induced hPDLCs was reversed by autophagy inhibitor 3-MA.

Furthermore, Pg-LPS treatment led to the reduction in mitochondrial LC3II/LC3I and Parkin proteins, but an elevation in mitochondrial P62 protein in hPDLCs (the LPS group vs. the Control group) (*P* < 0.001). This effect of Pg-LPS was counteracted by Pue treatment (the LPS + Pue group vs. the LPS group) (*P* < 0.05, *P* < 0.01) (Fig. 3F). Both of mRFP-eGFP-LC3 assay (Fig. 3G and H) and Mito Tracker staining (Fig. 3I and J) displayed the prominently reduced LC3 dots in hPDLCs after Pg-LPS induction (the LPS group vs. the Control group) (*P* < 0.001). Pue treatment increased LC3 dots in the Pg-LPS-induced hPDLCs (the LPS + Pue group vs. the LPS group) (*P* < 0.05). These results instructed that Pue treatment helped restore mitochondrial autophagy in the PD cell model.

### Pue treatment might relieve inflammation and induce osteogenic differentiation of the PD cell model by enhancing mitochondrial autophagy

**Fig. 4:**
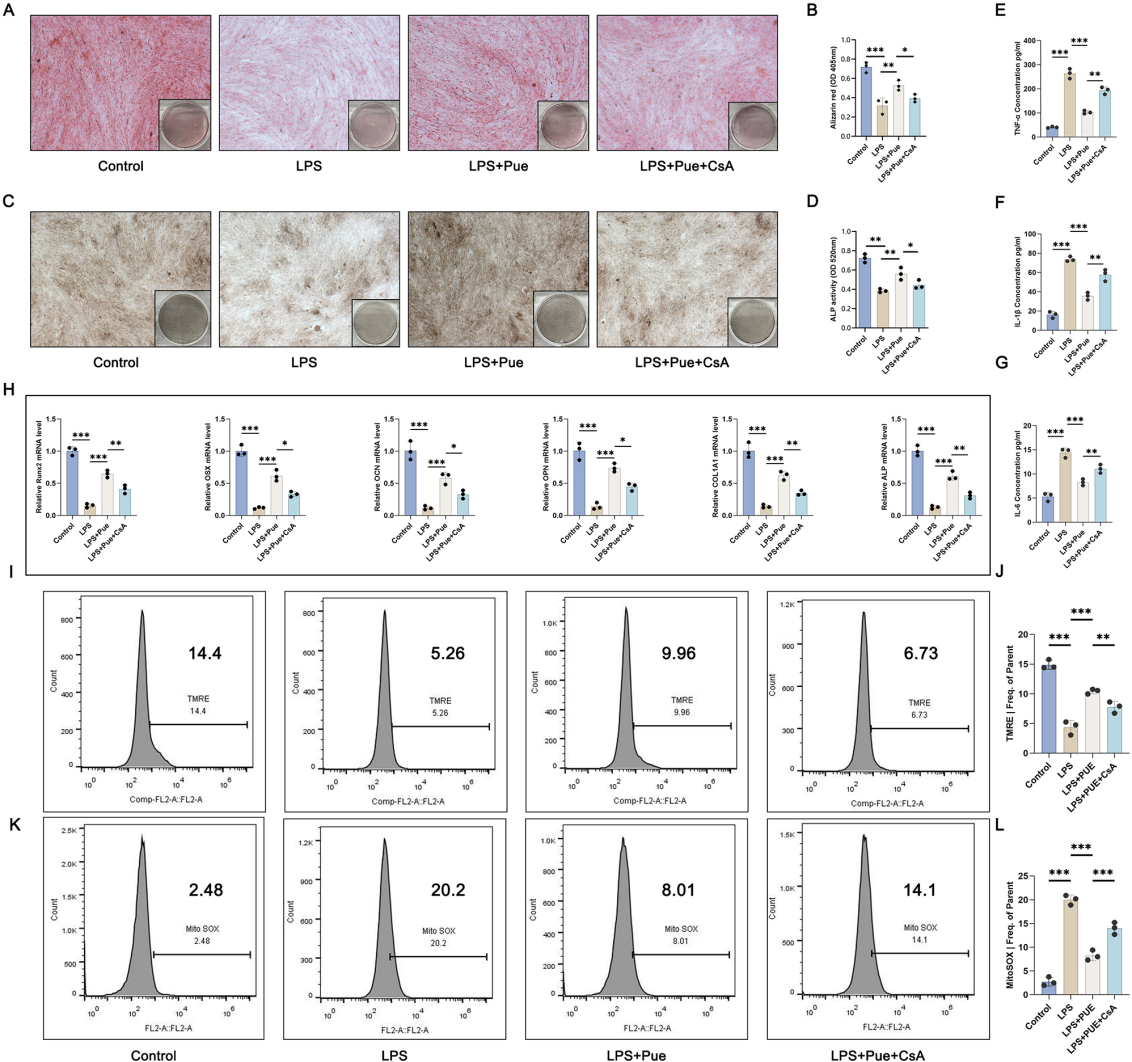
Pue promoted osteogenic differentiation but attenuated inflammation of the PD cell model through enhancing mitochondrial autophagy (**A**-**D**) Alizarin red staining along with ALP staining was adopted to detect the osteogenic differentiation ability of hPDLCs. *n* = 3. (**E**-**G**) ELISA was carried out to test pro-inflammatory factors in hPDLCs. *n* = 3. (**H**) Osteogenesis-related genes in hPDLCs were examined by qRT-PCR. *n* = 3. (**I**-**J**) TMRE staining combined with flow cytometry analysis was employed to monitor mitochondrial membrane potential changes in hPDLCs. *n* = 3. (**K**-**L**) Mitochondrial ROS level in hPDLCs was appraised by MitoSox Red staining combined with flow cytometry analysis. *n* = 3. * *P* < 0.05. ** *P* < 0.01. *** *P* < 0.001

CsA (an inhibitor of mitochondrial autophagy) was adopted to treat hPDLCs. Alizarin red staining (Fig. 4A and B) and ALP staining (Fig. 4C and D) were utilized to research the osteogenic differentiation capacity of hPDLCs. In hPDLCs, the attenuated Alizarin red staining and ALP activity that induced by Pg-LPS (the LPS group vs. the Control group) (*P* < 0.01, *P* < 0.001) treatment was reversed by Pue treatment (the LPS + Pue group vs. the LPS group) (*P* < 0.01). Intriguingly, the enhancement of Pue on Alizarin red staining and ALP activity was attenuated by CsA (the LPS + Pue + CsA group vs. the LPS + Pue group) (*P* < 0.05).

As presented by ELISA, the elevated TNF-α, IL-1β and IL-6 levels in the Pg-LPS-induced hPDLCs (the LPS group vs. the Control group) (*P* < 0.001) was declined by Pue treatment (the LPS + Pue group vs. the LPS group) (*P* < 0.001). However, the suppression of Pue on the production of TNF-α, IL-1β and IL-6 in the Pg-LPS-induced hPDLCs was reversed by CsA (the LPS + Pue + CsA group vs. the LPS + Pue group) (*P* < 0.01) (Fig. 4E and G).

qRT-PCR exhibited the reduced mRNA expression for Runx2, OSX, OPN, OCN, ALP and COL1A1 in the Pg-LPS-induced hPDLCs (the LPS group vs. the Control group) (*P* < 0.001), which was increased by Pue treatment (the LPS + Pue group vs. the LPS group) (*P* < 0.001). However, the promotion of Pue on mRNA expression for Runx2, OSX, OPN, OCN, ALP and COL1A1 in the Pg-LPS-induced hPDLCs was abolished by CsA (the LPS + Pue + CsA group vs. the LPS + Pue group) (*P* < 0.05, *P* < 0.01) (Fig. 4H).

By TMRE staining combined with flow cytometry analysis (Fig. 4I and J), Pg-LPS resulted in a decrease in mitochondrial membrane potential in hPDLCs (the LPS group vs. the Control group) (*P* < 0.001), but this effect of Pg-LPS was reversed by Pue treatment (the LPS + Pue group vs. the LPS group) (*P* < 0.001). In the Pg-LPS-induced hPDLCs, the increased mitochondrial membrane potential by Pue treatment was attenuated by CsA (the LPS + Pue + CsA group vs. the LPS + Pue group) (*P* < 0.01).

MitoSox Red staining combined with flow cytometry analysis (Fig. 4K and L) was performed to evaluate mitochondrial ROS level in hPDLCs. The exacerbated mitochondrial ROS by Pg-LPS induction (the LPS group vs. the Control group) (*P* < 0.001) was reduced by Pue treatment (the LPS + Pue group vs. the LPS group) (*P* < 0.001). The suppression effect of Pue on mitochondrial ROS in the Pg-LPS-induced hPDLCs was abrogated by CsA (the LPS + Pue + CsA group vs. the LPS + Pue group) (*P* < 0.001). Hence, all of the data indicated that Pue treatment might induce osteogenic differentiation and repress inflammation of the PD cell model via facilitating mitochondrial autophagy.

### Pue treatment maintained mitochondrial kinetic homeostasis in the PD cell model

**Fig. 5:**
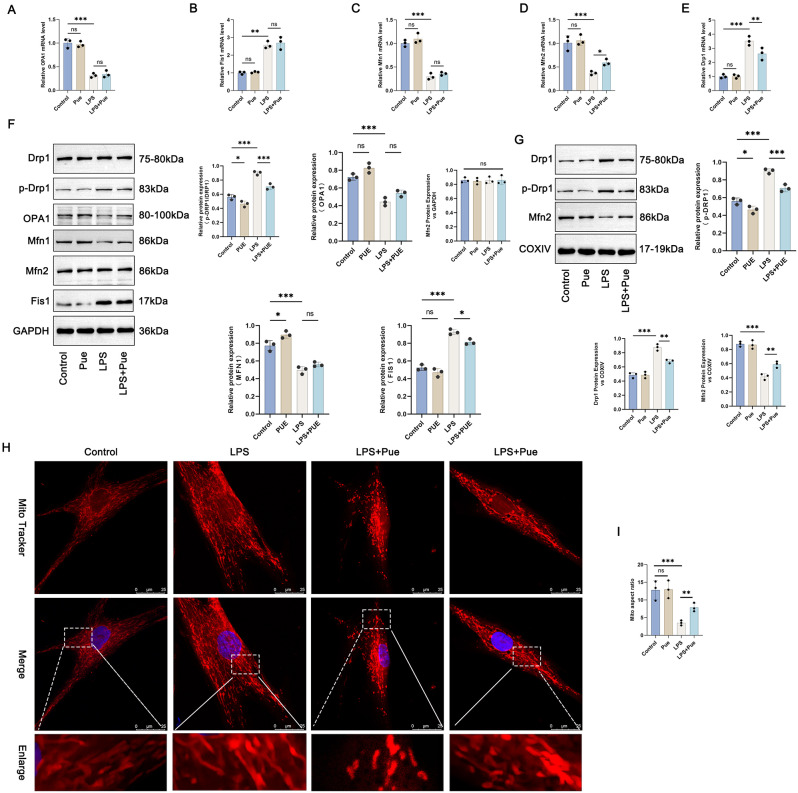
Pue treatment maintained mitochondrial kinetic homeostasis in the PD cell model. (**A**-**E**) Mitochondrial dynamics-related factors in hPDLCs were scrutinized by qRT-PCR. *n* = 3. (**F**-**G**) Mfn2, Drp1, p-DRP1, OPA1, Mfn1 and Fis1 proteins in cytoplasm and mitochondria of hPDLCs were assessed by Western blot, respectively (Full-length blots/gels are presented in Supplementary Fig. 5F-Drp1, Fig. 5F-Mfn2, Fig. 5F-GAPDH, Fig. 5G-Drp1, Fig. 5G-Mfn2, Fig. 5G-COXIV). *n* = 3. (**H**-**I**) Mito Tracker staining was employed to research mitochondria division in hPDLCs. *n* = 3. * *P* < 0.05. ** *P* < 0.01. *** *P* < 0.001. The “ns” symbol represented no obvious difference

Mitochondrial dynamics-related factors in hPDLCs were scrutinized by qRT-PCR. Pg-LPS induction led to a much diminish in the mRNA expression for OPA1, Fis1 and Mfn1 in hPDLCs (the LPS group vs. the Control group) (*P* < 0.01, *P* < 0.001), whereas Pue treatment showed no obvious influence on the expression of OPA1, Fis1 and Mfn1 mRNAs in the presence or absence of Pg-LPS (Fig. 5A and C). Intriguingly, Pue treatment alone had no obvious influence on the mRNA expression for Mfn2 and Drp1 in hPDLCs (the Pue group vs. the Control group). However, Pg-LPS induction resulted in a reduction in Mfn2 mRNA and an increase in Drp1 mRNA in hPDLCs (the LPS group vs. the Control group) (*P* < 0.001), but was counteracted by Pue treatment (the LPS + Pue group vs. the LPS group) (*P* < 0.05, *P* < 0.01) (Fig. 5D and E). Based on this discovery, this work further detected the expression of Mfn2, Drp1, p-DRP1, OPA1, Mfn1, and Fis1 proteins in cytoplasm of hPDLCs. Neither Pg-LPS nor Pue could affect the expression of Mfn2 protein in cytoplasm of hPDLCs. Pg-LPS treatment significantly reduced OPA1 and Mfn1 proteins, but increased p-DRP1/DRP1 and Fis1 proteins in cytoplasm of hPDLCs (the LPS group vs. the Control group) (*P* < 0.001). Pue had no obvious influence on the expression of OPA1 and Fis1 (the Pue group vs. the Control group). It decreased p-DRP1/DRP1 protein and increased Mfn1 protein in cytoplasm of hPDLCs (the Pue group vs. the Control group) (*P* < 0.05). In the Pg-LPS-induced hPDLCs, Pue treatment did not affect the expression of OPA1 and Mfn1 proteins, but diminished the expression of p-DRP1/DRP1 and Fis1 proteins in cytoplasm (the LPS + Pue group vs. the LPS group) (*P* < 0.05, *P* < 0.001) (Fig. 5F). The expression of Drp1, p-DRP1 and Mfn2 proteins in mitochondria of hPDLCs was further examined. hPDLCs treated by Pg-LPS showed lower Mfn2 protein but higher Drp1 and p-DRP1 proteins in mitochondria of hPDLCs (the LPS group vs. the Control group) (*P* < 0.001). Pue treatment declined p-DRP1 protein in mitochondria of hPDLCs (the Pue group vs. the Control group) (*P* < 0.05), but had no obvious effect on mitochondrial Drp1 and Mfn2 proteins. As referred to the LPS group, hPDLCs of the LPS + Pue group showed lower Drp1 and p-DRP1 proteins but higher Mfn2 protein in mitochondria (*P* < 0.01, *P* < 0.001) (Fig. 5G).

Mito Tracker staining was utilized to research mitochondria division. Pue treatment alone showed no statistically significant effect on mitochondria aspect ratio (the Pue group vs. the Control group). However, Pg-LPS treatment led to a much decline in mitochondria aspect ratio in hPDLCs (the LPS group vs. the Control group) (*P* < 0.001). Indeed, the suppression of Pg-LPS on mitochondria aspect ratio was reversed by Pue treatment (the LPS + Pue group vs. the LPS group) (*P* < 0.01) (Fig. 5H and I). Thereby, these above data illustrated that Pue treatment was effective in maintaining mitochondrial kinetic homeostasis.

### Pue treatment might intensify mitochondrial autophagy in the PD cell model by activating Mfn2

**Fig. 6:**
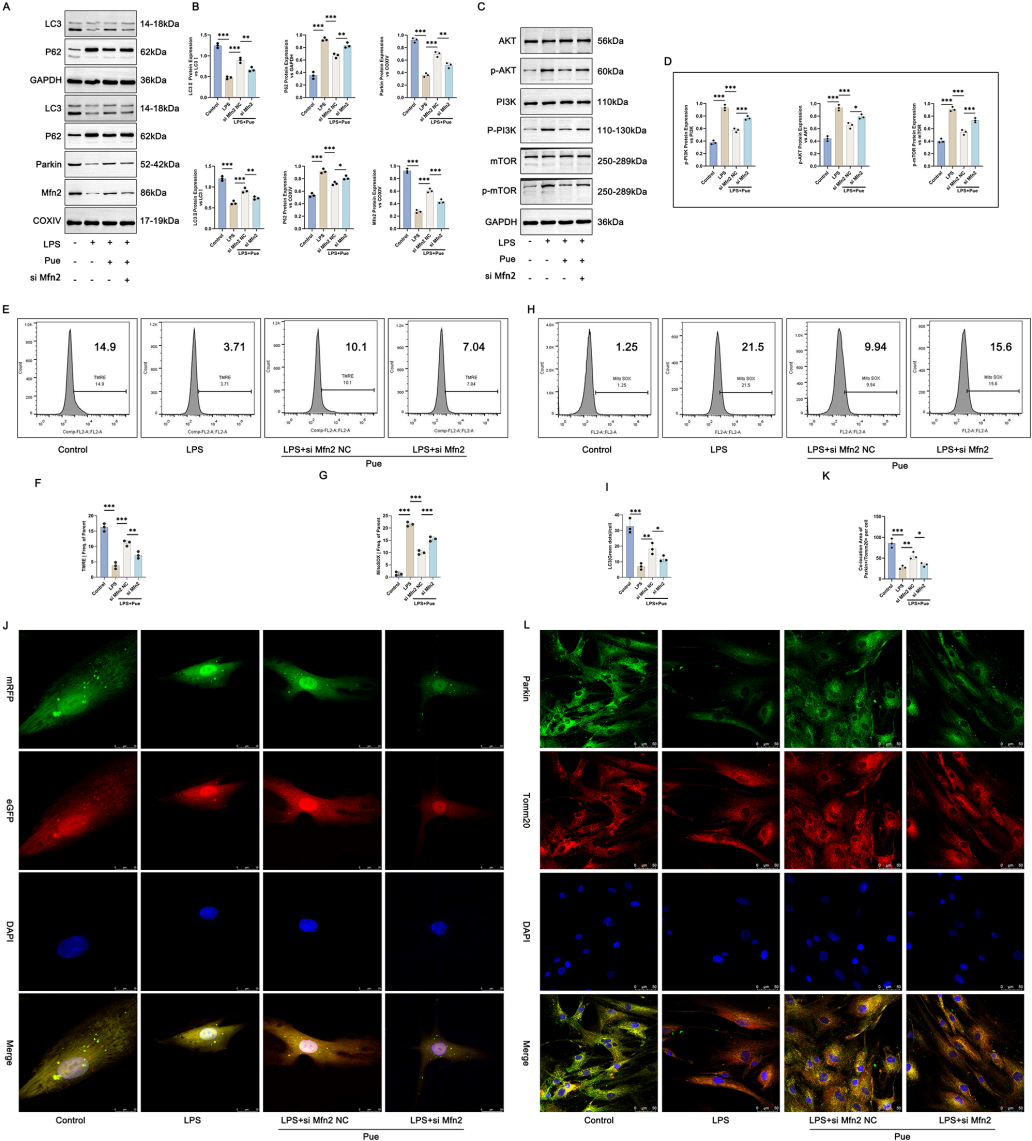
Pue treatment enhanced mitochondrial autophagy in the PD cell model via promoting Mfn2 expression. (**A**-**B**) Western blot was executed to monitor the expression of autophagy-related proteins in hPDLCs and mitochondria (Full-length blots/gels are presented in Supplementary Fig. 6A-LC3, Fig. 6A-P62, Fig. 6A-GAPDH, Fig. 6A-Mitochondrial LC3, Fig. 6A-Mitochondrial P62, Fig. 6A-Mitochondrial Parkin, Fig. 6A-Mitochondrial Mfn2, Fig. 6A-COXIV). *n* = 3. (**C**-**D**) The PI3K/AKT/mTOR pathway activity in hPDLCs was scrutinized by Western blot (Full-length blots/gels are presented in Supplementary Fig. 6C-AKT, Fig. 6C-p-AKT, Fig. 6C-PI3K, Fig. 6C-p-PI3K, Fig. 6C-mTOR, Fig. 6C-p-mTOR, Fig. 6C-GAPDH). *n* = 3. (**E**-**F**) TMRE staining combined with flow cytometry analysis was performed for the observation of mitochondrial membrane potential changes in hPDLCs. *n* = 3. (**G**-**H**) MitoSox Red staining combined with flow cytometry analysis was employed to detect mitochondrial ROS level in hPDLCs. *n* = 3. (**I**-**J**) mRFP-eGFP-LC3 assay was adopted to explore LC3 dots in hPDLCs. *n* = 3. (**K**-**L**) Parkin/Tomm20 immunofluorescence staining of hPDLCs was executed to evaluate mitochondrial autophagy. *n* = 3. * *P* < 0.05. ** *P* < 0.01. *** *P* < 0.001

Mfn2 siRNA was transfected into hPDLCs, in order to verify whether Pue induced mitochondrial autophagy in the Pg-LPS-induced hPDLCs by activating Mfn2. Western blot was adopted to scrutinize the expression of autophagy-related proteins in hPDLCs and mitochondria. Pg-LPS treatment decreased LC3II/LC3I protein and increased P62 protein in hPDLCs (the LPS group vs. the Control group) (*P* < 0.001). Conversely, Pue treatment led to an increase in LC3II/LC3I protein and a decrease in P62 protein in the Pg-LPS-induced hPDLCs (the LPS + Pue + siMfn2 NC group vs. the LPS group) (*P* < 0.001). In fact, as matched to the LPS + Pue + siMfn2 NC group, hPDLCs of the LPS + Pue + siMfn2 group expressed lower LC3II/LC3I protein but higher P62 protein (*P* < 0.01). On the other hand, Pg-LPS treatment led to the reduced mitochondrial Parkin, LC3II/LC3I and Mfn2 protein expression as well as the increased mitochondrial P62 protein expression in hPDLCs (the LPS group vs. the Control group) (*P* < 0.001), which was counteracted by Pue treatment (the LPS + Pue + siMfn2 NC group vs. the LPS group) (*P* < 0.001). However, the promotion of Pue on mitochondrial Parkin, LC3II/LC3I and Mfn2 proteins and its suppression on mitochondrial P62 protein was abolished by Mfn2 silencing (the LPS + Pue + siMfn2 group vs. the LPS + Pue + siMfn2 NC group) (*P* < 0.05, *P* < 0.01, *P* < 0.001) (Fig. 6A and B).

The PI3K/AKT/mTOR pathway activity in hPDLCs was appraised by Western blot. As a result, Pg-LPS induction abnormally activated the expression of p-PI3K/PI3K, p-AKT/AKT and p-mTOR/mTOR proteins in hPDLCs (the LPS group vs. the Control group) (*P* < 0.001), but this influence of Pg-LPS was eliminated by Pue treatment (the LPS + Pue + siMfn2 NC group vs. the LPS group) (*P* < 0.001). The suppression of Pue on the activity of the PI3K/AKT/mTOR pathway was attenuated by the loss of Mfn2 (the LPS + Pue + siMfn2 group vs. the LPS + Pue + siMfn2 NC group) (*P* < 0.05, *P* < 0.001) (Fig. 6C and D).

Additionally, TMRE staining combined with flow cytometry analysis (Fig. 6E and F), MitoSox Red staining combined with flow cytometry analysis (Fig. 6G and H), mRFP-eGFP-LC3 assay (Fig. 6I and J), and Parkin/Tomm20 immunofluorescence staining (Fig. 6K and L) were utilized to monitor mitochondrial membrane potential changes, mitochondrial ROS level, LC3 dots and mitochondrial Parkin expression in hPDLCs, respectively. The decreased mitochondrial membrane potential, increased mitochondrial ROS level, reduced LC3 dots and Parkin/Tomm20 co-location induced by Pg-LPS (the LPS group vs. the Control group) (*P* < 0.001) in hPDLCs was abrogated by Pue treatment (the LPS + Pue + siMfn2 NC group vs. the LPS group) (*P* < 0.01, *P* < 0.001). Mfn2 knockdown abolished the elevation of Pue on mitochondrial membrane potential, LC3 dots and Parkin/Tomm20 co-location, and its suppression on mitochondrial ROS level in the Pg-LPS-induced hPDLCs (the LPS + Pue + siMfn2 group vs. the LPS + Pue + siMfn2 NC group) (*P* < 0.05, *P* < 0.01, *P* < 0.001). Based on these results, Pue was suggested to enhance mitochondrial autophagy in the PD cell model via activating Mfn2 expression.

### Pue treatment might attenuate periapical inflammation and bone destruction, and facilitate osteogenic differentiation in periapical tissues of PD mice by facilitating mitochondrial autophagy via activating mitochondrial Mfn2

**Fig. 7:**
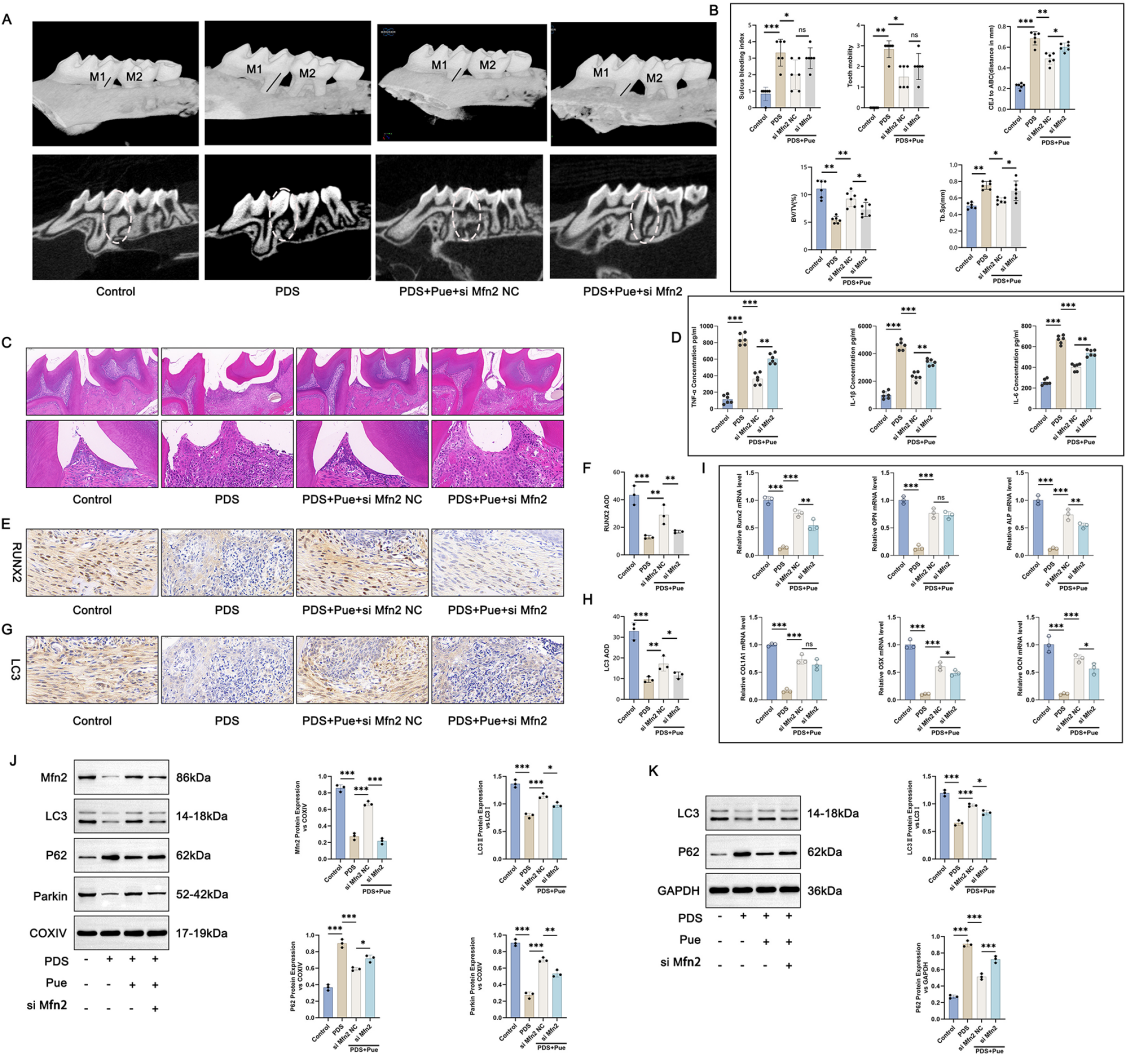
Pue treatment alleviated periapical inflammation, bone destruction, and induced osteogenic differentiation in periapical tissues of PD mice by promoting mitochondrial autophagy via activating mitochondrial Mfn2. (**A**-**B**) Micro-CT images and analysis of sulcus bleeding index, tooth mobility, CEJ-ABC distance, BV/TV and Tb. Sp of mice was implemented. *n* = 6. (**C**) H&E staining of periapical tissues in mice was carried out to observe pathological changes. *n* = 3. (**D**) ELISA of mice periapical tissues was utilized to evaluate pro-inflammatory factors. *n* = 6. (**E**-**H**) Immunohistochemistry of mice periapical tissues was utilized for the detection of RUNX2 and LC3 protein expression. *n* = 3. (**I**) qRT-PCR of mice periapical tissues was used to appraise the expression of osteogenesis-related genes. *n* = 3. (**J**) Western blot was executed to monitor mitochondrial autophagy-related proteins in periapical tissues of mice (Full-length blots/gels are presented in Supplementary Fig. 7J-Mfn2, Fig. 7J-LC3, Fig. 7J-P62, Fig. 7J-Parkin, Fig. 7J-COXIV). *n* = 3. (**K**) The expression of autophagy-related proteins in periapical tissues of mice was assessed by Western blot (Full-length blots/gels are presented in Supplementary Fig. 7K-LC3, Fig. 7K-P62, Fig. 7K-GAPDH). *n* = 3. * *P* < 0.05. ** *P* < 0.01. *** *P* < 0.001. The “ns” symbol represented no obvious difference

Adenovirus-based Mfn2 siRNA was transfected into periodontal tissues of PD mice, and mice were treated with Pue at a dose of 10 mg/kg. As shown in Fig. 7A and B, by Micro-CT analysis, the abnormally increased sulcus bleeding index, tooth mobility, CEJ-ABC distance and Tb. Sp, as well as the decreased BV/TV in PD mice in PD mice (the PD group vs. the Control group) (*P* < 0.01, *P* < 0.001) was reversed by Pue treatment (the PD + Pue + siMfn2 NC group vs. the PD group) (*P* < 0.05, *P* < 0.01). When relative to mice in the PD + Pue + siMfn2 NC group, those in the PD + Pue + siMfn2 group possessed higher CEJ-ABC distance and Tb. Sp, as well as lower BV/TV (*P* < 0.05).

According to H&E staining, severe periapical inflammation and bone destruction in PD mice (the PD group vs. the Control group) was alleviated by Pue treatment (the PD + Pue + siMfn2 NC group vs. the PD group). However, Mfn2 silencing reversed the relief of Pue on periapical inflammation and bone destruction in PD mice (the PD + Pue + siMfn2 group vs. the PD + Pue + siMfn2 NC group) (Fig. 7C). ELISA of periapical tissues displayed higher TNF-α, IL-1β and IL-6 levels in PD mice (the PD group vs. the Control group) (*P* < 0.001), which was reduced by Pue treatment (the PD + Pue + siMfn2 NC group vs. the PD group) (*P* < 0.001). In fact, the suppression of Pue on TNF-α, IL-1β and IL-6 production in periapical tissues of PD mice was counteracted by the silencing of Mfn2 (the PD + Pue + siMfn2 group vs. the PD + Pue + siMfn2 NC group) (*P* < 0.01) (Fig. 7D).

By immunohistochemistry, the reduced RUNX2 and LC3 proteins in periapical tissues of PD mice (the PD group vs. the Control group) (*P* < 0.001) was up-regulated by Pue treatment (the PD + Pue + siMfn2 NC group vs. the PD group) (*P* < 0.01). In contrast, the enhancement of Pue on RUNX2 and LC3 protein expression in periapical tissues of PD mice was attenuated by Mfn2 silencing (the PD + Pue + siMfn2 group vs. the PD + Pue + siMfn2 NC group) (*P* < 0.05, *P* < 0.01) (Fig. 7E and H). Based on results from qRT-PCR of periapical tissues, PD mice expressed less mRNAs for Runx2, OSX, OPN, OCN, ALP and COL1A1 genes (the PD group vs. the Control group) (*P* < 0.001), while Pue treatment intensified the expression of these genes (the PD + Pue + siMfn2 NC group vs. the PD group) (*P* < 0.001). The enhancement of Pue on the expression of Runx2, OSX, OCN, and ALP mRNAs in periapical tissues of PD mice was reversed by Mfn2 deletion (the PD + Pue + siMfn2 group vs. the PD + Pue + siMfn2 NC group) (*P* < 0.05, *P* < 0.01) (Fig. 7I).

Western blot was utilized for the expression of autophagy-related proteins in periapical tissues of PD mice. As a result, PD mice expressed much lower mitochondrial Mfn2, LC3II/LC3I and Parkin proteins, as well as higher mitochondrial P62 protein in periapical tissues (the PD group vs. the Control group) (*P* < 0.001). After Pue treatment, the increased mitochondrial Mfn2, LC3II/LC3I and Parkin proteins, as well as the reduced mitochondrial P62 protein occurred in periapical tissues of PD mice (the PD + Pue + siMfn2 NC group vs. the PD group) (*P* < 0.001). However, the promotion of Pue on mitochondrial Mfn2, LC3II/LC3I and Parkin proteins, and its suppression on mitochondrial P62 protein, was reversed by Mfn2 silencing (the PD + Pue + siMfn2 group vs. the PD + Pue + siMfn2 NC group) (*P* < 0.05, *P* < 0.01, *P* < 0.001) (Fig. 7J). Simultaneously, the reduced LC3II/LC3I protein and increased P62 protein in periapical tissues of PD mice (the PD group vs. the Control group) (*P* < 0.001) was abrogated by Pue treatment (the PD + Pue + siMfn2 NC group vs. the PD group) (*P* < 0.001). Conversely, in periapical tissues of PD mice, the elevation of Pue on LC3II/LC3I protein and its suppression on P62 protein were eliminated by Mfn2 silencing (the PD + Pue + siMfn2 group vs. the PD + Pue + siMfn2 NC group) (*P* < 0.05, *P* < 0.001) (Fig. 7K). Taken together, Pue treatment might relieve PD, such as alleviating periapical inflammation and bone destruction as well as enhancing osteogenic differentiation in periapical tissues, by enhancing mitochondrial autophagy via activating mitochondrial Mfn2.

## Discussion

The initiating factor of PD is oral bacterial infection, which can eventually lead to loosening and loss of teeth if left untreated [22]. This study highlighted the therapeutic effect of Pue on PD mice, as it decreased CEJ-ABC distance and Tb. Sp, increased Tb. N and BV/TV, reduced sulcus bleeding index and tooth mobility, suppressed inflammation and bone destruction, and promoted osteogenic differentiation. One of the main clinical features of PD is the inflammatory response, and the overproduction of pro-inflammatory factors (such as TNF-α, IL-1β and IL-6) in the periodontal tissues is the primary cause of damage to gingival tissues and ligaments [23]. By means of pathological staining and ELISA of periapical tissues, this paper revealed the mitigating effect of Pue on periapical inflammation, bone destruction and its suppression on the production of TNF-α, IL-1β and IL-6 in PD mice. Similarly, in the Pg-LPS-induced hPDLCs, the inhibition of Pue on the production of TNF-α, IL-1β and IL-6 was demonstrated. hPDLCs are ideal candidates for the regenerative repair of periodontal tissues and bone regeneration in PD, as it possesses multidirectional differentiation potential and elf-renewal capacity [22]. This study instructed the enhancement of Pue on the expression of Runx2, OSX, OPN, OCN, ALP and COL1A1 in periapical tissues of PD mice and the Pg-LPS-induced hPDLCs. As we know, Runx2, OSX, OPN, OCN, ALP and COL1A1 are osteogenic markers, of which, Runx2, ALP and COL1A1 are early-stage osteogenic markers and OSX, OPN and OCN are late-stage osteogenic markers [24]. Thus, Pue was capable of inducing osteogenic differentiation of hPDLCs in PD. Limited data have suggested the therapeutic potential of Pue on PD [11, 12]. The findings in this work were consistent with the previous studies. The focus of this work was on the therapeutic effects and molecular mechanisms of Pue on PD, thus the interaction form between Pue and hPDLCs was not revealed in detail. Previous studies have reported that Pue can exert its pharmacological effects by interacting with membrane proteins as well as by being taken up and transported by cells [25, 26]. Therefore, in this study, Pue might also regulate the biological behavior of hPDLCs through these approaches.

Mitochondrial autophagy is a form of mitochondrial self-protection, which maintains the stability of quantity and structure of mitochondria and exerts a crucial role in maintaining normal mitochondrial function [13]. It has been revealed that abnormal mitochondrial autophagy can affect the biological behaviors of hPDLCs [13, 27]. According to the present study, in periapical tissues of PD mice, the repressed autophagy (as shown by the reduced LC3 protein expression) was found, whereas Pue treatment increased the expression of LC3 protein. Through in vitro study, the suppressed autophagy (as implied by the reduced LC3II/LC3I and the increased P62 proteins), the impaired mitochondrial function (as evidenced by the declined mitochondrial membrane potential and the intensified mitochondrial ROS level), as well as the blocked mitochondrial autophagy (as displayed by the reduced mitochondrial LC3II/LC3I, Parkin, and the elevated mitochondrial P62 proteins) was found in the Pg-LPS-induced hPDLCs. LC3II/LC3I and mitochondrial Parkin are autophagy-inducing proteins, while P62 accumulates when autophagy is blocked [28, 29]. Intriguingly, the impaired mitochondrial function and suppressed mitochondrial autophagy in the Pg-LPS-induced hPDLCs was eliminated by Pue treatment. Rescue experiment by co-treatment of the Pg-LPS-induced hPDLCs with Pue and CsA indicated that, Pue might promote osteogenic differentiation, attenuate inflammation, and maintain normal mitochondrial function by activating mitochondrial autophagy. In several other disease studies, Pue treatment has been implied to alleviate diseases by alleviating mitochondrial dysfunction and promoting mitochondrial autophagy [15, 30, 31]. The novelty of this paper was that, Pue was suggested for the first time to promote osteogenic differentiation and suppress inflammation of hPDLCs in PD by activating mitochondrial autophagy.

Mitochondrial dynamics plays an essential role in normal cellular physiological activities. The abnormality of mitochondrial dynamics can lead to a variety of diseases, and is usually accompanied by the disrupted mitochondrial autophagy [32]. OPA1, Fis1, Mfn1, Mfn2, Drp1 and p-DRP1 are mitochondrial dynamics-related factors involved in the regulation of mitochondrial fusion and fission processes [16, 33]. Drp1 and Fis1 are key proteins participating in mitochondrial fission, and Drp1 phosphorylation regulates mitochondrial fission by promoting the translocation from cytoplasm to mitochondria [16, 33]. Mfn1, Mfn2 and OPA1 are involved in mediating mitochondrial membrane fusion [33]. In this paper, Pue was discovered to have no obvious influence on the expression of Drp1, Mfn2, OPA1, and Fis1, whereas it intensified Mfn1 expression and suppressed p-Drp1 expression in the Pg-LPS-induced hPDLCs. Further research suggested that Pue could increase mitochondrial Mfn2 expression and reduce mitochondrial Drp1 and p-Drp1 expression in the Pg-LPS-induced hPDLCs, but rather than affecting cellular Mfn2 and Drp1 expression. Mfn2 has been suggested to be contributed to the restoration of mitochondrial autophagy, and Mfn2 combined with Drp1 are involved in the maintenance of normal mitochondrial function by mediating the mitochondrial fusion and fission processes [34, 35]. The activation of mitochondrial autophagy has been researched to promote osteogenic differentiation of hPDLCs [13]. Mitochondrial Mfn2 is an essential component for initiating downstream signals of mitochondrial autophagy; the reduction in mitochondrial Mfn2 prevents the initiation of mitochondrial autophagy, which leads to mitochondrial damage and oxidative stress injury [36]. Oxidative stress injury is a key factor in the progression of PD; in PD, Mfn2 promotes periodontal regeneration, attenuates oxidative stress injury, inflammation, and apoptosis of periodontal cells, maintains mitochondrial integrity, and facilitates mitochondrial fusion; these functions of Mfn2 in PD offers a broad prospect for its treatment [37]. However, whether Mfn2 mitigates PD by activating mitochondrial autophagy remains poorly explained currently. In this research, by in vitro study of the Pg-LPS-induced hPDLCs, Pue treatment increased mitochondrial Mfn2, and Mfn2 silencing reversed the mitochondrial autophagy enhancement, mitochondrial function maintenance induced by Pue treatment. In this work, hPDLCs were transfected by 50 nM Mfn2 siRNA, and this safety dose was referred to a previous study [21]. Thus, Pue was suggested to relieve PD progression by activating mitochondrial autophagy via enhancing mitochondrial Mfn2.

Based on this study, in the PD cell model, the PI3K/AKT/mTOR pathway was over activated, whereas Pue treatment suppressed the activity of it. In inflammation-related diseases, the blockage of the PI3K/AKT/mTOR pathway activity has been shown to activate mitochondrial autophagy to alleviate inflammatory response [38, 39]. Some anti-inflammatory components have been reported to exert their therapeutic effects on PD by suppressing the activation of the PI3K/AKT/mTOR pathway, while the activator of the PI3K/AKT/mTOR pathway eliminated the anti-inflammatory effect of these anti-inflammatory components [40]. The suppression of Pue on the PI3K/AKT/mTOR pathway activity has been reported previously [41]. This article firstly demonstrated that Pue relieved PD by inactivating the PI3K/AKT/mTOR pathway. More interestingly, Mfn2 silencing abrogated the inhibition of Pue on the PI3K/AKT/mTOR pathway activity and the promotion on mitochondrial autophagy in the PD cell model. Mfn2 has been found to induce autophagy by inactivating the PI3K/AKT/mTOR pathway [42]. Therefore, Mfn2 could suppress the activity of the PI3K/AKT/mTOR pathway to activate mitochondrial autophagy in PD in this study, and Pue could facilitate mitochondrial autophagy in PD by inducing Mfn2 expression. By in vivo experiment, adenovirus-based Mfn2 siRNA with a viral titer of 10^9^ VG/µL and an injection volume of 500 nL was transfected into mice, and this transfection dose had been shown to be safety by a previous study [20]. As a result, the relief on PD (such as the alleviated periapical inflammation and bone destruction), induction on osteogenic differentiation, and promotion on mitochondrial autophagy induced by Pue treatment was counteracted by Mfn2 silencing. This further indicated that Pue might exert a therapeutic effect on PD by intensifying mitochondrial autophagy via activating mitochondrial Mfn2 expression. Collectively, the current study provided sufficient basis and exact molecular mechanism for the application of Pue in the treatment of PD.

There are limitations in this study. Firstly, it would be very interesting to study the long-term effects of Pue for PD. Second, this study performed Parkin/Tomm20 immunofluorescence staining to observe mitochondrial autophagy. It would be better to verify mitochondrial autophagy by transmission electron microscope. Third, performing experiments to investigate the tissue distribution and metabolism of Pue after local injection into the mouse gingiva would be useful. Finally, whether activation of the PI3K/AKT/mTOR pathway regulates Mfn2 or Drp1 translocation from cytosol to mitochondria should be explored. However, due to the limitations of our laboratory, these issues cannot be performed at present, which will be the focus of our future research.

## Conclusion

Pue was suggested to have therapeutic effect on PD. In PD mice, Pue treatment reduced sulcus bleeding index and tooth mobility; increased Tb. N and BV/TV; diminished CEJ-ABC distance and Tb. Sp; relieved periapical inflammation and bone destruction; and facilitated osteogenic differentiation. In vitro mechanistic study showed that, Pue might relieve inflammation and enhance osteogenic differentiation of the PD cell model by intensifying mitochondrial autophagy via up-regulating the expression of mitochondrial Mfn2. This mechanism was further validated by in vivo rescue experiments in PD mice. Thus, Pue treatment was proposed to be an effective strategy for treating PD in the clinical setting. In clinical practice, Pue may be useful for treating PD.

## Electronic supplementary material

Below is the link to the electronic supplementary material.

Supplementary Material 1: Fig. S1 In vivo safety testing of Pue, and autophagy inhibitor 3-MA treatment of the PD cell model. (A) In vivo safety testing of Pue. By H&E staining, Pue was non-toxic to heart, liver, spleen, lung, kinder, brain, skin and oral mucosa of mice. (B-E) ALP staining and Alizarin red staining indicated that autophagy inhibitor 3-MA reversed the promotion of Pue on osteogenic differentiation of the Pg-LPS-induced hPDLCs. *n* = 3. (F) By qRT-PCR, autophagy inhibitor 3-MA abolished the promotion of Pue on the expression of osteogenic differentiation factors in the Pg-LPS-induced hPDLCs. *n* = 3. (G) Based on ELISA, the suppression of Pue on the levels of pro-inflammatory factors (including IL-6, TNF-α and IL-1β) in the Pg-LPS-induced hPDLCs was counteracted by autophagy inhibitor 3-MA. *n* = 3. * *P* < 0.05. ** *P* < 0.01. *** *P* < 0.001. **** *P* < 0.0001

Supplementary Material 2

## Data Availability

The datasets used and/or analysed during the current study are available from the corresponding author on reasonable request.

## Abbreviations

Pue: Puerarin
PD: Periodontitis
Pg-LPS: Gingivalis-lipopolysaccharide
Mfn2: Mitofusin 2
hPDLCs: Human periodontal ligament cells
NC: Negative control
Micro-CT: Micro-computed tomography
CEJ: Cementoenamel junction
ABC: Alveolar bone crest
BV/TV: Bone volume fraction
Tb. N: Trabecular bone number
Tb.Sp: Trabecular spacing
H&E: Hematoxylin-eosin
EDTA: Ethylene-diamine tetra-acetic acid
HRP: Horseradish peroxidase
DMEM: Dulbecco’s modified Eagle’s medium
FBS: Fetal bovine serum
ALP: Alkaline phosphatase
PBS: Phosphate buffered saline
OD: Optical density
ROS: Reactive oxygen species
DAPI: 4’, 6-diamidino-2-phenylindole
qRT-PCR: Quantitative reverse transcription-polymerase chain reaction
GAPDH: Glyceraldehyde-3-phosphate dehydrogenase
Runx2: Runt-related gene 2
OSX: Osterix
OPN: Osteopontin
OCN: Osteocalcin
COL1A: Collagen type I alpha 1 chai
OPA1: Optic atrophy 1
Fis1: Fission 1 protein
Mfn1: Mitofusin 1
Drp1: Dynamin-related protein 1
RIPA: Radio immunopreci pitation assay
PVDF: Polyvinylidene fluoride
LC3: Light chain
PI3K: Phosphatidylinositol 3-kinas
p-PI3: Phosphorylated-PI3K
AKT: Protein kinase
p-AKT: Phosphorylated-AKT
mTOR: Mammalian target of rapamycin
p-mTOR: Phosphorylated-mTO
COXIV: Cytochrome C oxidase subunit IV
