# Supplementary figures and images for: Puerarin relives inflammation, bone destruction and facilitates osteogenic differentiation in periodontitis by enhancing mitochondrial autophagy via activating mitochondrial Mitofusin 2
Source: Stem Cell Res Ther. 2025 May 1;16:218. doi: 10.1186/s13287-025-04355-w (PMC12044717; doi:10.1186/s13287-025-04355-w)

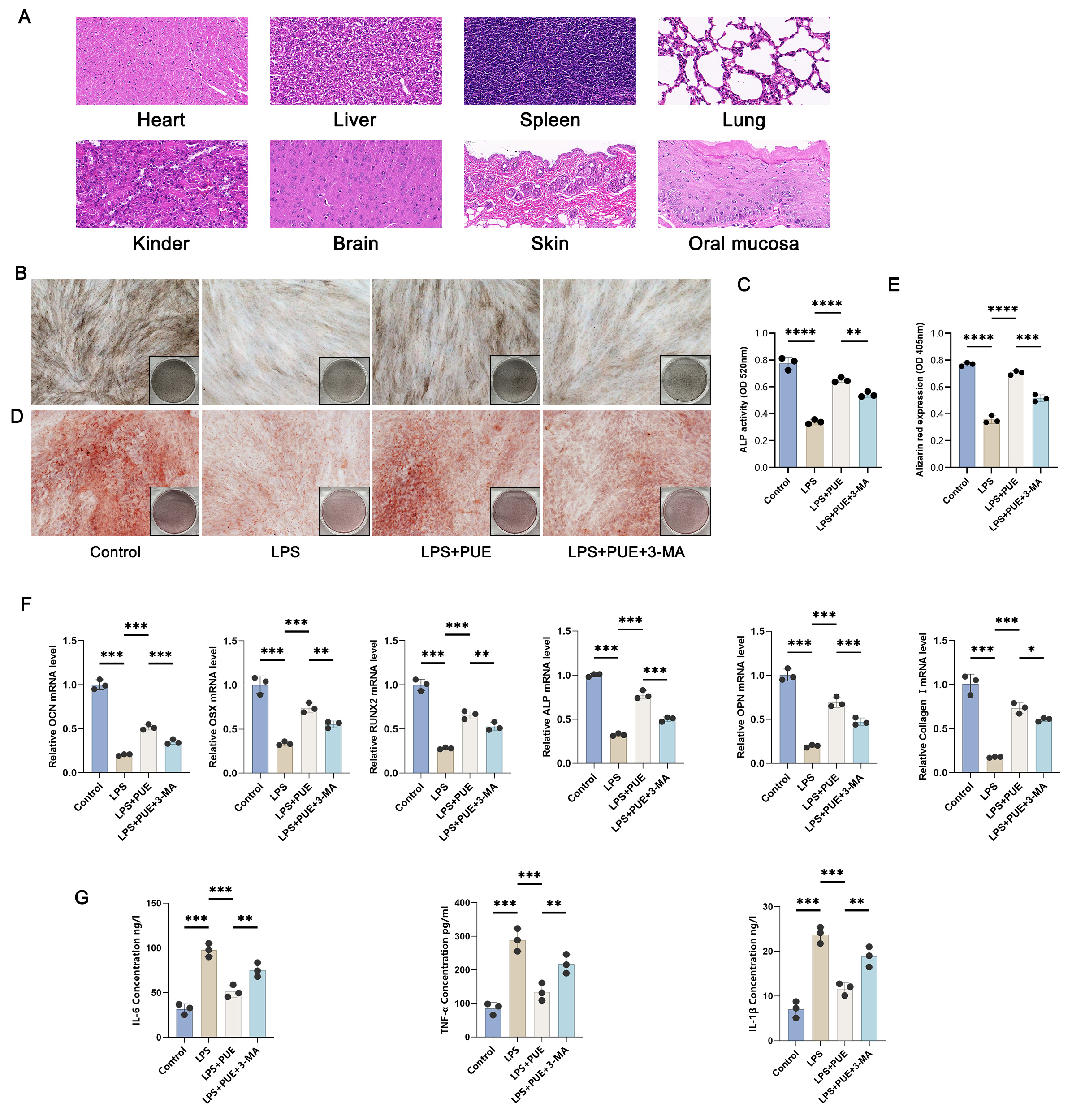

Supplement: Supplementary file 1 — Supplementary Material 1: Fig. S1 In vivo safety testing of Pue, and autophagy inhibitor 3-MA treatment of the PD cell model. (A) In vivo safety testing of Pue. By H&E staining, Pue was non-toxic to heart, liver, spleen, lung, kinder, brain, skin and oral mucosa of mice. (B-E) ALP staining and Alizarin red staining indicated that autophagy inhibitor 3-MA reversed the promotion of Pue on osteogenic differentiation of the Pg-LPS-induced hPDLCs. n = 3. (F) By qRT-PCR, autophagy inhibitor 3-MA abolished the promotion of Pue on the expression of osteogenic differentiation factors in the Pg-LPS-induced hPDLCs. n = 3. (G) Based on ELISA, the suppression of Pue on the levels of pro-inflammatory factors (including IL-6, TNF-α and IL-1β) in the Pg-LPS-induced hPDLCs was counteracted by autophagy inhibitor 3-MA. n = 3. * P < 0.05. ** P < 0.01. *** P < 0.001. **** P < 0.0001 [file 13287_2025_4355_MOESM1_ESM.jpg]
