# Supplementary material for: Puerarin relives inflammation, bone destruction and facilitates osteogenic differentiation in periodontitis by enhancing mitochondrial autophagy via activating mitochondrial Mitofusin 2
Source: Stem Cell Res Ther. 2025 May 1;16:218. doi: 10.1186/s13287-025-04355-w (PMC12044717; doi:10.1186/s13287-025-04355-w)
Supplement: Supplementary file 2 — Supplementary Material 2 [file 13287_2025_4355_MOESM2_ESM.ppt]

## Slide 1
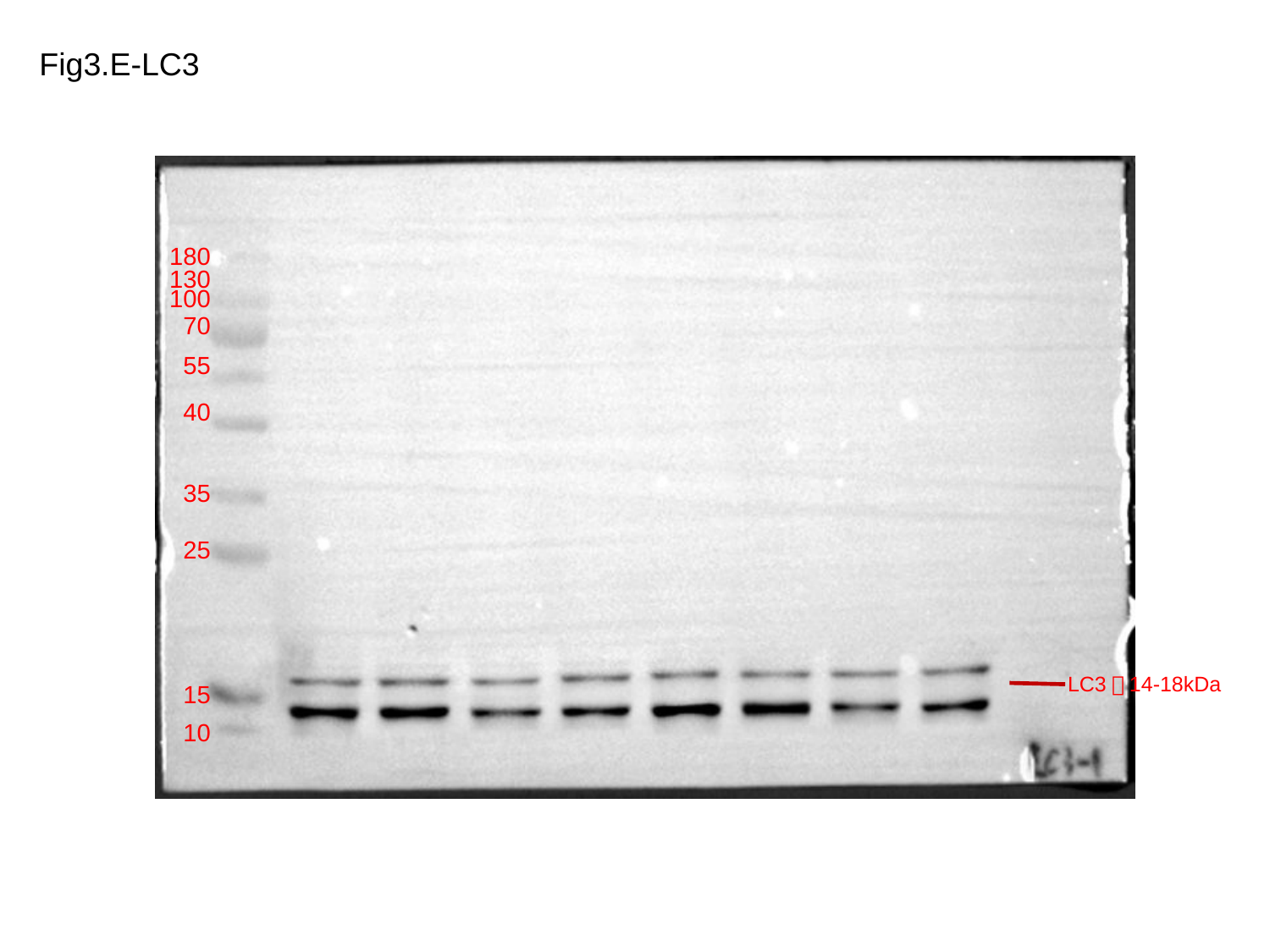

Fig3.E-LC3
180
130
100
70
55
40
35
25
LC3：14-18kDa
15
10

## Slide 2
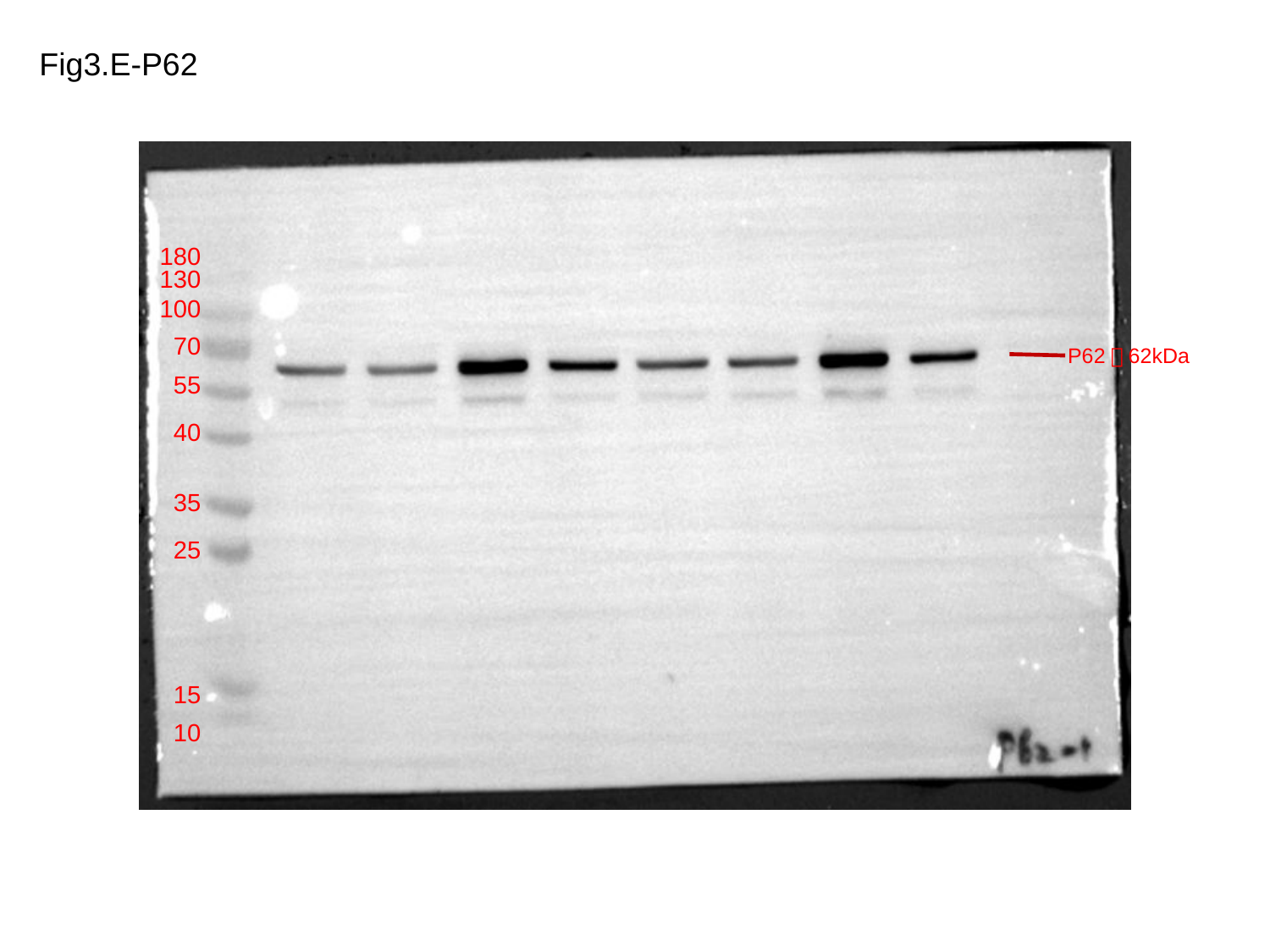

Fig3.E-P62
180
130
100
70
P62：62kDa
55
40
35
25
15
10

## Slide 3
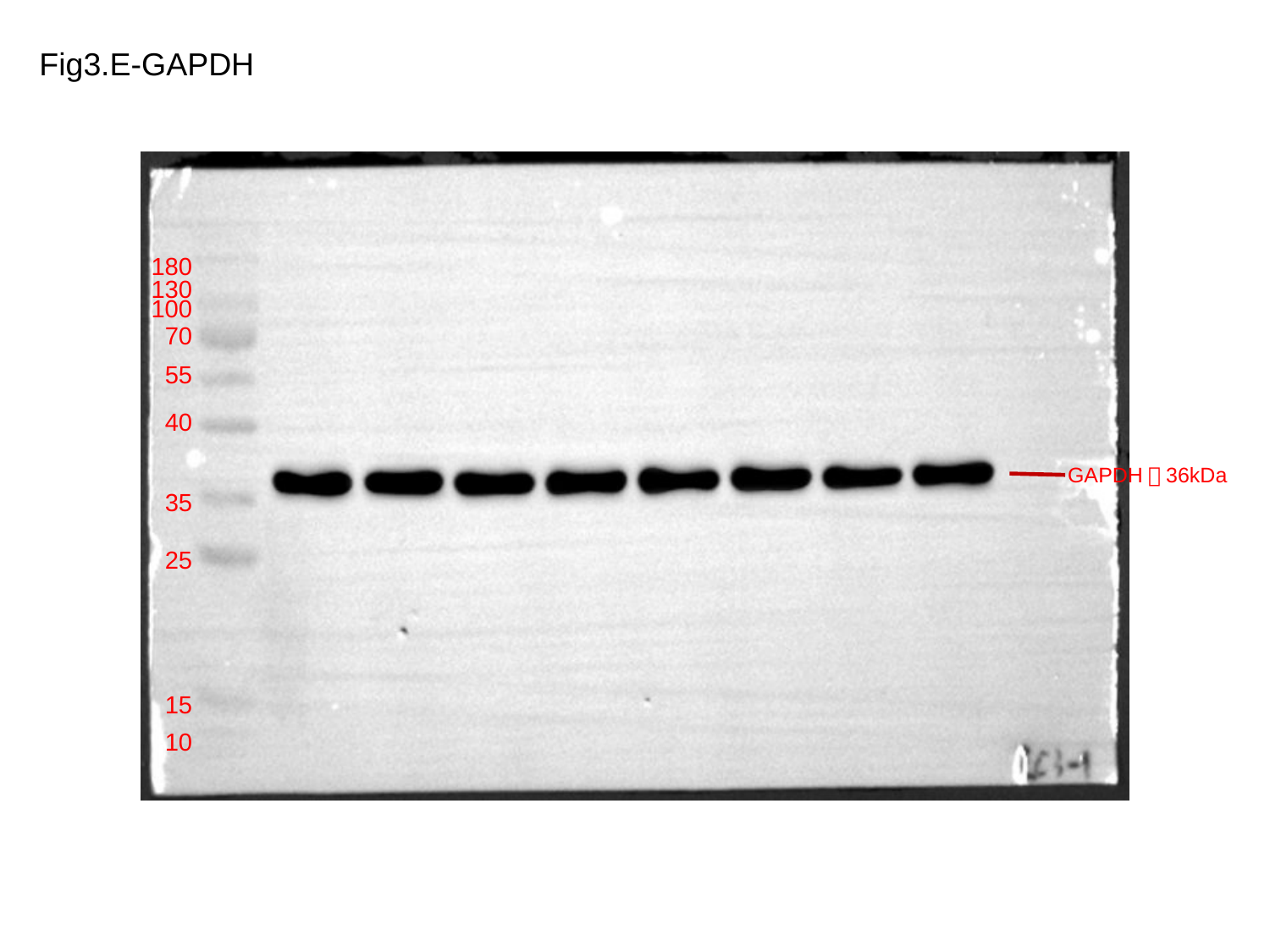

Fig3.E-GAPDH
180
130
100
70
55
40
GAPDH：36kDa
35
25
15
10

## Slide 4
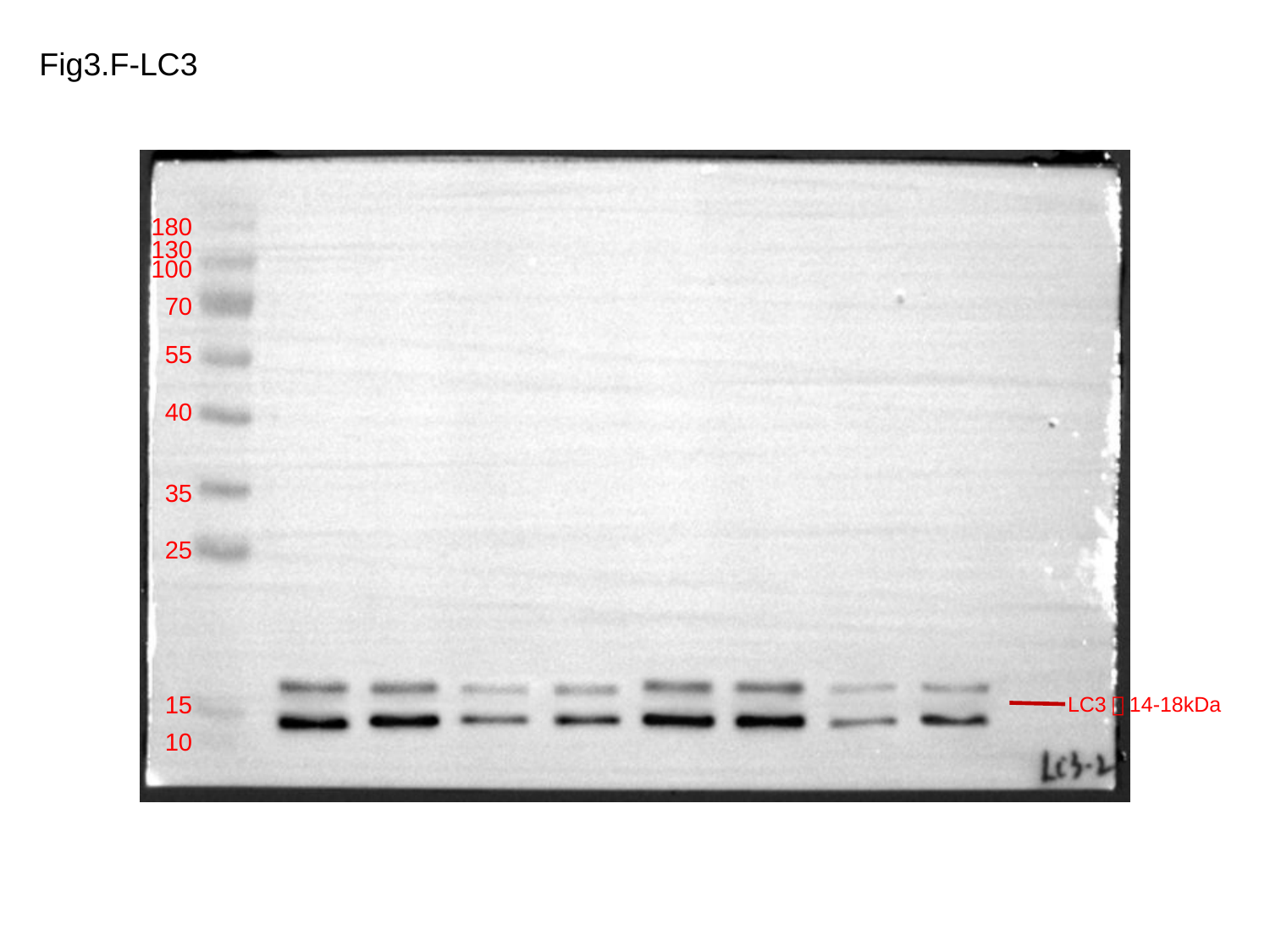

Fig3.F-LC3
180
130
100
70
55
40
35
25
15
LC3：14-18kDa
10

## Slide 5
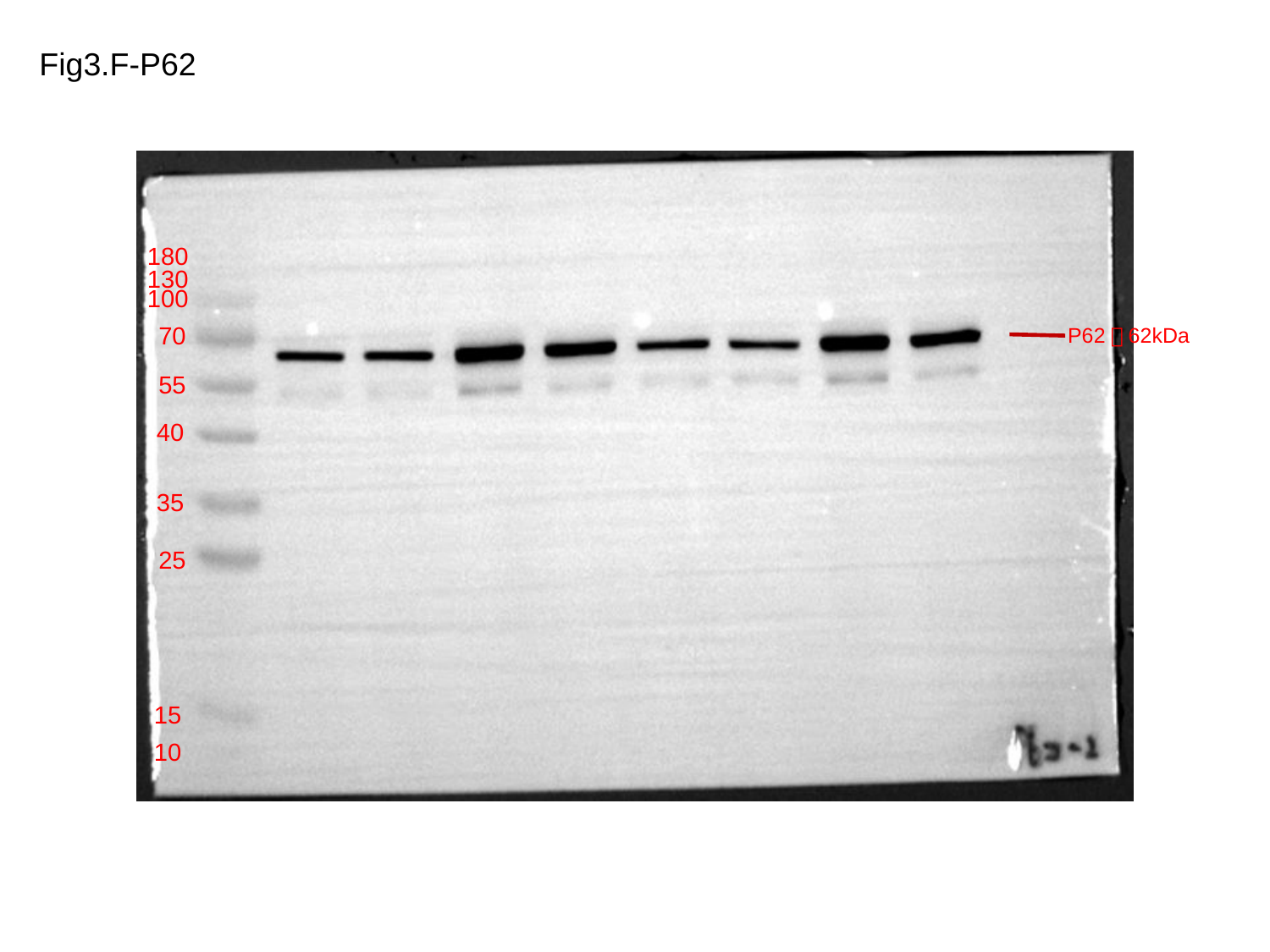

Fig3.F-P62
180
130
100
70
P62：62kDa
55
40
35
25
15
10

## Slide 6
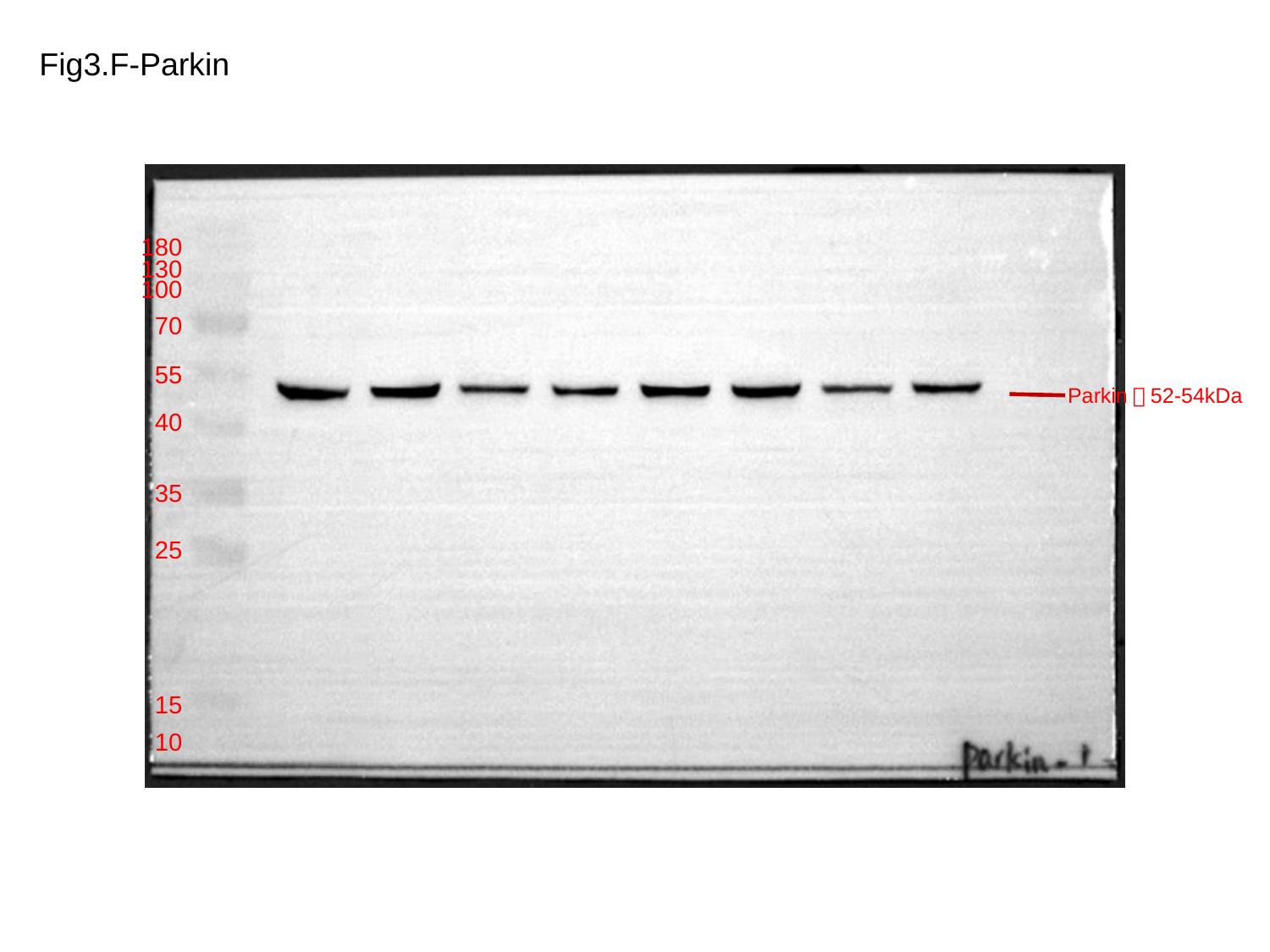

Fig3.F-Parkin
180
130
100
70
55
Parkin：52-54kDa
40
35
25
15
10

## Slide 7
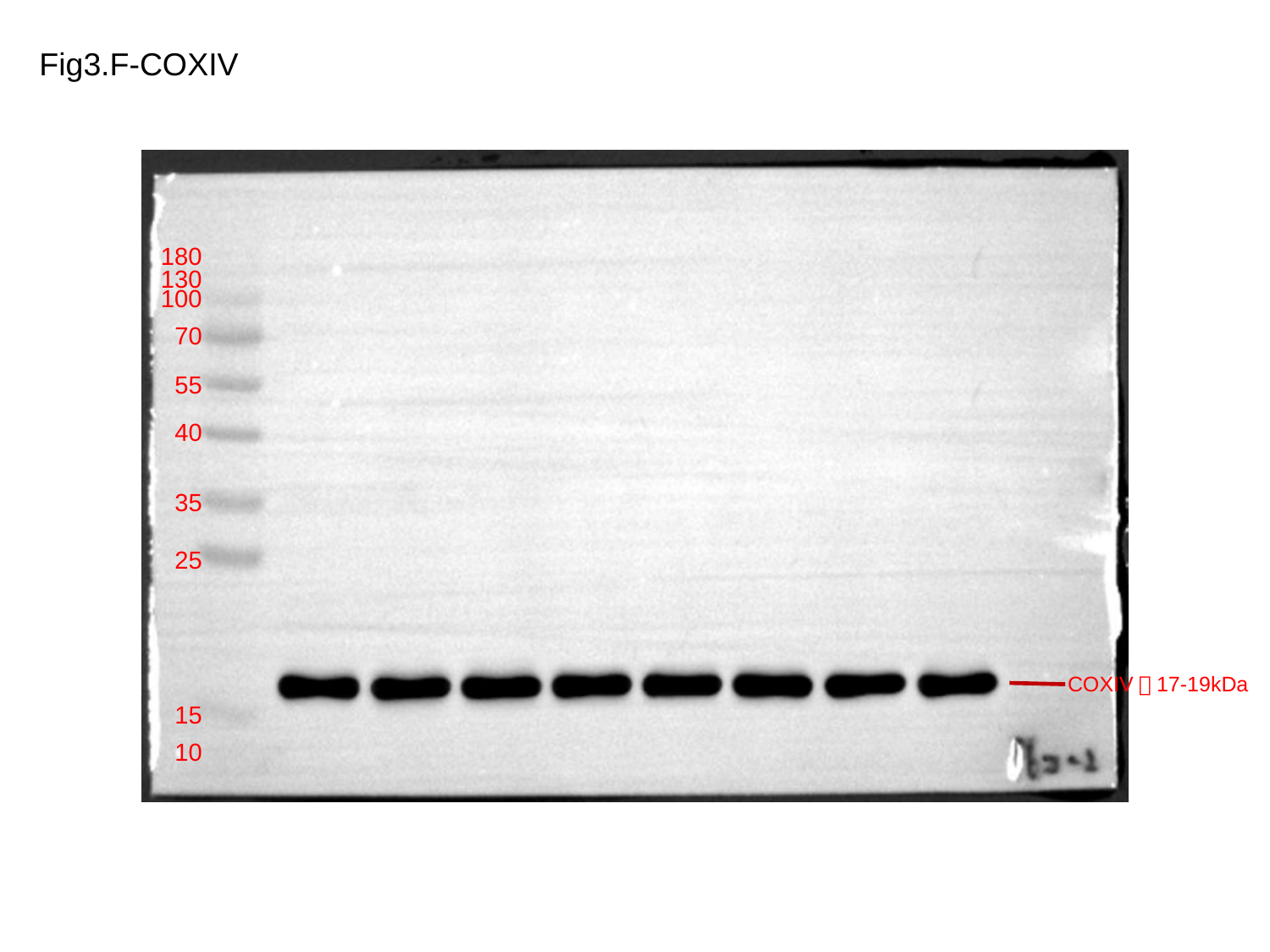

Fig3.F-COXIV
180
130
100
70
55
40
35
25
COXIV：17-19kDa
15
10

## Slide 8
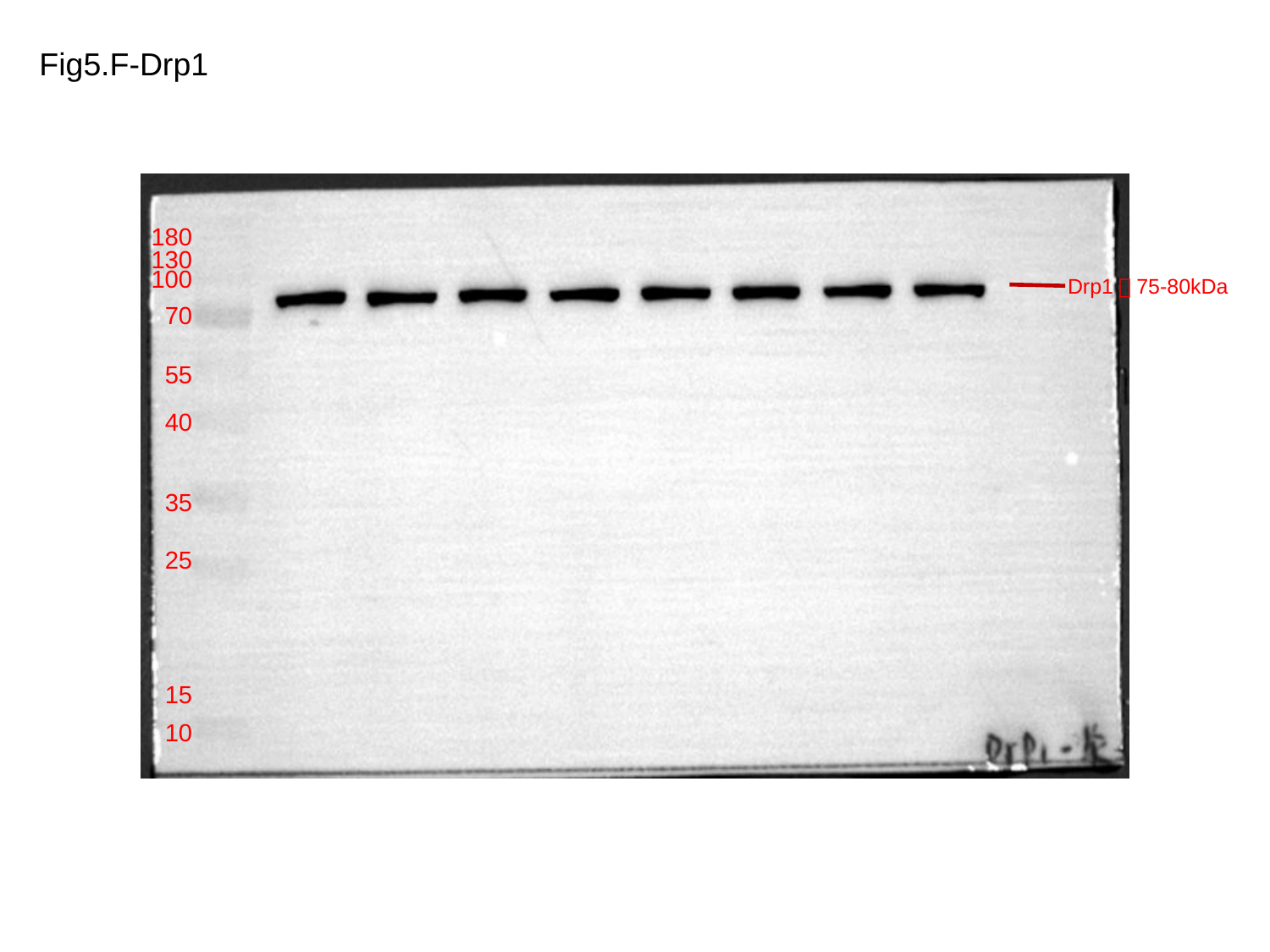

Fig5.F-Drp1
180
130
100
Drp1：75-80kDa
70
55
40
35
25
15
10

## Slide 9
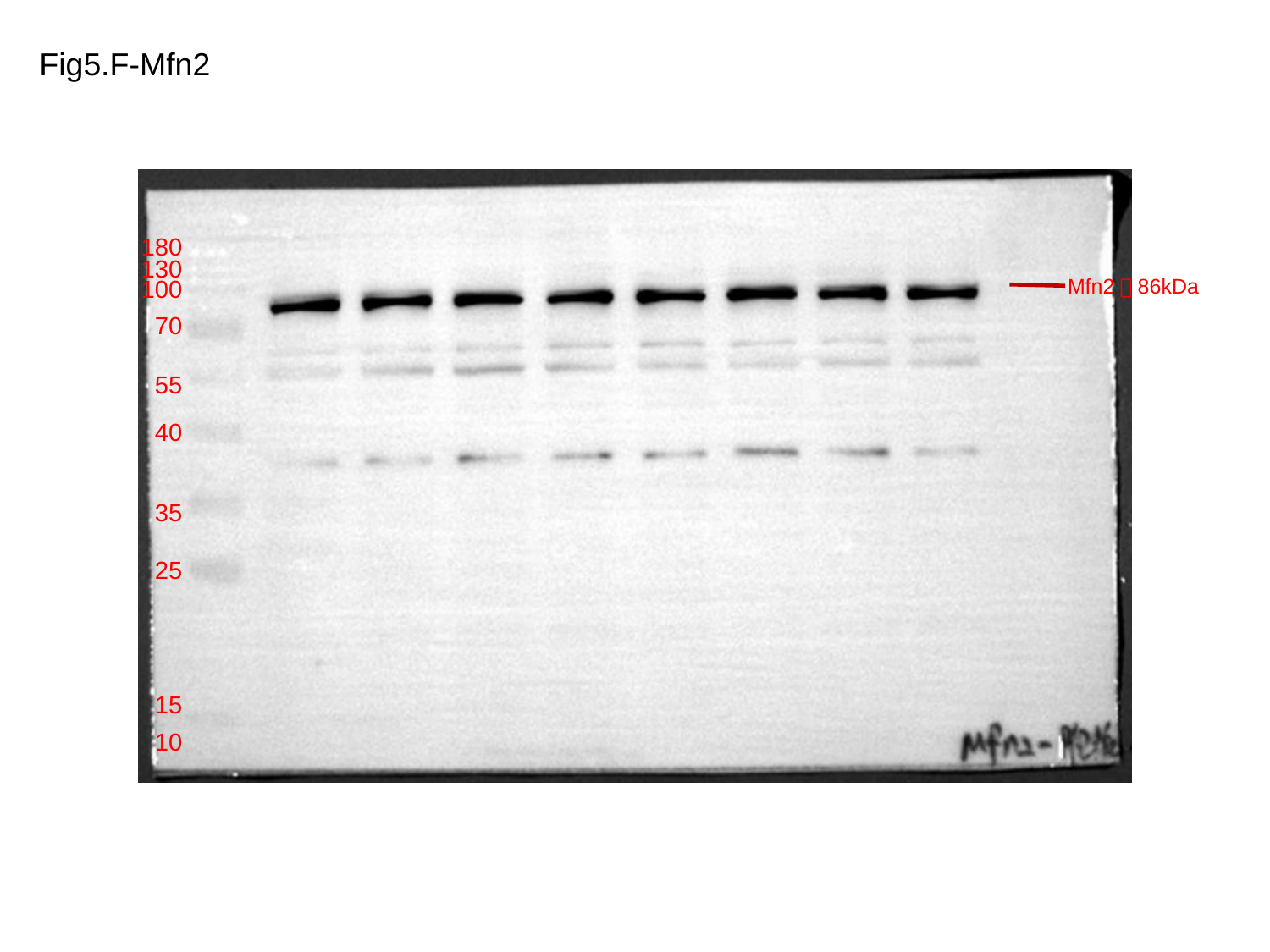

Fig5.F-Mfn2
180
130
Mfn2：86kDa
100
70
55
40
35
25
15
10

## Slide 10
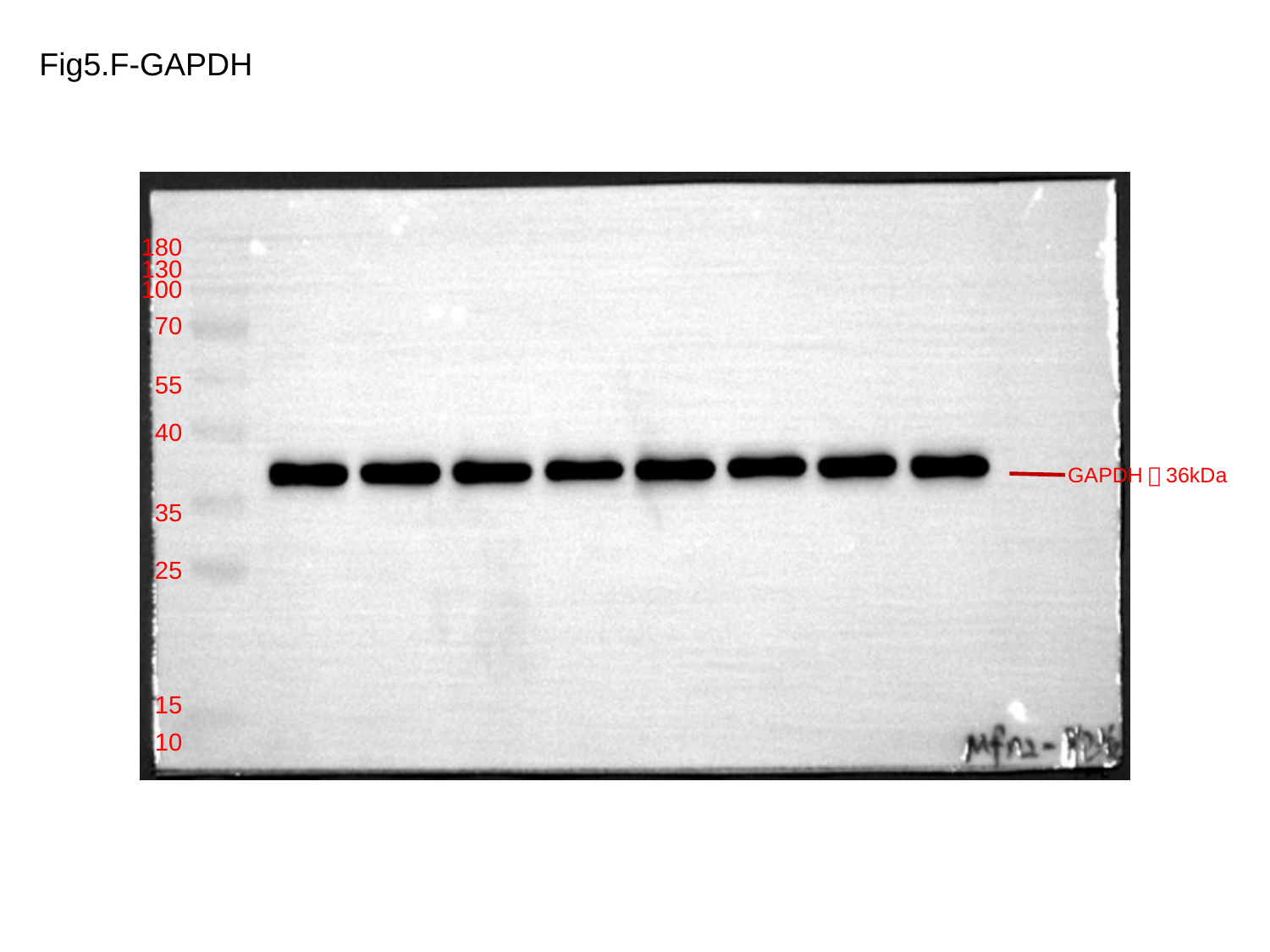

Fig5.F-GAPDH
180
130
100
70
55
40
GAPDH：36kDa
35
25
15
10

## Slide 11
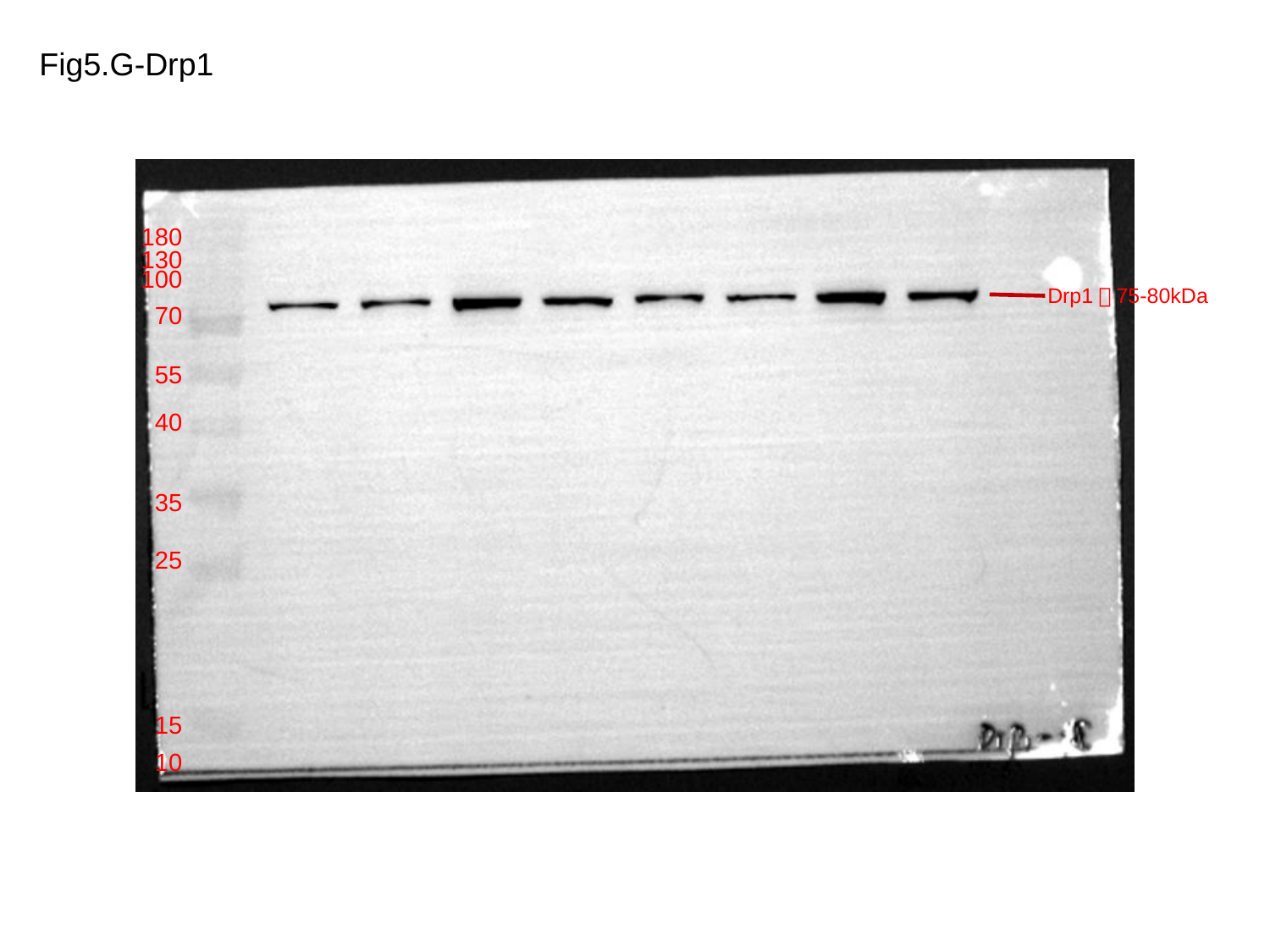

Fig5.G-Drp1
180
130
100
Drp1：75-80kDa
70
55
40
35
25
15
10

## Slide 12
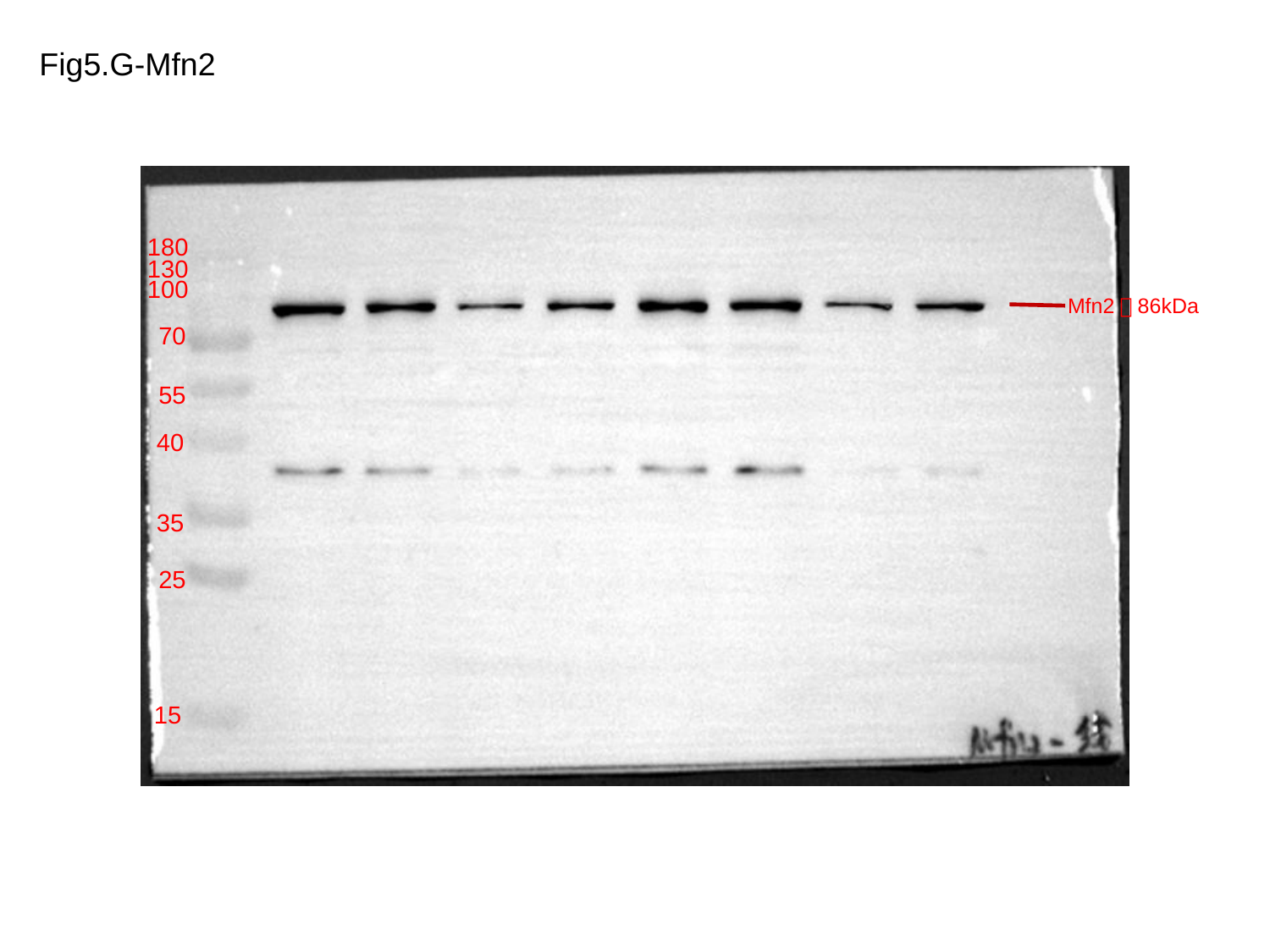

Fig5.G-Mfn2
180
130
100
Mfn2：86kDa
70
55
40
35
25
15

## Slide 13
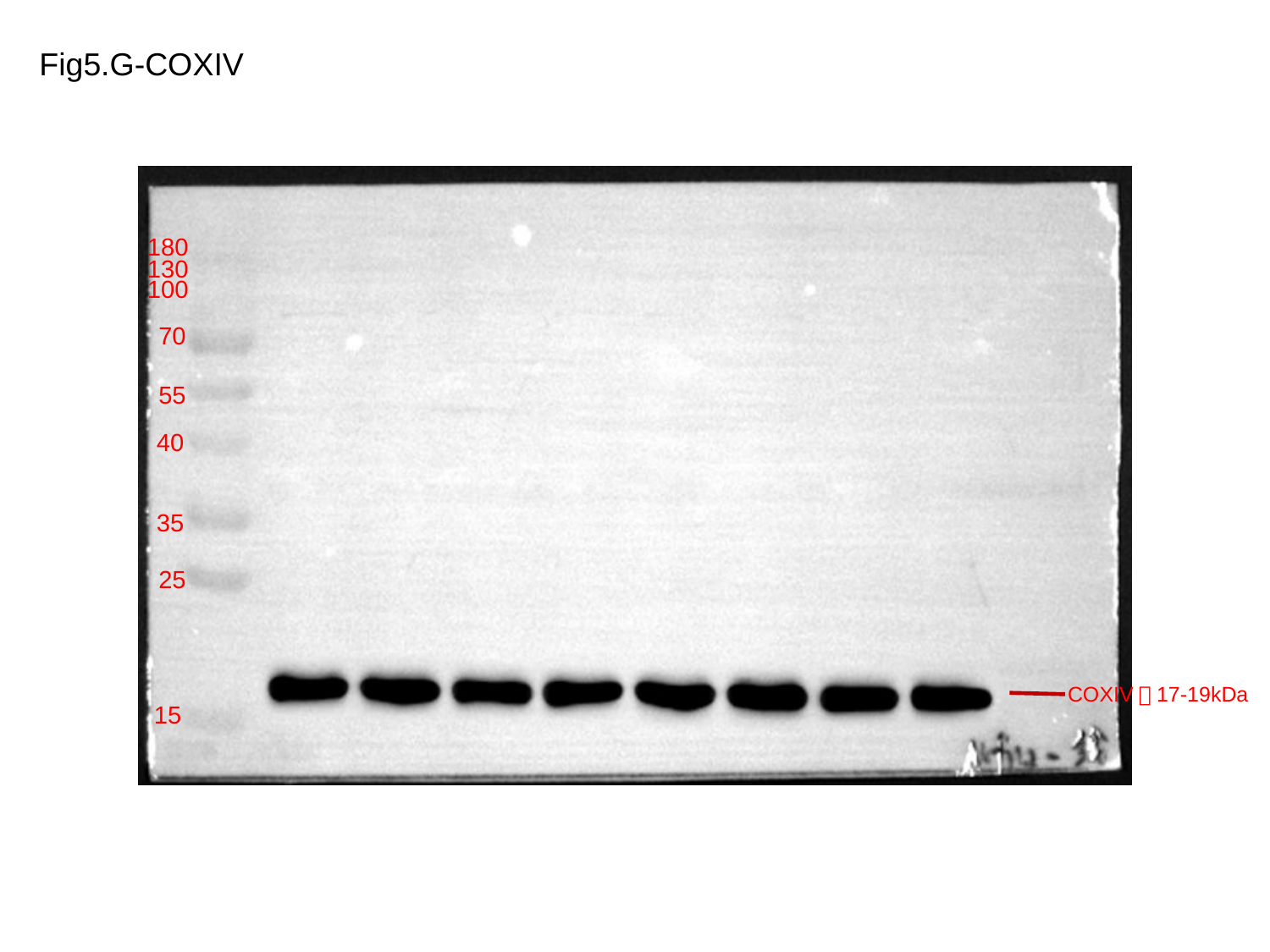

Fig5.G-COXIV
180
130
100
70
55
40
35
25
COXIV：17-19kDa
15

## Slide 14
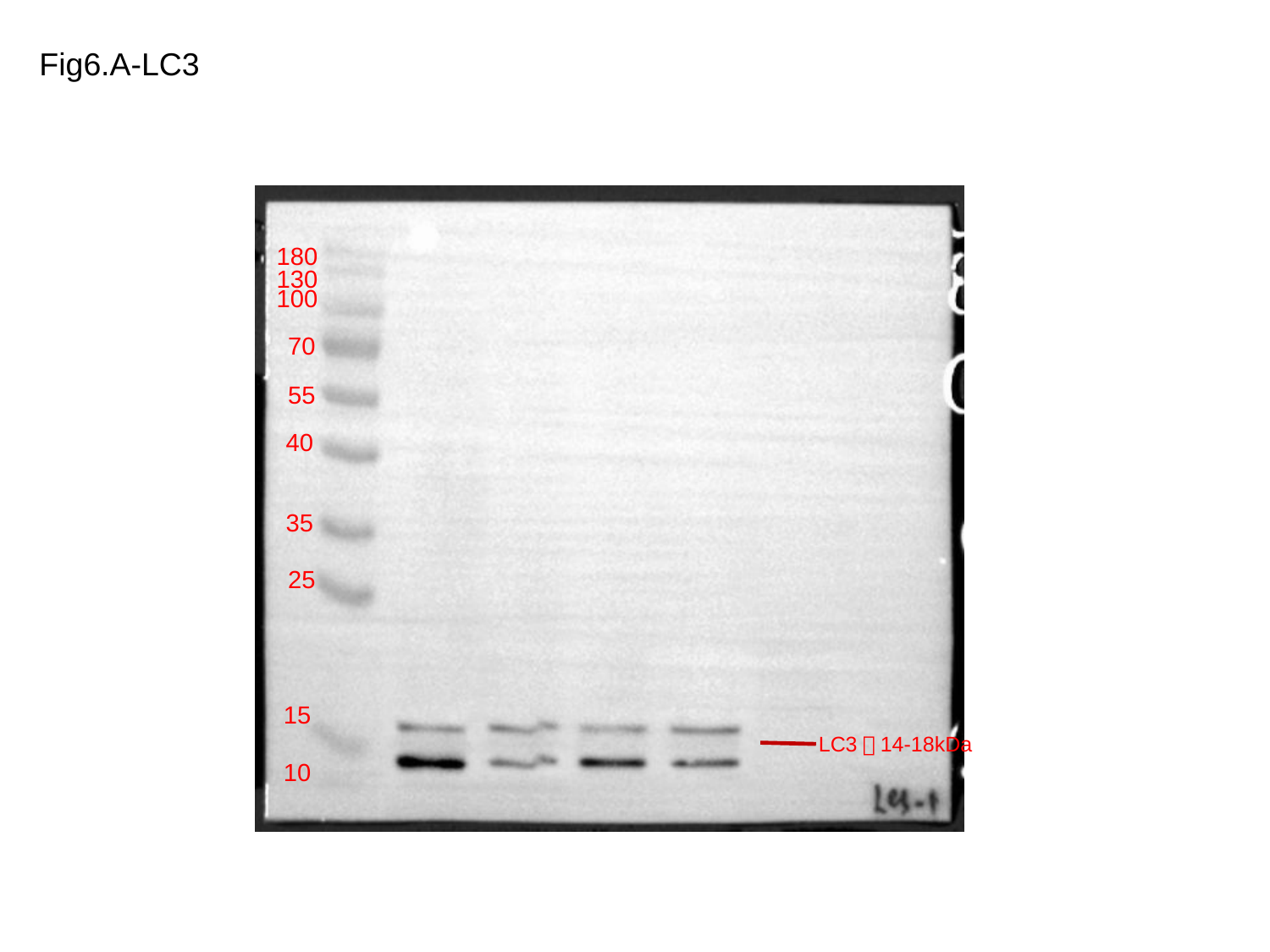

Fig6.A-LC3
180
130
100
70
55
40
35
25
15
LC3：14-18kDa
10

## Slide 15
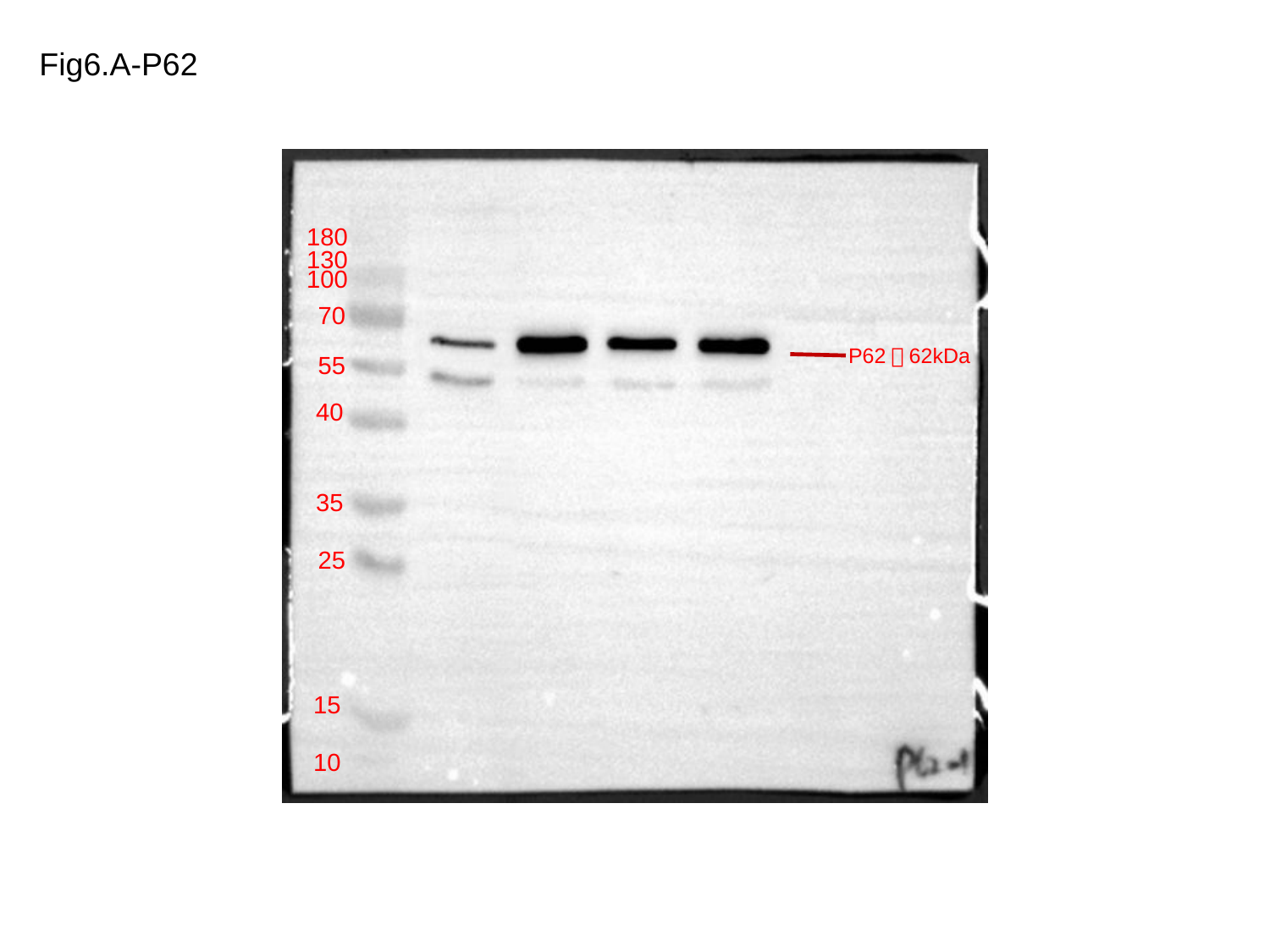

Fig6.A-P62
180
130
100
70
P62：62kDa
55
40
35
25
15
10

## Slide 16
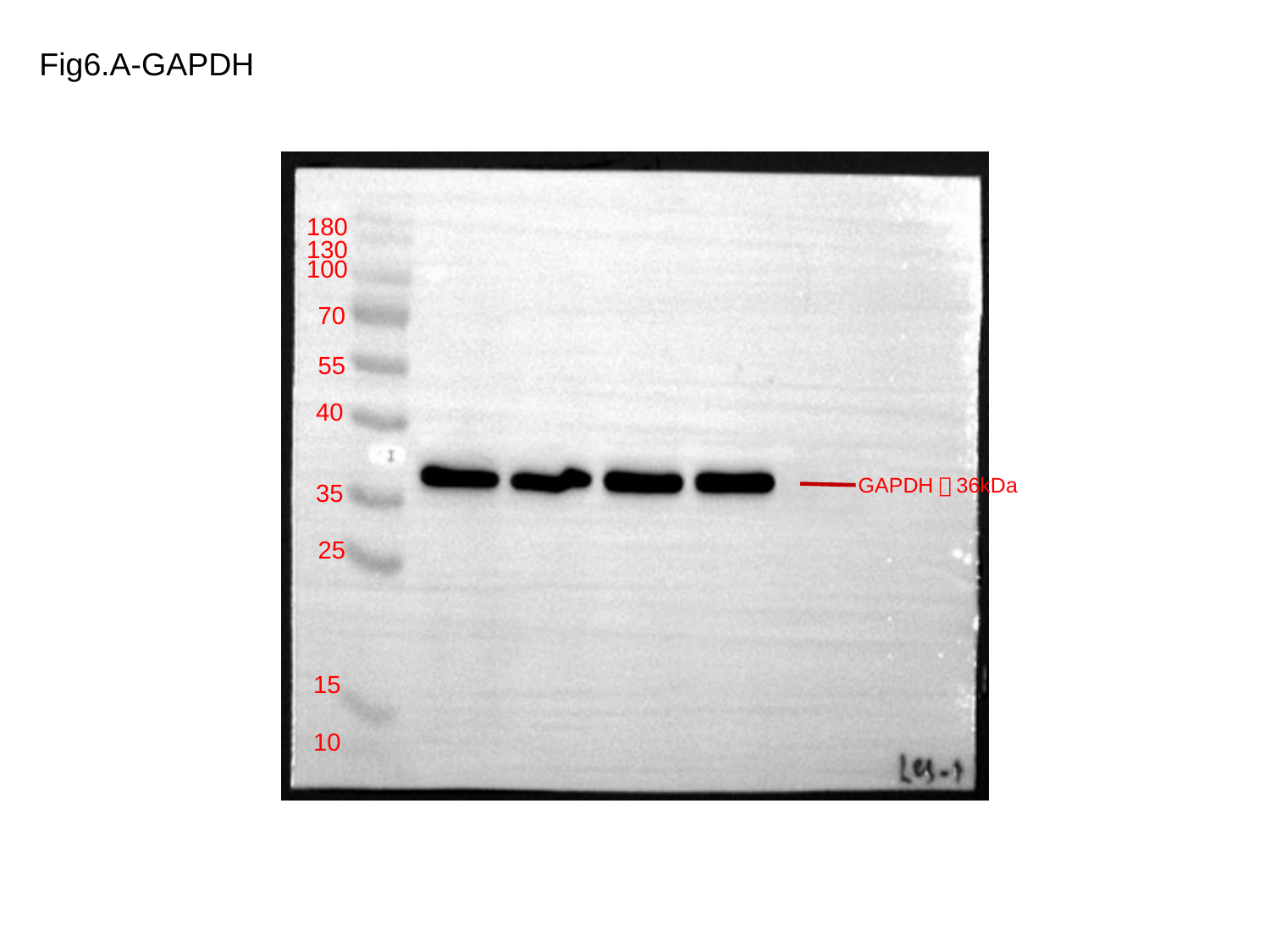

Fig6.A-GAPDH
180
130
100
70
55
40
GAPDH：36kDa
35
25
15
10

## Slide 17
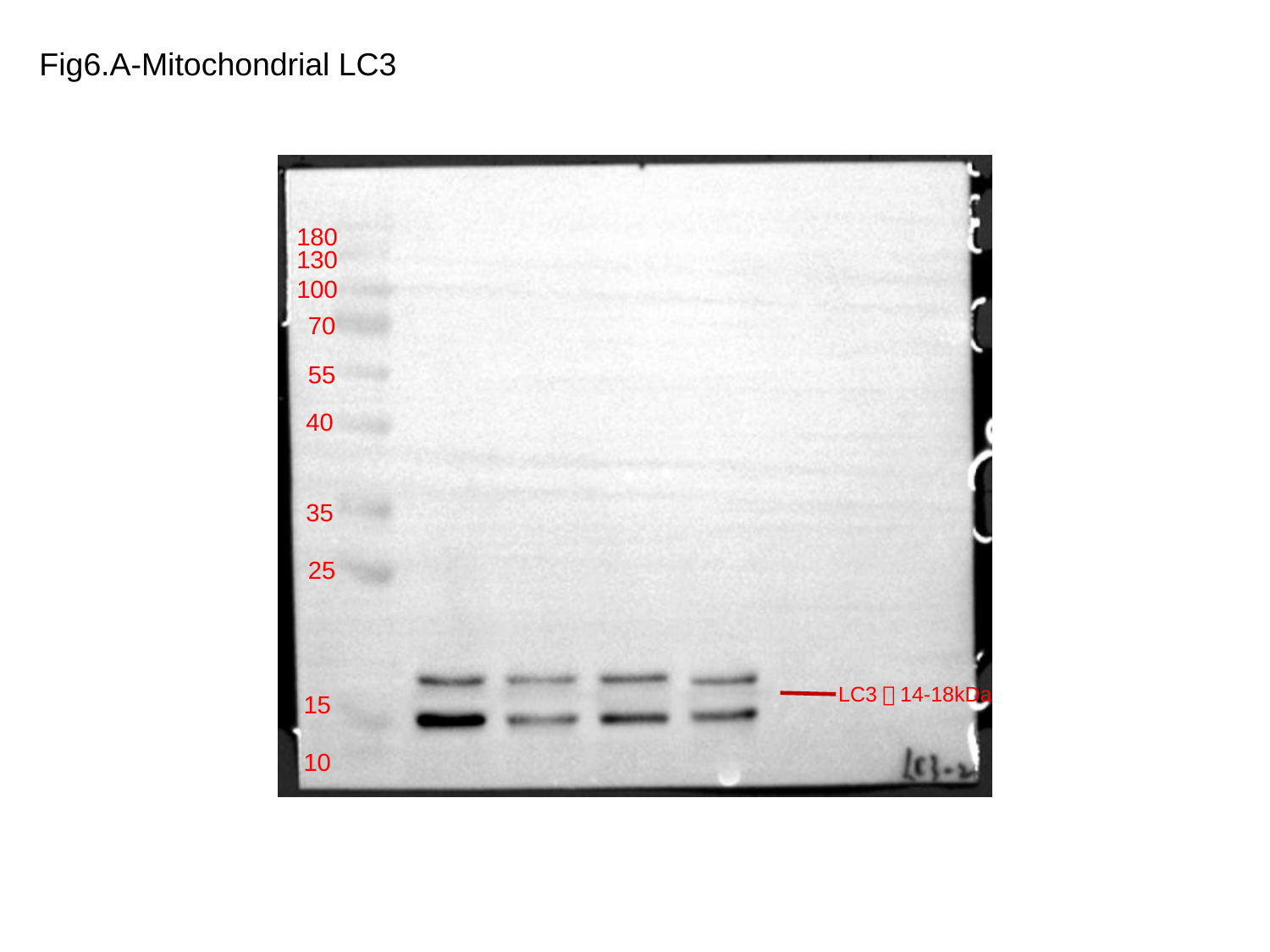

Fig6.A-Mitochondrial LC3
180
130
100
70
55
40
35
25
LC3：14-18kDa
15
10

## Slide 18
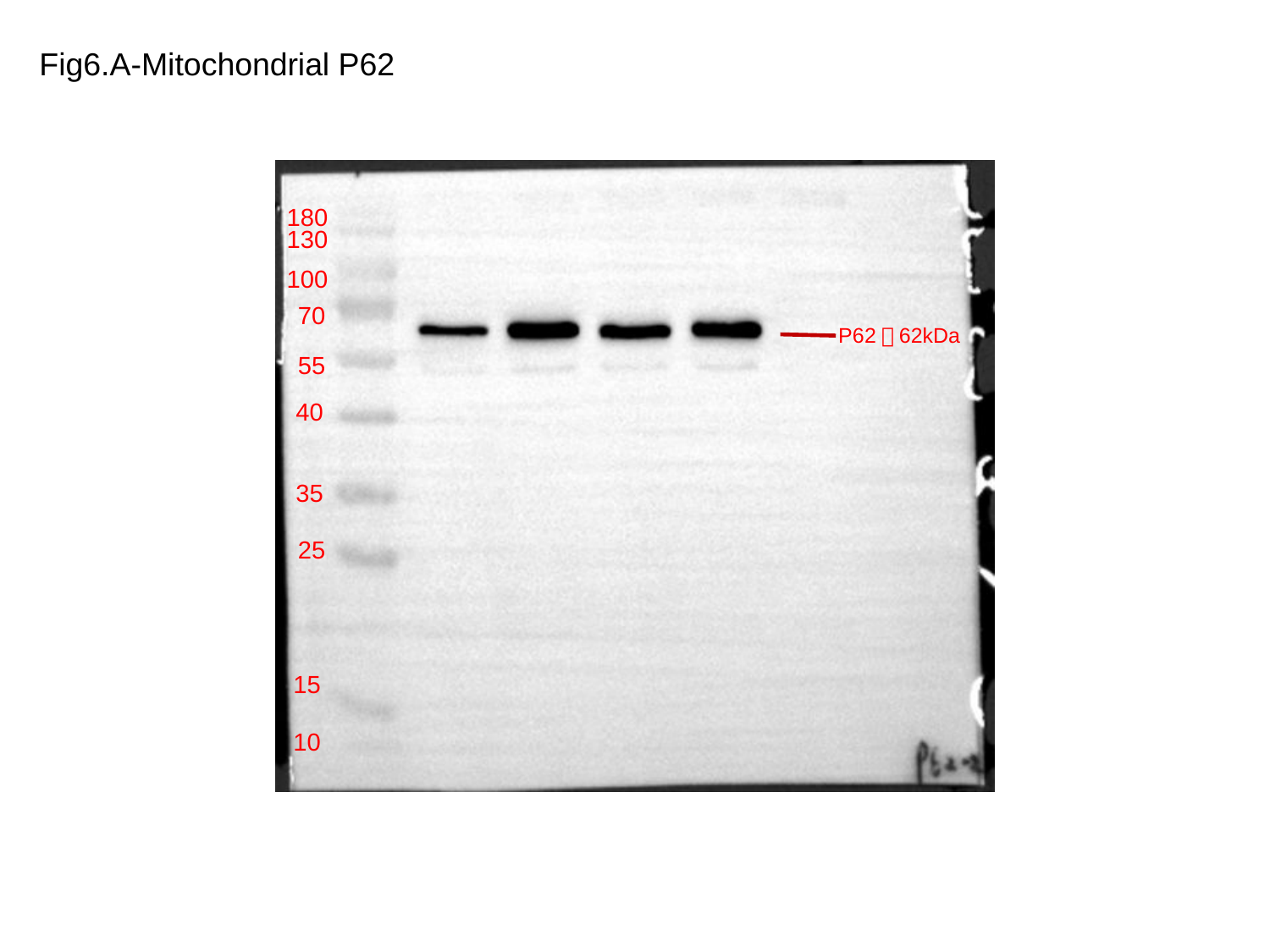

Fig6.A-Mitochondrial P62
180
130
100
70
P62：62kDa
55
40
35
25
15
10

## Slide 19
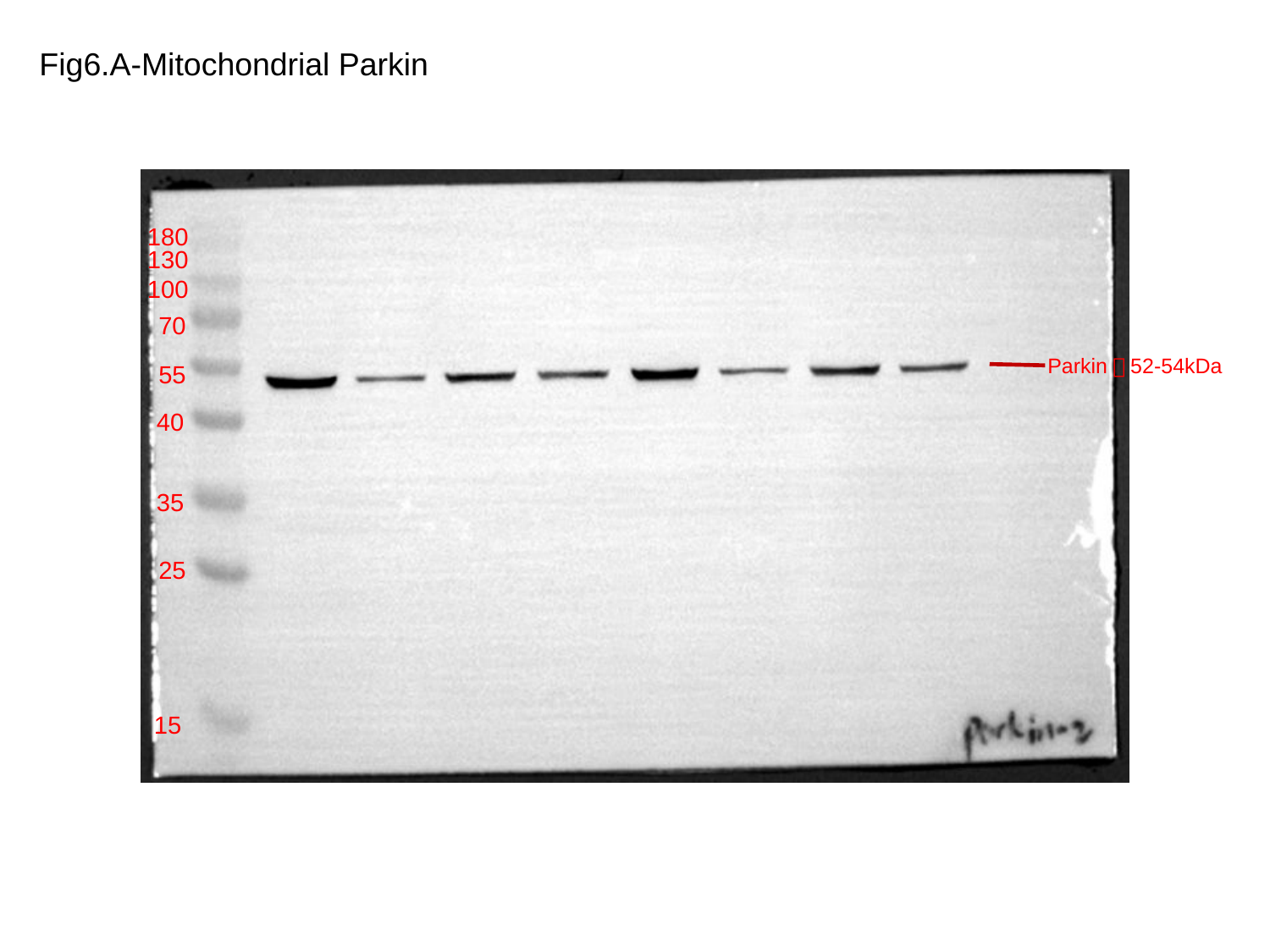

Fig6.A-Mitochondrial Parkin
180
130
100
70
Parkin：52-54kDa
55
40
35
25
15

## Slide 20
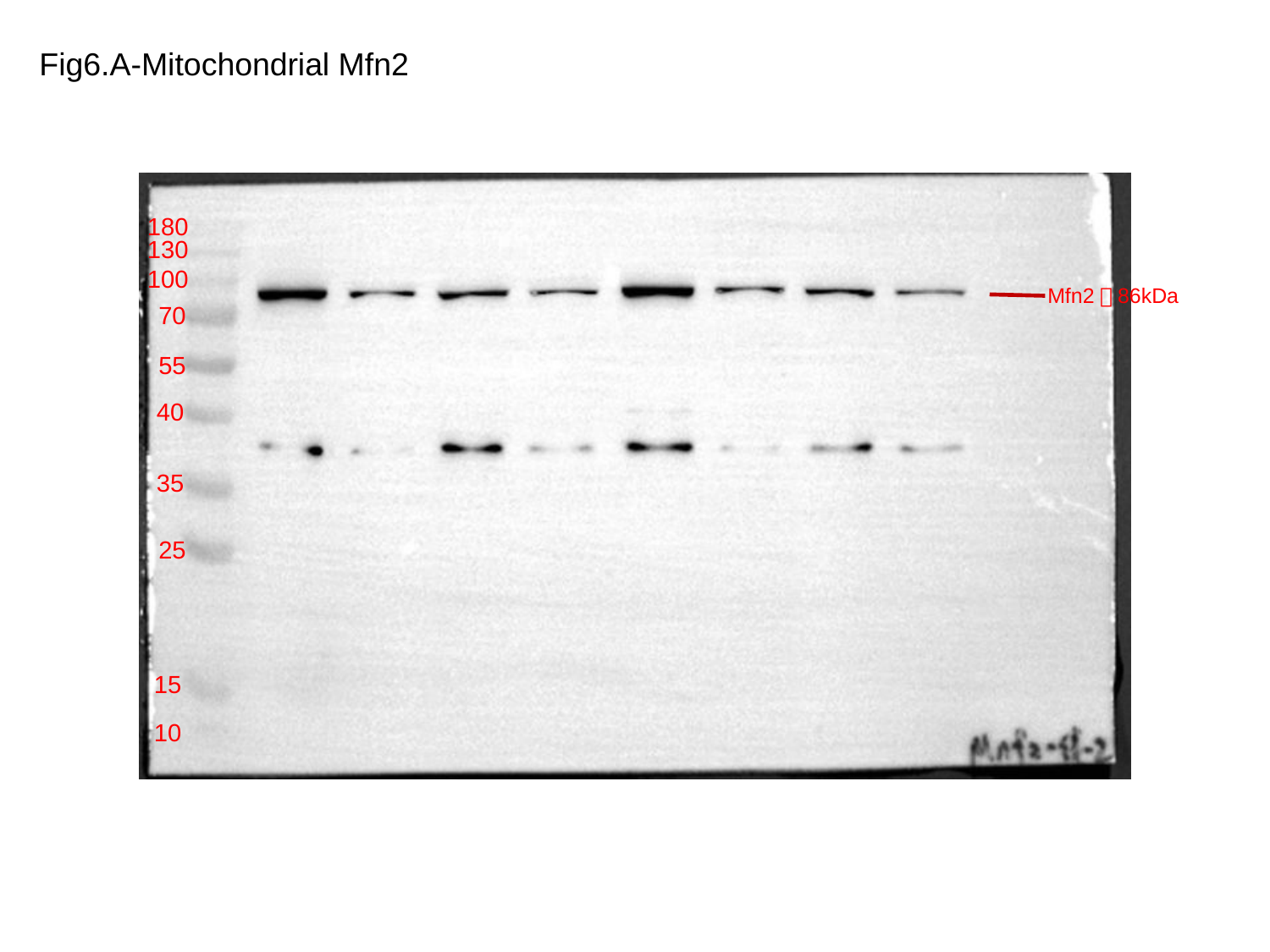

Fig6.A-Mitochondrial Mfn2
180
130
100
Mfn2：86kDa
70
55
40
35
25
15
10

## Slide 21
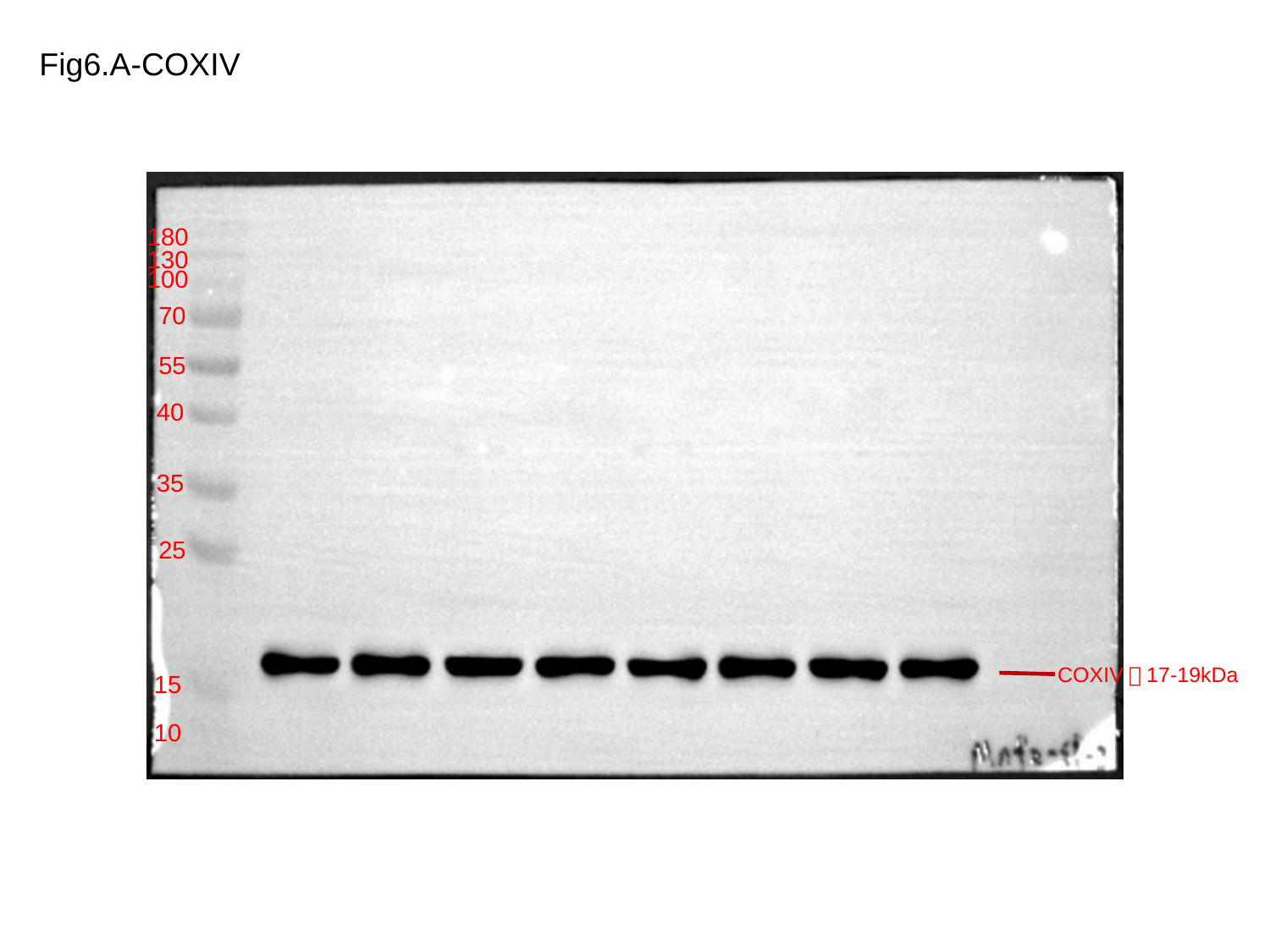

Fig6.A-COXIV
180
130
100
70
55
40
35
25
COXIV：17-19kDa
15
10

## Slide 22
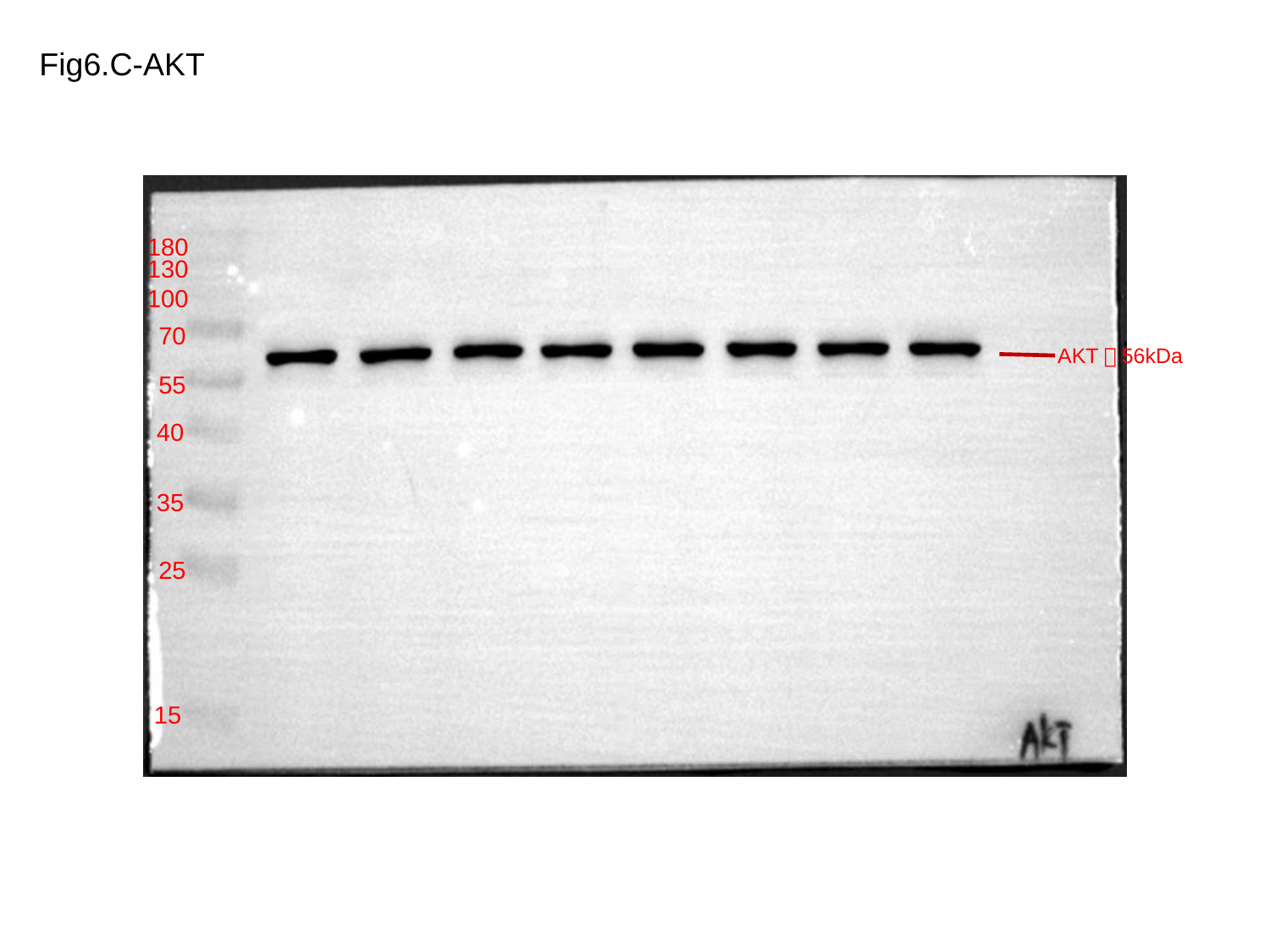

Fig6.C-AKT
180
130
100
70
AKT：56kDa
55
40
35
25
15

## Slide 23
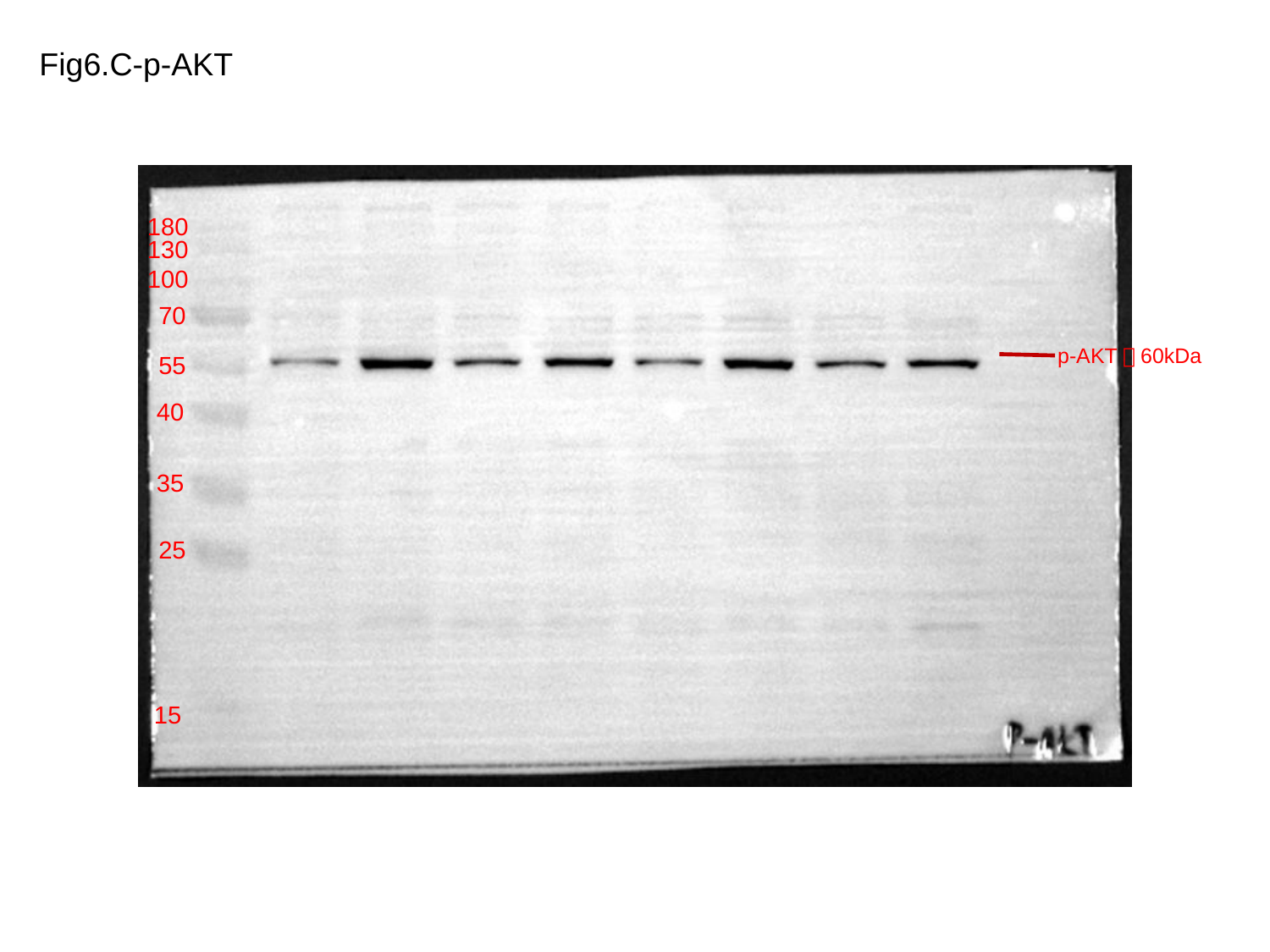

Fig6.C-p-AKT
180
130
100
70
p-AKT：60kDa
55
40
35
25
15

## Slide 24
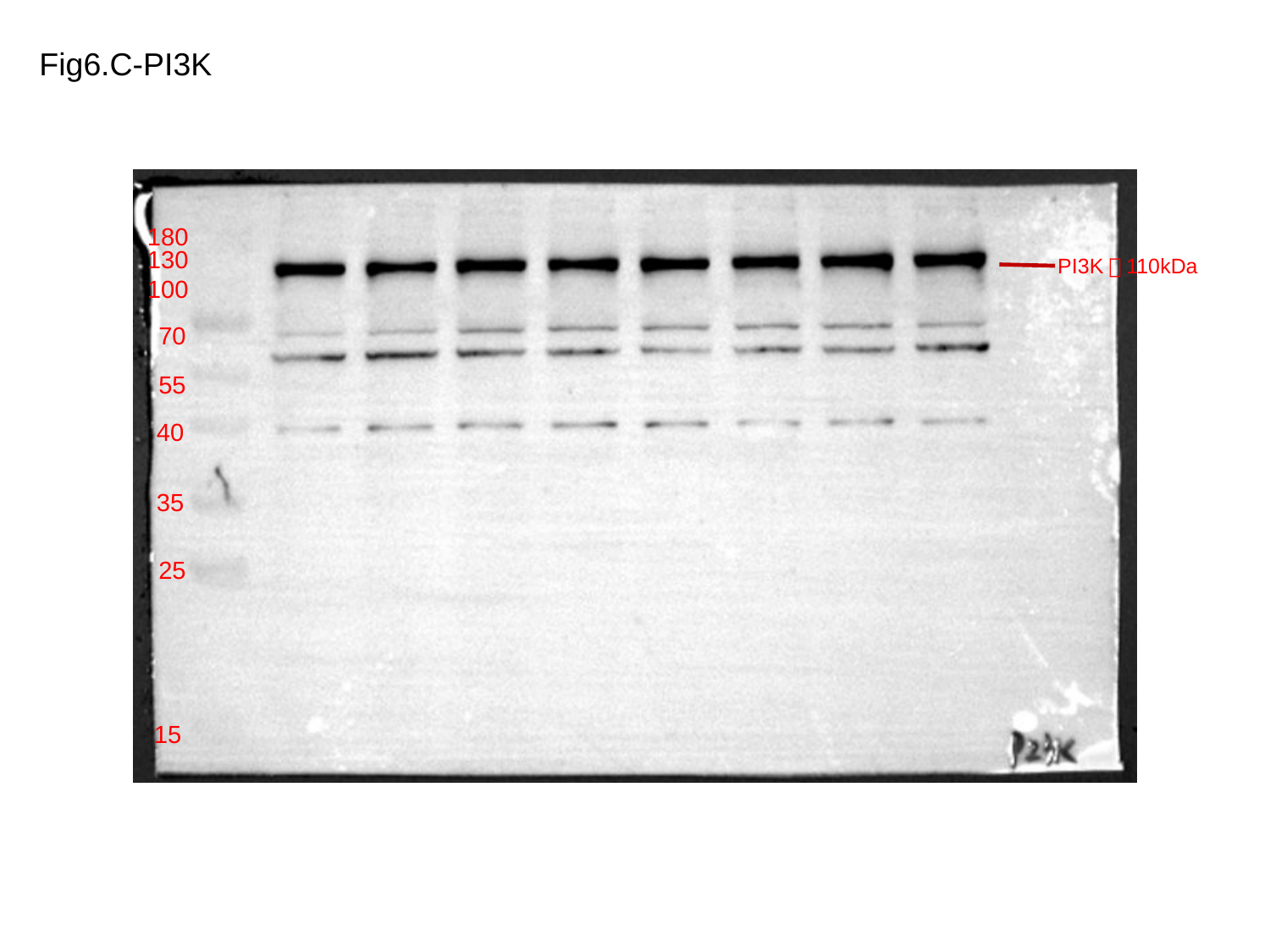

Fig6.C-PI3K
180
130
PI3K：110kDa
100
70
55
40
35
25
15

## Slide 25
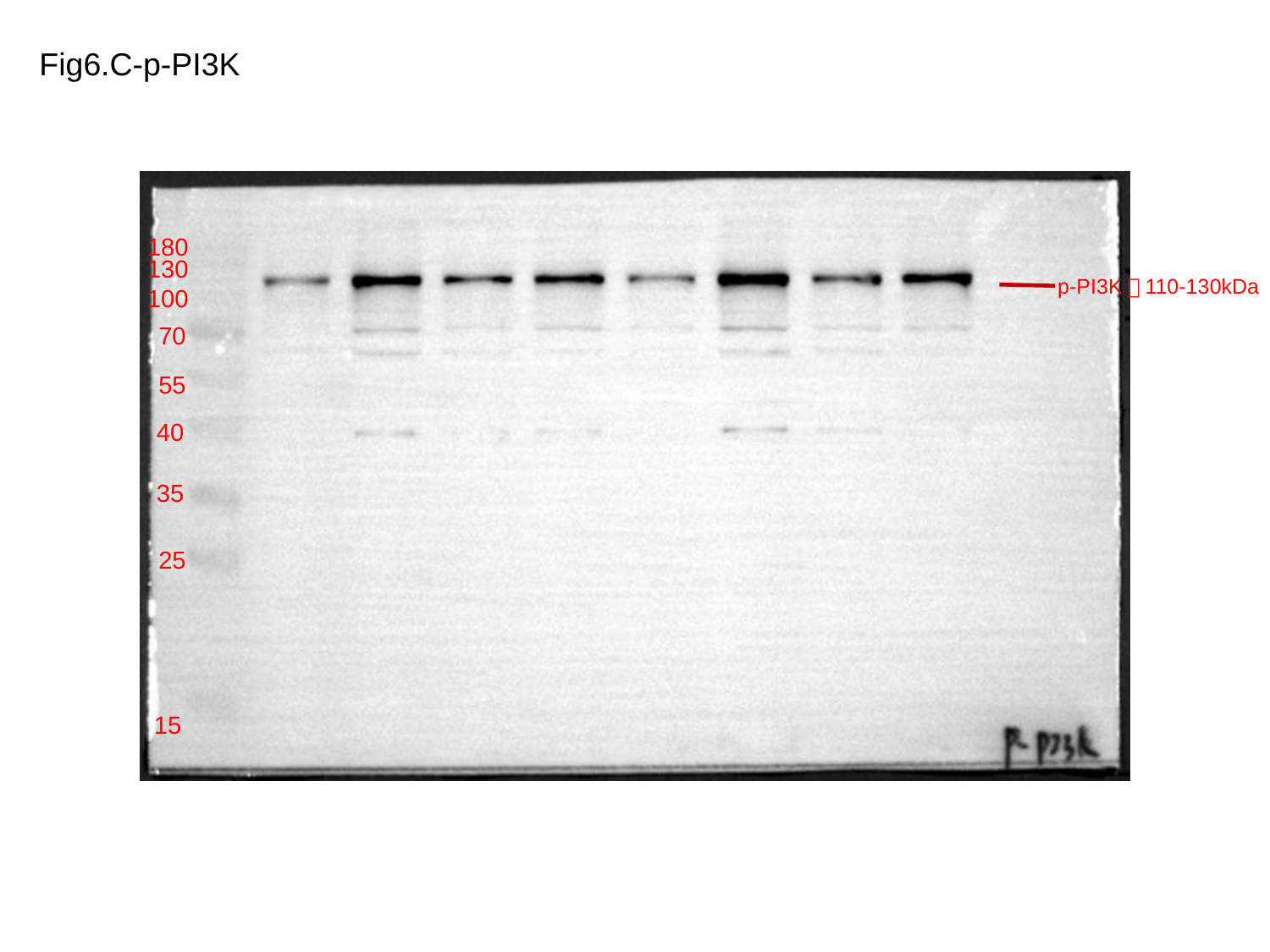

Fig6.C-p-PI3K
180
130
p-PI3K：110-130kDa
100
70
55
40
35
25
15

## Slide 26
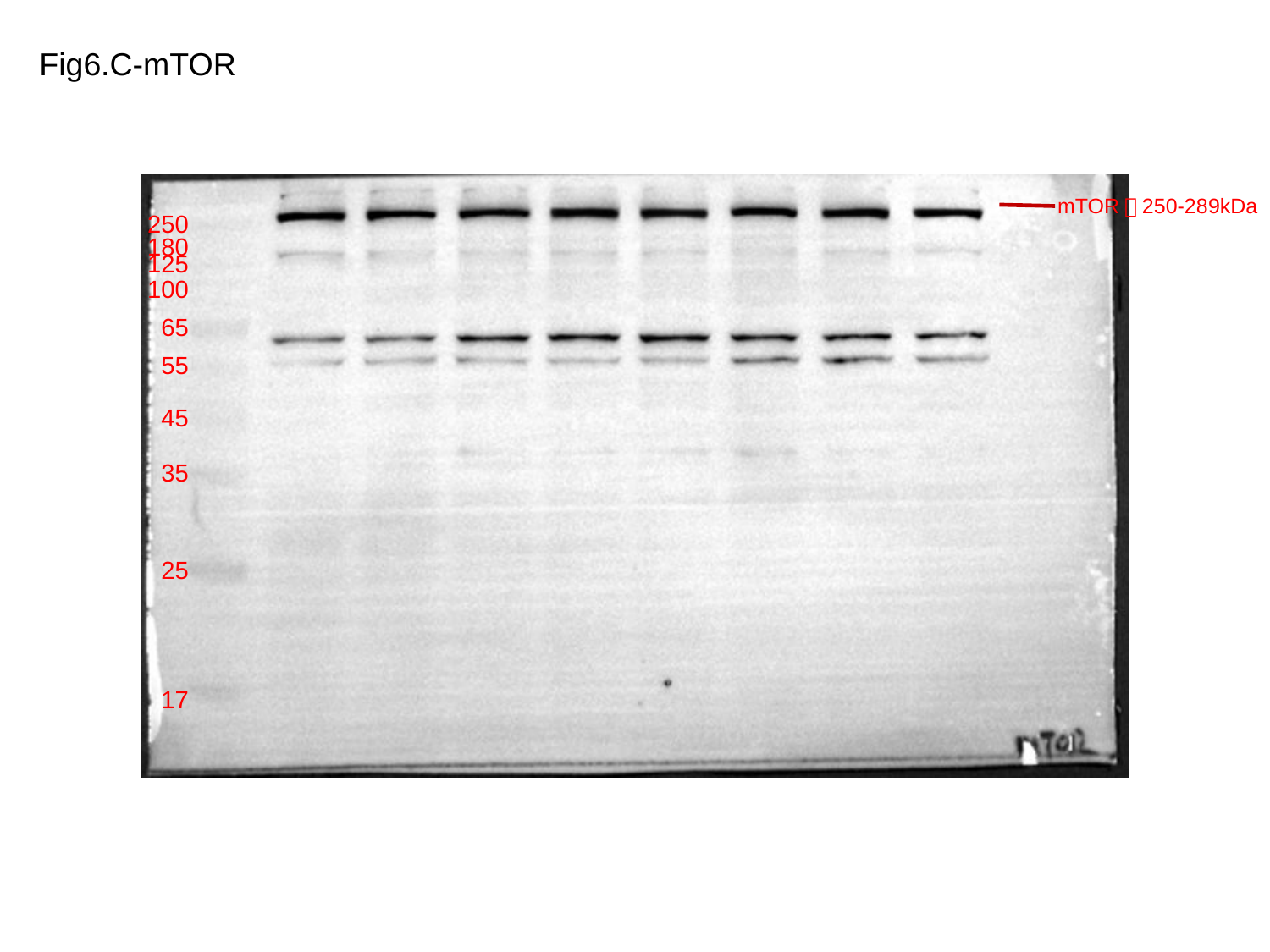

Fig6.C-mTOR
mTOR：250-289kDa
250
180
125
100
65
55
45
35
25
17

## Slide 27
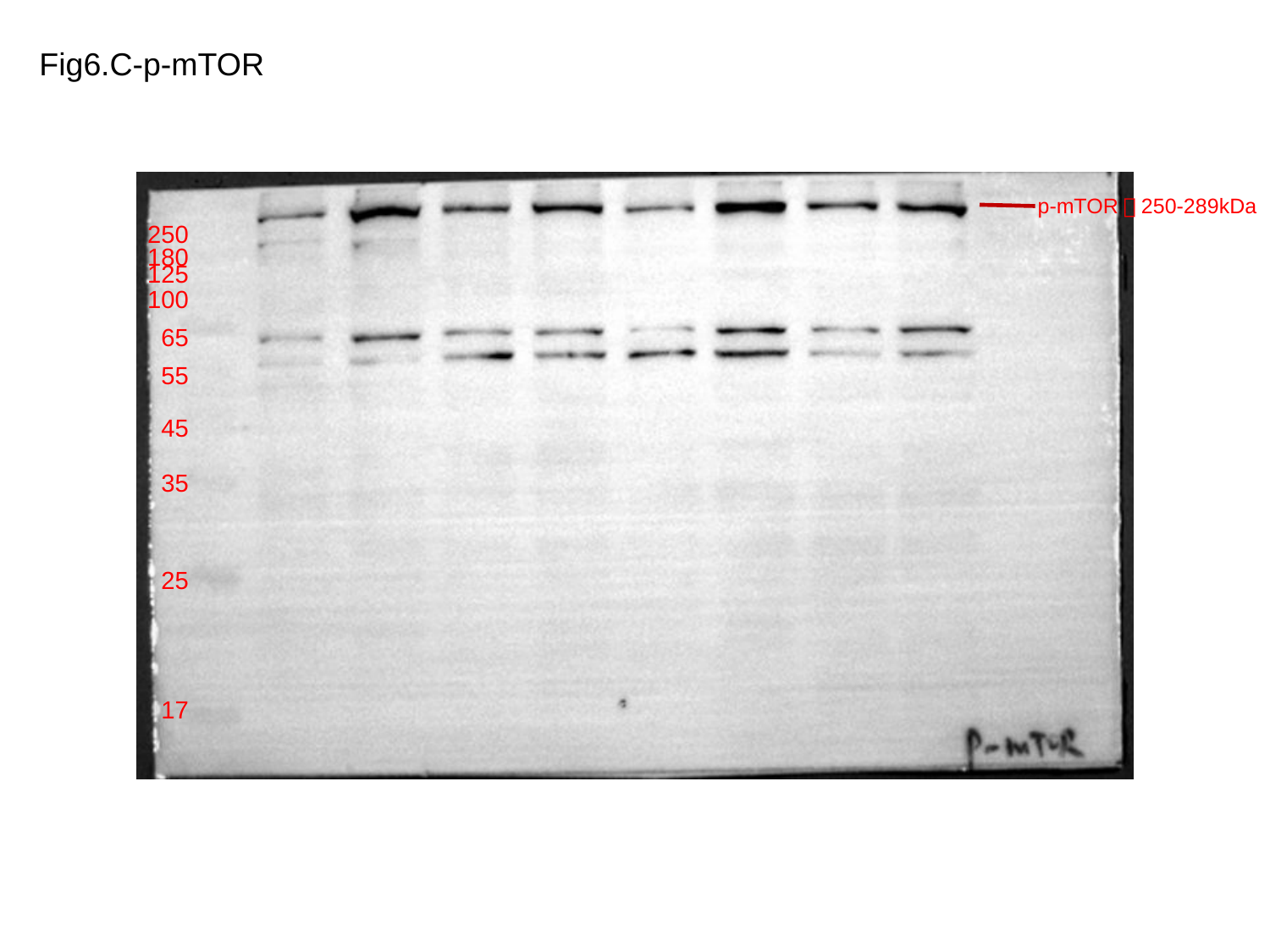

Fig6.C-p-mTOR
p-mTOR：250-289kDa
250
180
125
100
65
55
45
35
25
17

## Slide 28
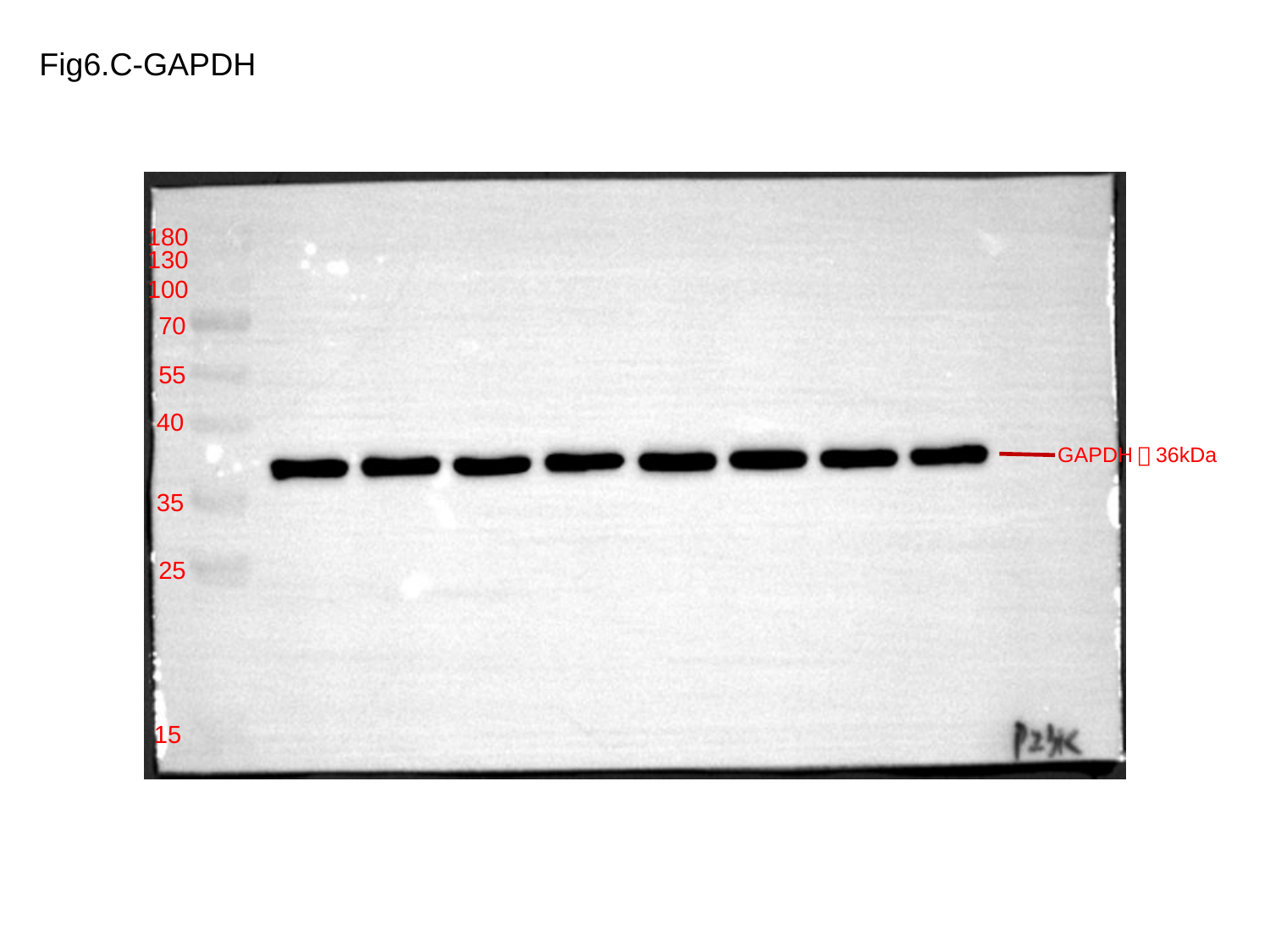

Fig6.C-GAPDH
180
130
100
70
55
40
GAPDH：36kDa
35
25
15

## Slide 29
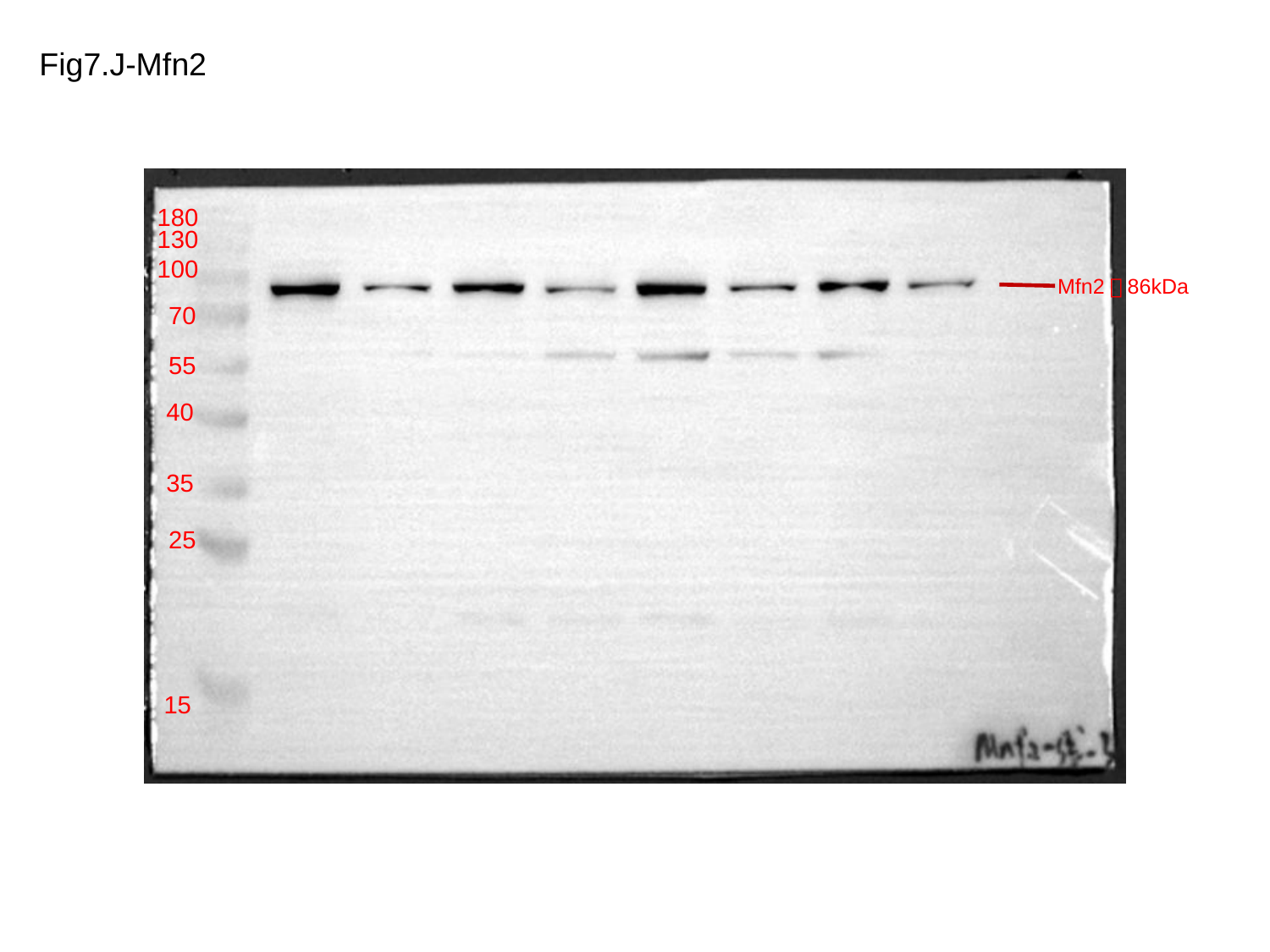

Fig7.J-Mfn2
180
130
100
Mfn2：86kDa
70
55
40
35
25
15

## Slide 30
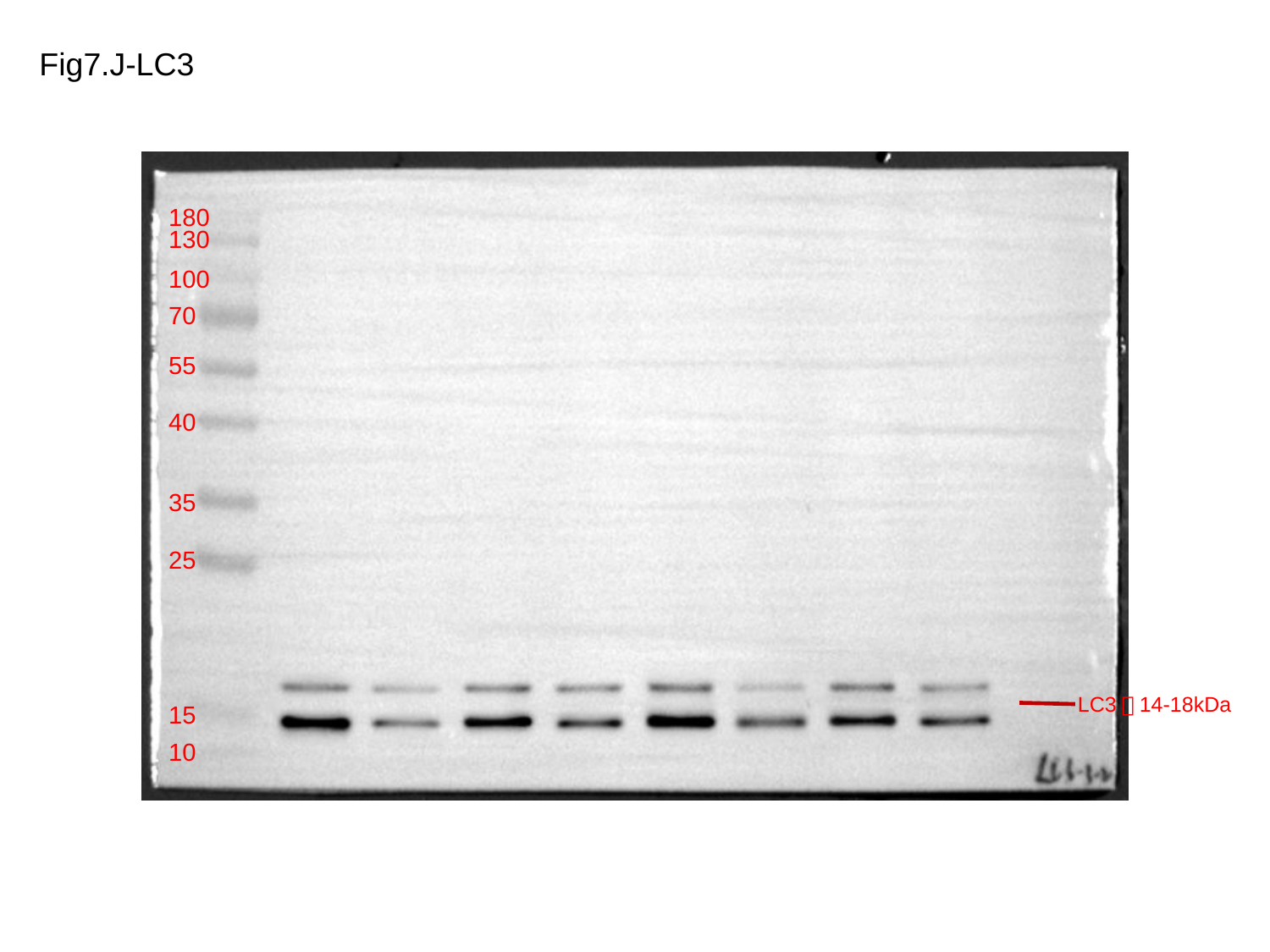

Fig7.J-LC3
180
130
100
70
55
40
35
25
LC3：14-18kDa
15
10

## Slide 31
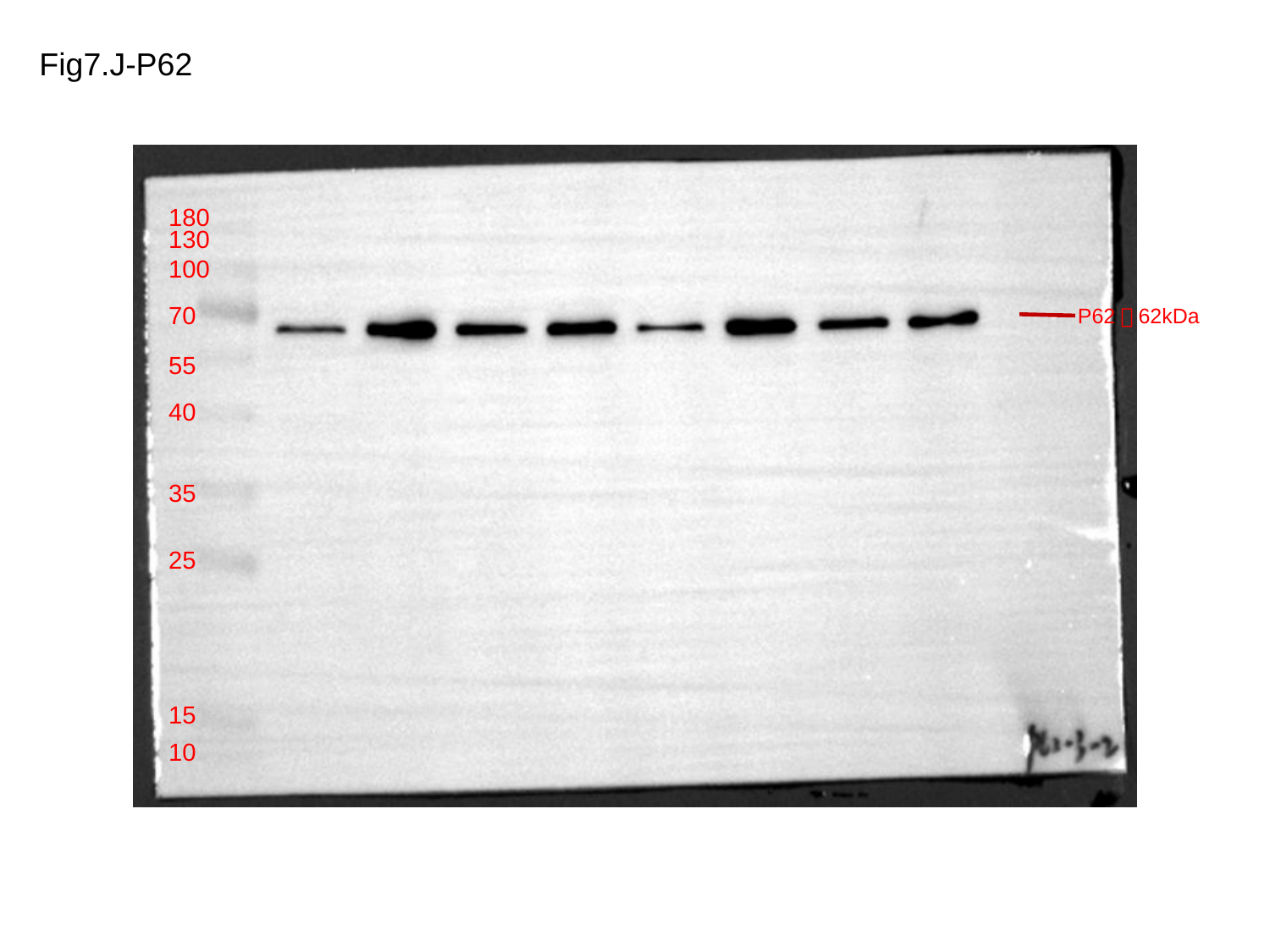

Fig7.J-P62
180
130
100
70
P62：62kDa
55
40
35
25
15
10

## Slide 32
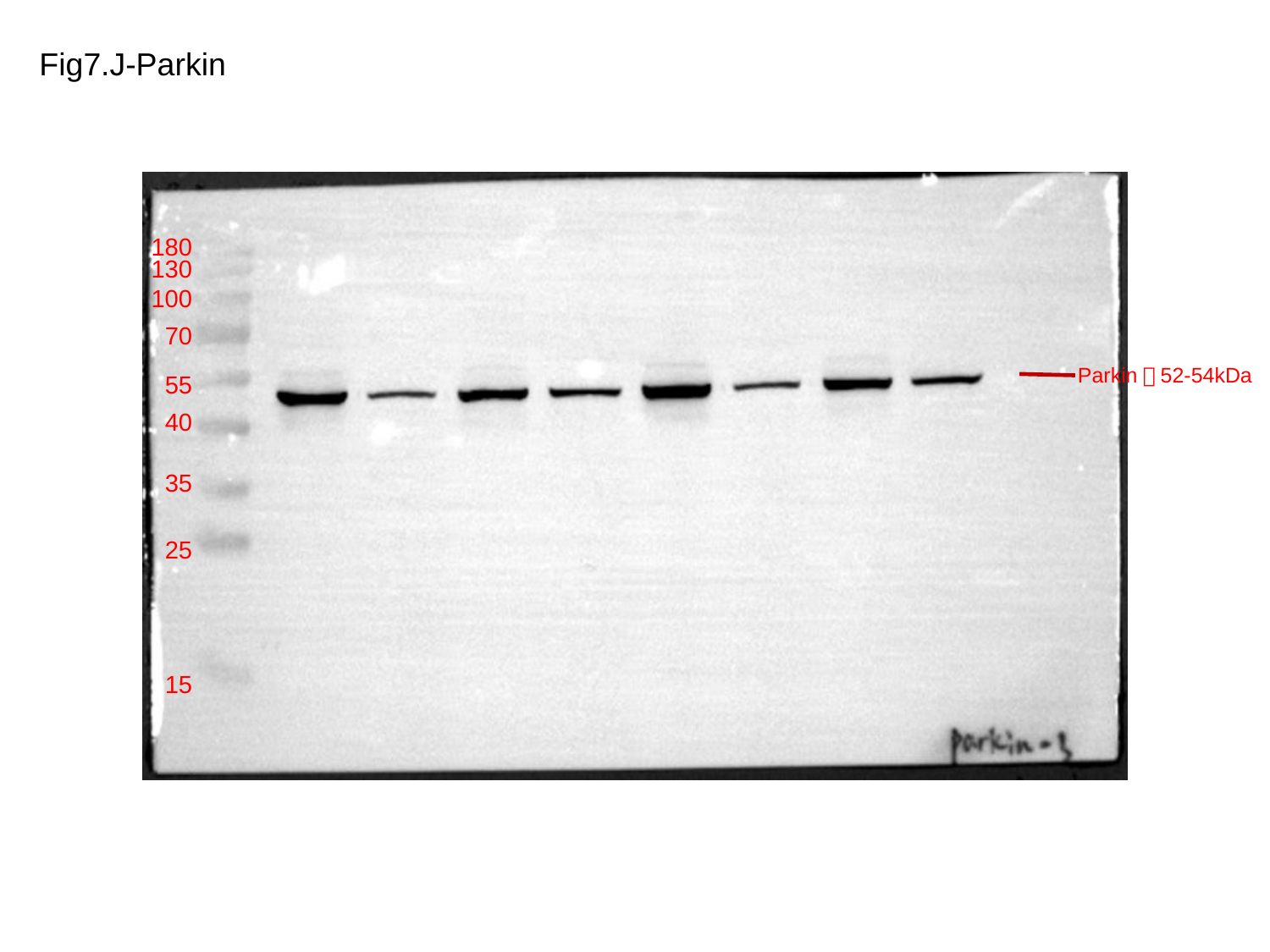

Fig7.J-Parkin
180
130
100
70
Parkin：52-54kDa
55
40
35
25
15

## Slide 33
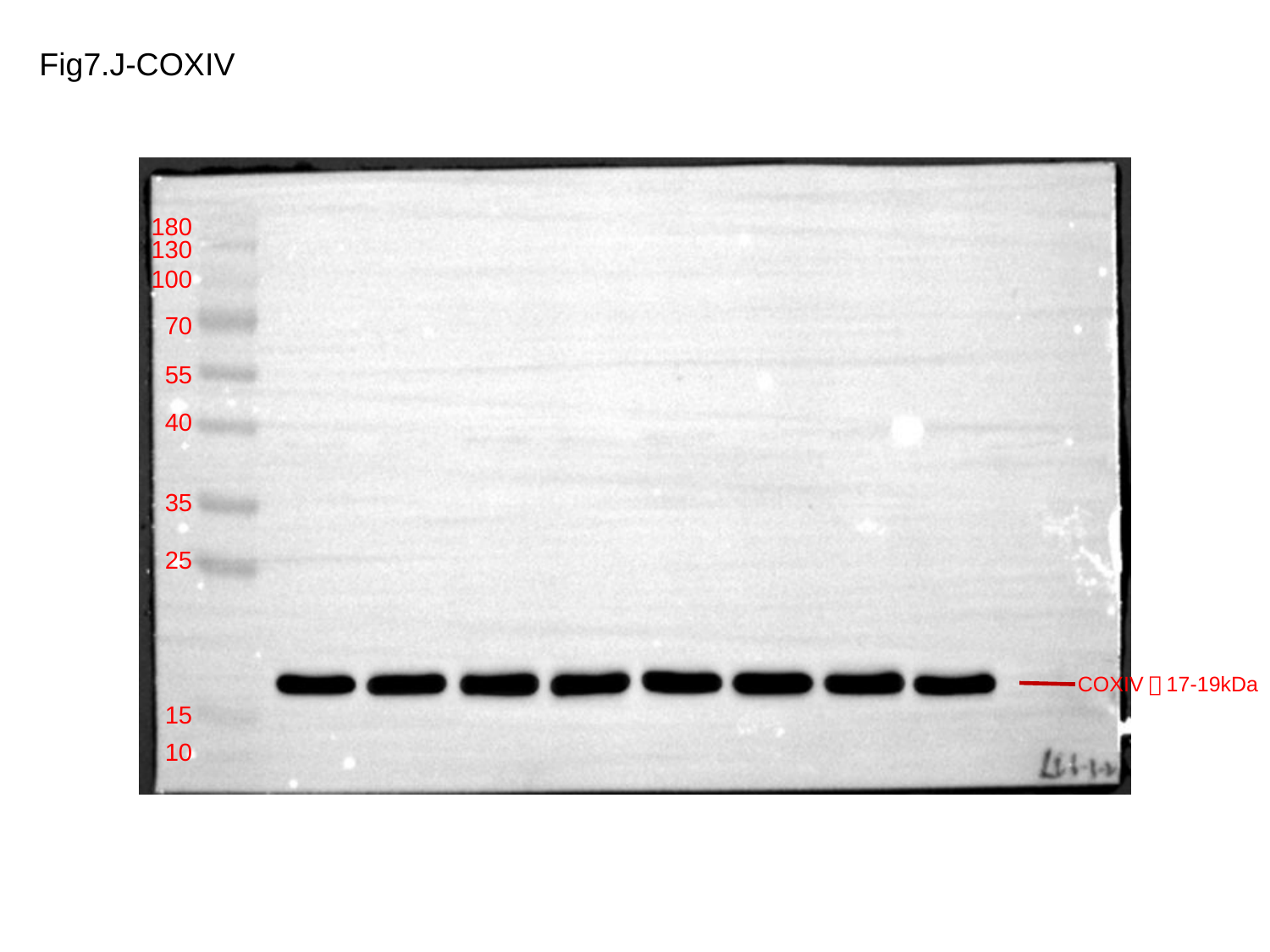

Fig7.J-COXIV
180
130
100
70
55
40
35
25
COXIV：17-19kDa
15
10

## Slide 34
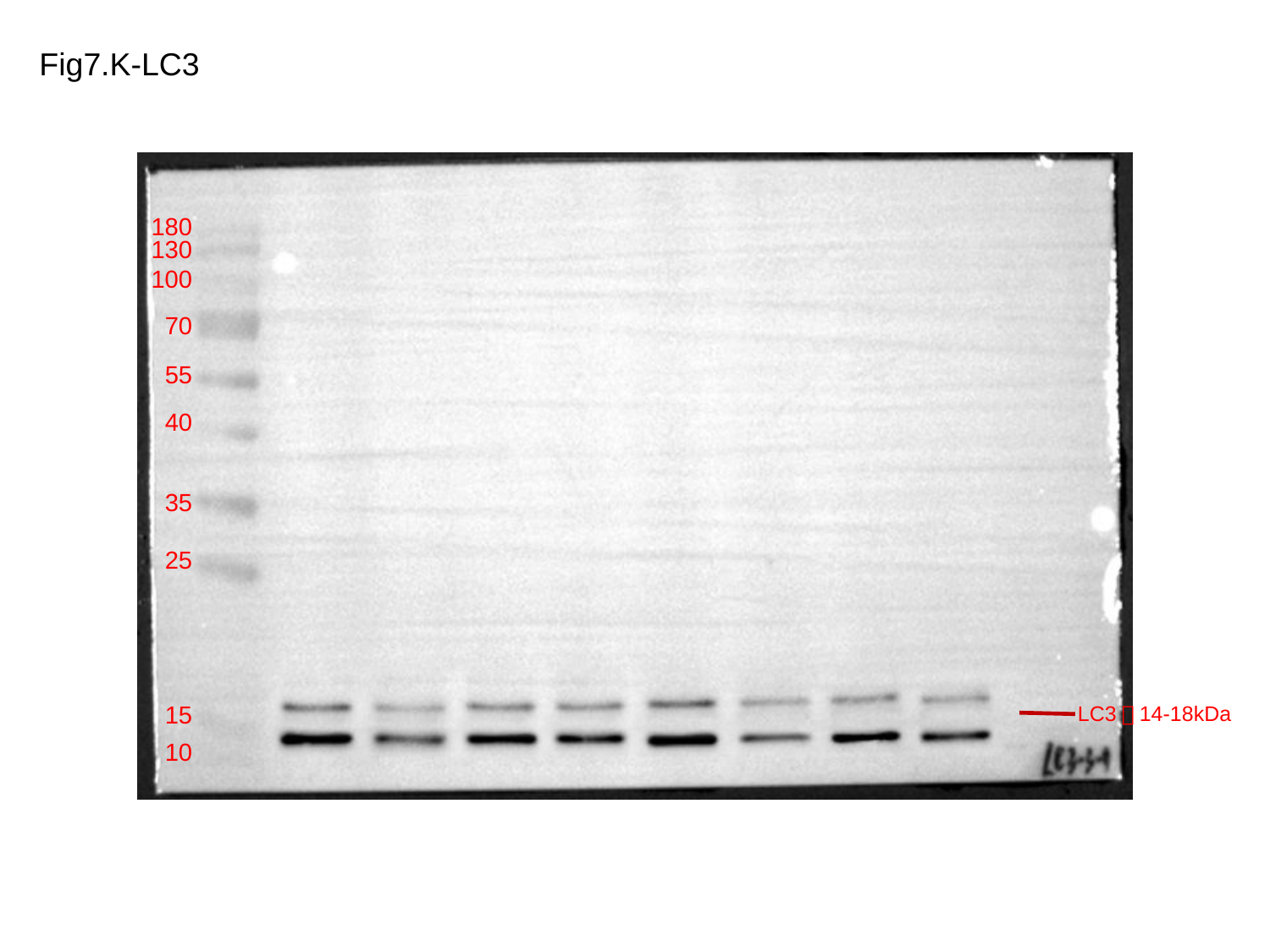

Fig7.K-LC3
180
130
100
70
55
40
35
25
15
LC3：14-18kDa
10

## Slide 35
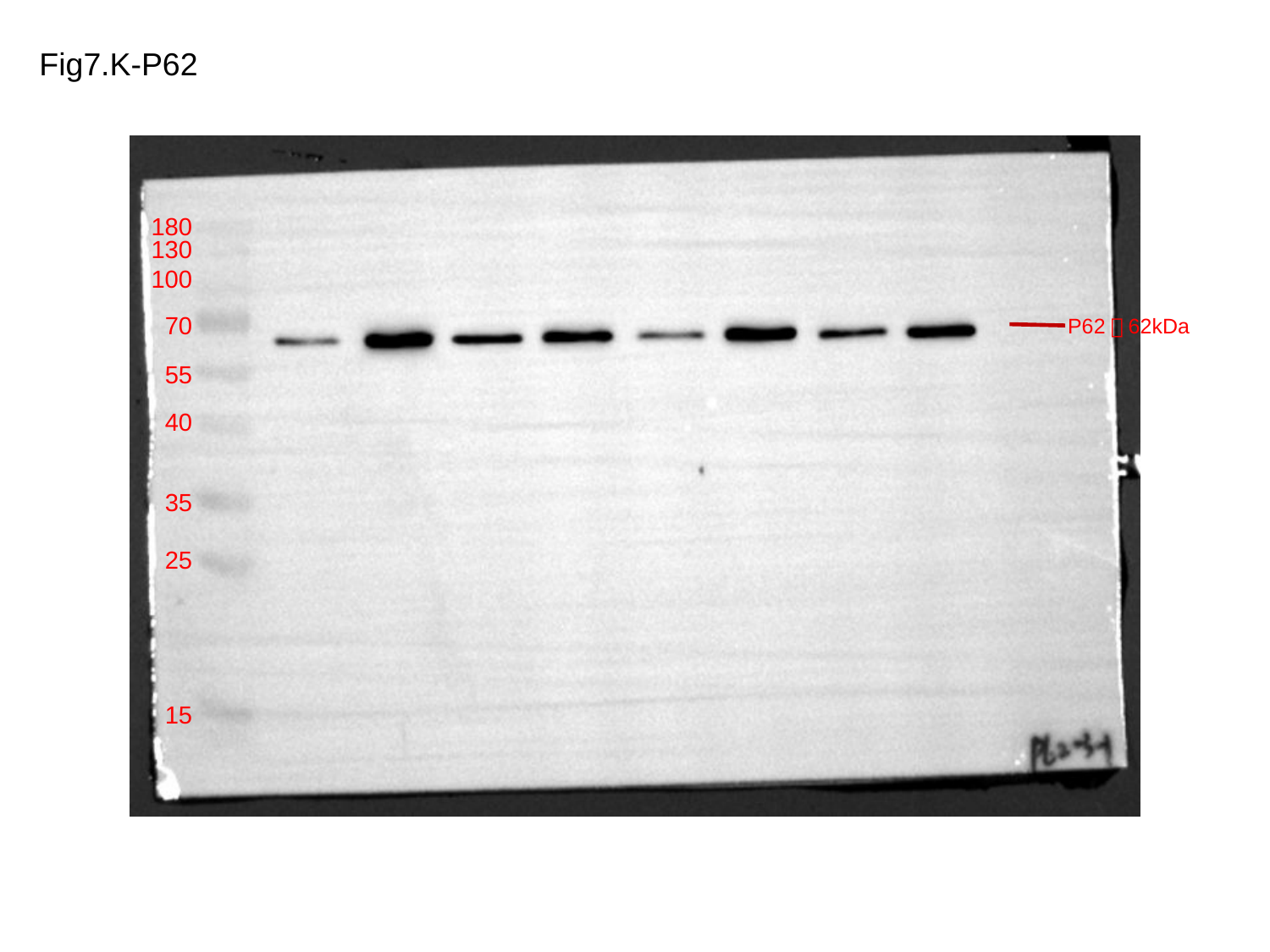

Fig7.K-P62
180
130
100
70
P62：62kDa
55
40
35
25
15

## Slide 36
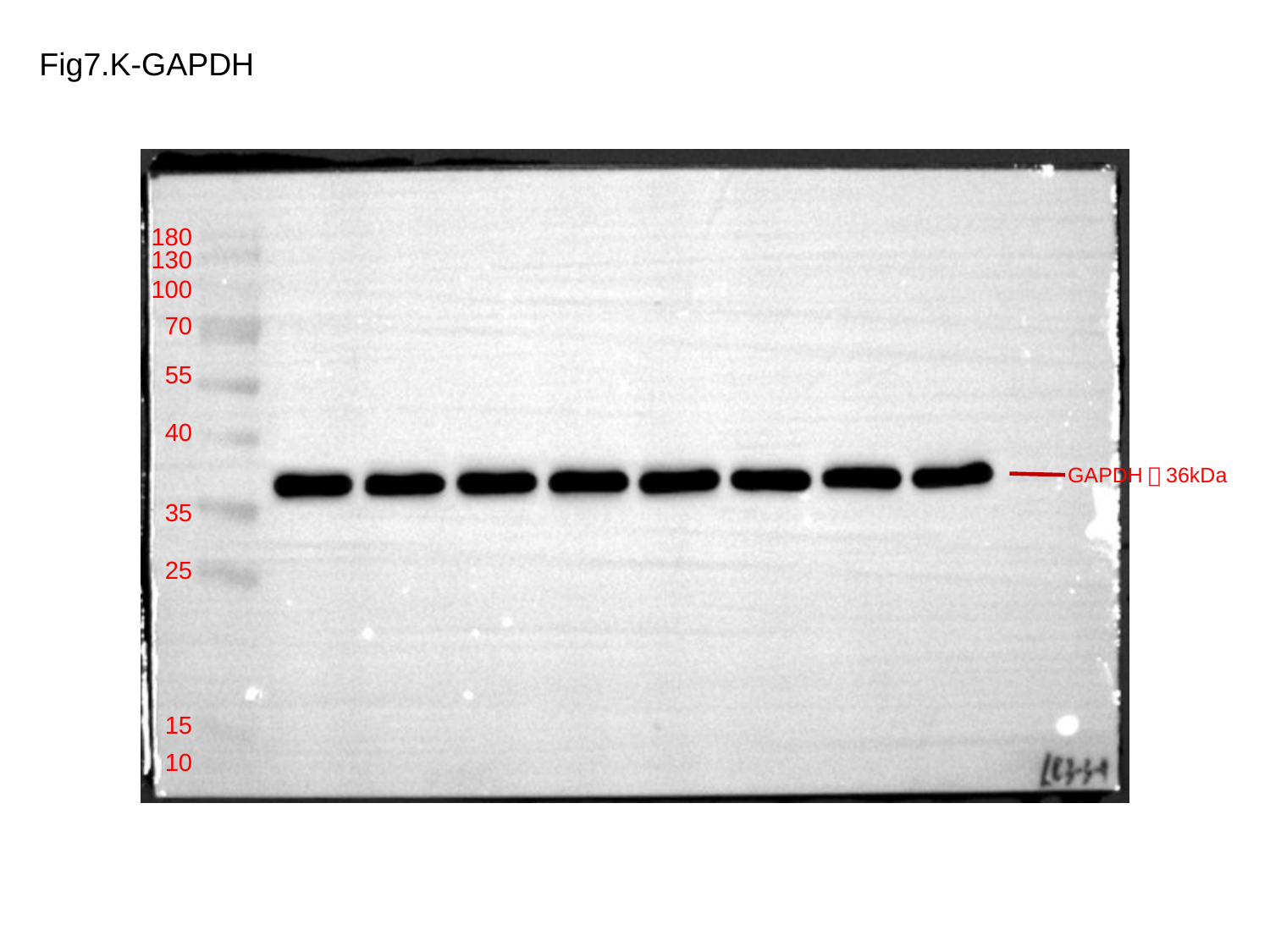

Fig7.K-GAPDH
180
130
100
70
55
40
GAPDH：36kDa
35
25
15
10
